# *Cryptosporidium parvum* and *Cryptosporidium hominis* subtypes in crab-eating macaques

**DOI:** 10.1186/s13071-019-3604-7

**Published:** 2019-07-15

**Authors:** Li Chen, Suhui Hu, Wen Jiang, Jianguo Zhao, Na Li, Yaqiong Guo, Chenghong Liao, Qian Han, Yaoyu Feng, Lihua Xiao

**Affiliations:** 1 State Key Laboratory of Bioreactor Engineering, School of Resource and Environmental, East China University of Science and Technology, Shanghai, 200237 China; 2 College of Veterinary Medicine, South China Agricultural University, Guangzhou, 510642 China; 3 Key Laboratory of Tropical Biological Resources of Ministry of Education, School of Life and Pharmaceutical Sciences, Hainan University, Haikou, 570228 Hainan China

**Keywords:** *Cryptosporidium*, *Cryptosporidium parvum*, *Cryptosporidium hominis*, Subtypes, Crab-eating macaques

## Abstract

**Background:**

Non-human primates are often infected with human-pathogenic *Cryptosporidium hominis* subtypes, but rarely with *Cryptosporidium parvum*. In this study, 1452 fecal specimens were collected from farmed crab-eating macaques (*Macaca fascicularis*) in Hainan, China during the period April 2016 to January 2018. These specimens were analyzed for *Cryptosporidium* species and subtypes by using PCR and sequence analysis of the *18S* rRNA and 60 kDa glycoprotein (*gp60*) genes, respectively.

**Results:**

Altogether, *Cryptosporidium* was detected using *18S* rRNA-based PCR in 132 (9.1%) sampled animals, with significantly higher prevalence in females (12.5% or 75/599 *versus* 6.1% or 43/706), younger animals (10.7% or 118/1102 in monkeys 1–3-years-old *versus* 4.0% or 14/350 in those over 3-years-old) and animals with diarrhea (12.6% or 46/365 *versus* 7.9% or 86/1087). Four *Cryptosporidium* species were identified, namely *C. hominis*, *C. parvum*, *Cryptosporidium muris* and *Cryptosporidium ubiquitum* in 86, 30, 15 and 1 animal, respectively. The identified *C. parvum*, *C. hominis* and *C. ubiquitum* were further subtyped by using *gp60* PCR. Among them, *C. parvum* belonged to subtypes in two known subtype families, namely IIoA14G1 (in 18 animals) and IIdA19G1 (in 2 animals). In contrast, *C. hominis* mostly belonged to two new subtype families Im and In, which are genetically related to Ia and Id, respectively. The *C. hominis* subtypes identified included ImA18 (in 38 animals), InA14 (in six animals), InA26 (in six animals), InA17 (in one animal) and IiA17 (in three animals). The *C. ubiquitum* isolates belonged to subtype family XIId. By subtype, ImA18 and IIoA14G1 were detected in animals with diarrhea whereas the remaining ones were mostly found in asymptomatic animals. Compared with *C. parvum* and *C. muris*, higher oocyst shedding intensity was observed in animals infected with *C. hominis*, especially those infected with the Im subtype family.

**Conclusions:**

Data from the study suggest that crab-eating macaques are infected with diverse *C. parvum* and *C. hominis* subtypes. The *C. parvum* IIo subtype family previously seen in rodents in China has apparently expanded its host range.

**Electronic supplementary material:**

The online version of this article (10.1186/s13071-019-3604-7) contains supplementary material, which is available to authorized users.

## Background

*Cryptosporidium* is a common gastrointestinal parasite, responsible for diarrhea in humans, non-human primates (NHPs) and ruminants [1, 2]. In humans, they are transmitted by direct person-to-person or animal-to-person contact, or indirectly through ingestion of contaminated water and food [3]. NHPs are genetically related to humans, thus are widely used as animal models in studies of human diseases. As they are in close contact with humans, laboratory NHPs have been considered potential reservoirs of human-pathogenic *Cryptosporidium* species [4].

To date, nearly 40 *Cryptosporidium* species have been established [5]. Among them, *Cryptosporidium hominis* and *Cryptosporidium parvum* are common species infecting humans [3]. The former has been identified in NHPs in some studies, most of which included its common human-pathogenic subtype families such as Ia, Ib, Id and Ie. The latter, although having a broader host range than *C. hominis*, has only been occasionally detected in NHPs, mostly by its anthroponotic subtype family IIc [4, 6, 7]. Other species occasionally detected in NHPs include *Cryptosporidium muris*, *C. andersoni*, *C. ubiquitum*, *C. meleagridis* and *C. suis* [4, 6–10]. Most of these studies were done with wild or captive animals. Thus far, there have been only two studies of *Cryptosporidium* spp. in farmed NHPs, which reported a low occurrence of *C. hominis* in crab-eating macaques (*Macaca fascicularis*) in China [11, 12].

To further examine the genetic diversity and public health potential of *Cryptosporidium* parasites in NHPs, we genotyped and subtyped these pathogens and measured the number of oocysts per gram of feces (OPG) in fecal specimens from crab-eating macaques on a commercial farm in Hainan, China.

## Methods

### Specimen collection

In April 2016, June 2017, October 2017 and January 2018, a total of 1452 fecal specimens were collected from crab-eating macaques on a commercial farm in Hainan, China. The farm was awarded accreditation from the International Association for Assessment and Accreditation of Laboratory Animal Care in 2009. More than 20,000 animals were kept at the beginning of the study. On the farm, animals were kept in individual cages (1 m^3^), with ~ 30 cages in one room of 60 m^2^. The cages were 1 m above the ground to allow stools to fall onto the floor for easy cleaning. Monkeys could interact with animals in neighboring cages. The rooms were cleaned twice daily. Male and female animals were reared in separate areas. Fresh fruit, commercial diet and clean drinking water were regularly distributed.

By gender, these animals were divided into three categories, namely 706 males, 599 females and 147 animals with missing information on the sex. By age, 1102 were 1–3-year-old monkeys and 350 were > 3 year-old adult monkeys. Because most animals over 4 years of age were sold for laboratory research, the number of adult monkeys was smaller than young monkeys on the farm. At the sampling, 365 animals had loose stools, as defined by runny fecal consistency, while the remaining 1087 monkeys had normal stools.

The fecal specimens were collected from the floor by using sterile disposable polyethylene gloves prior to room cleaning, placed into 50-ml plastic centrifuge tubes and transported to the laboratory in coolers with ice packs. The specimens were stored in 2.5% potassium dichromate solution at 4 °C for less than two months prior to DNA extraction.

### DNA extraction

About 200 mg of each fecal specimen was washed three times with distilled water by centrifugation at 2000×*g* for 10 min. Genomic DNA was isolated from the washed fecal material by using a FastDNA SPIN Kit for soil (MP Biomedicals, Santa Ana, CA, USA) and stored at − 20 °C before PCR analysis within one year.

### *Cryptosporidium* genotyping and subtyping

*Cryptosporidium* was detected by nested PCR amplification of a ~ 830-bp fragment of the *18S* rRNA gene [13]. The species present was determined by sequence analysis of the PCR products. To subtype *C. parvum* and *C. hominis*, a ~ 850-bp fragment of the 60 kDa glycoprotein (*gp60*) gene was analyzed by nested PCR and DNA sequencing [14]. Another set of *gp60* primers targeting a ~ 1000-bp fragment were used in subtyping *Cryptosporidium ubiquitum* [15]. Each specimen was analyzed by PCR at least twice, with *Cryptosporidium bovis* DNA being used as the positive control for the *18S* rRNA PCR and DNA of *C. parvum* IOWA isolate (Waterborne, Inc., New Orleans, LA, USA) as the positive control for the *gp60* PCR. A negative control with reagent water was used in each PCR analysis.

### Sequence analysis

All secondary PCR products of the *18S* rRNA and *gp60* genes were bi-directionally sequenced using the secondary PCR primers on an ABI 3730 Genetic Analyzer (Applied Biosystems, Foster City, CA, USA). Nucleotide sequences generated were assembled by using software ChromasPro v.1.32 (http://technelysium.com.au/ChromasPro.html), aligned with reference sequences from GenBank by using ClustalX (http://clustal.org). A maximum likelihood (ML) tree was constructed by using MEGA6 (https://www.megasoftware.net/). The robustness of the cluster formation was assessed by using bootstrapping with 1000 pseudo-replicates. Representative sequences generated in this study were submitted to the GenBank database under accession numbers MG952704–MG952706, MG952708–MG952714 and MK509808. Alignments of the *18S* rRNA and *gp60* sequences are presented as Additional file 1: Figure S1 and Additional file 2: Figure S2.

### Measurement of oocyst shedding intensity

A quantitative PCR (qPCR), 18S-LC2 targeting the *18S* rRNA gene but with the use of SYBR Green instead of FRET probes for detection, was used to estimate the intensity of oocyst shedding in *Cryptosporidium*-positive specimens [16]. The 20-μl qPCR mix consisted of 10 μl of 2× SYBR Green Real-time PCR Master Mix (Toyobo Co., Ltd., Osaka, Japan), 0.8 μl of 10 mg/ml bovine serum albumin, 1 μl of 10 μM primers (each), 1 μl of DNA and 6.2 μl of PCR grade water. The qPCR was conducted on a LightCycler 480 II (Roche, Indianapolis, IN, USA) as follows: 1 cycle at 95 °C for 3 min; 50 cycles at 95 °C for 5 s, 55 °C for 10 s and 72 °C for 40 s; 1 cycle at 95 °C for 10 s and 50 °C for 30 s, and 0.1 °C melt steps from 50 to 80 °C; and cooling at 40 °C for 30 s. The Ct values generated in the qPCR were used in estimating the number of oocysts per gram of feces (OPG) in *Cryptosporidium*-positive specimens, using a standard curve generated with negative fecal specimens spiked with 10^2^, 10^3^, 10^4^, 10^5^ and 10^6^ oocysts of the *C. parvum* IOWA isolate.

### Statistical analysis

The Chi-square test implemented in SPSS Statistics v.20.0 for Windows (SPSS, Inc., Chicago, IL, USA) was used to compare differences in *Cryptosporidium* prevalence between gender, age groups or animals with and without diarrhea. Student’s t-test was used to compare average OPG between *Cryptosporidium* species or subtype families. Differences with *P* < 0.05 were considered significant.

## Results

### Occurrence of *Cryptosporidium* in crab-eating macaques

A total of 132 specimens (9.1%) were positive for *Cryptosporidium* among the 1452 fecal specimens from crab-eating macaques. The prevalence of *Cryptosporidium* in the first batch of specimens (1.3%, 3/236) was significantly lower than that of the second (13.3%, 53/399; *χ*^2^ = 26.61, *df* = 1, *P* < 0.0001), third (10.9%, 64/586; *χ*^2^ = 20.93, *df* = 1, *P* < 0.0001) or fourth batch of specimens (5.2%, 12/231; *χ*^2^ = 5.78, *df* = 1, *P* = 0.0162) (Table 1).

**Table 1:** Occurrence of *Cryptosporidium* species and subtypes in crab-eating macaques in Hainan, China

Sampling	Sample size	No. positive for *Cryptosporidium* (%)	*C. parvum*		*C. hominis*		*C. ubiquitum*		*C. muris*
			No. positive	Subtype (*n*)	No. positive	Subtype (*n*)	No. positive	Subtype (*n*)	No. positive
1st (Apr. 2016)	236	3 (1.3)	–	–	1	–	–	–	2
2nd (Jun. 2017)	399	53 (13.3)	14	IIoA14G1 (9)	32	ImA18 (12); InA14 (6); IiA17 (1)	1	XIId (1)	6
3rd (Oct. 2017)	586	64 (10.9)	15	IIoA14G1 (9); IIdA19G1 (2),	49	ImA18 (26); InA26 (5); InA17 (1); In (1)	–	–	–
4th (Jan. 2018)	231	12 (5.2)	1	–	4	InA26 (1); IiA17 (2)	–	–	7
Total	1452	132 (9.1)	30	IIoA14G1 (18); IIdA19G1 (2)	86	ImA18 (38); In (1); InA14 (6); InA17 (1); InA26 (6); IiA17 (3)	1	XIId (1)	15

By sex, the prevalence of *Cryptosporidium* in females was significantly higher than in males (*χ*^2^ = 16.16, *df* = 1, *P* = 0.0001; Table 2). By age, the prevalence in animals 1–3-years-old was significantly higher than in animals above 3-years-old (*χ*^2^ = 14.46, *df* = 1, *P* = 0.0001; Table 2). By fecal consistency, the prevalence in animals with loose stools was significantly higher than in animals with normal stools (*χ*^2^ = 7.28, *df* = 1, *P* = 0.0070; Table 2).

**Table 2:** *Cryptosporidium* species and subtypes in crab-eating macaques in Hainan, China by age, sex and fecal consistency

Specimen	Sampling size	No. positive for *Cryptosporidium* (%)	*Cryptosporidium* species				*C. parvum* subtype (*n*)	*C. hominis* subtype (*n*)	*C. ubiquitum* subtype (*n*)
			No. of *C. parvum*	No. of *C. hominis*	No. of *C. muris*	No. of *C. ubiquitum*			
1–2-years-old	609	71 (11.7)	21	47	3	–	IIoA14G1 (14); IIdA19G1 (1)	ImA18 (26); InA17 (1); In (1); InA26 (4)	–
2–3-years-old	493	47 (9.5)	9	30	7	1	IIdA19G1 (1); IIoA14G1 (4)	ImA18 (12); InA14 (6)	XIId (1)
> 3-years-old	350	14 (4.0)^a^	–	9	5	–	–	IiA17 (3); InA26 (2)	–
Male	706	43 (6.1)	17	13	12	1	IIoA14G1 (12); IIdA19G1 (1)	InA26 (3)	XIId (1)
Female	599	75 (12.5)^b^	6	68	1	–	IIoA14G1 (1); IIdA19G1 (1)	ImA18 (38); In (1); InA14 (6); InA17 (1); InA26 (3); IiA17 (2)	–
Unknown sex	147	14 (11.6)	4	2			IIoA14G1 (5)	IiA17 (1)	–
Normal stools	1087	86 (7.9)	20	50	15	1	IIoA14G1 (12); IIdA19G1 (1)	ImA18 (13); InA14 (6); IiA17 (3); In (1); InA17 (1); InA26 (4)	XIId (1)
Loose stools	365	46 (12.6)^c^	10	36	–	–	IIoA14G1 (6); IIdA19G1 (1)	ImA18 (25); InA26 (2)	–
Total	1452	132 (9.1)	30	86	15	1	IIoA14G1 (18); IIdA19G1 (2)	ImA18 (38); In (1); InA14 (6); InA17 (1); InA26 (6); IiA17 (3)	XIId (1)

^a^*P* < 0.05, for above 3-years-old in comparison with 1–3-years-old

^b^*P* < 0.05, for females in comparison with male monkeys

^c^*P* < 0.05, for loose stools in comparison with normal stools

### Occurrence of *Cryptosporidium* species

Based on sequence analysis of the *18S* rRNA PCR products, four *Cryptosporidium* species were identified among the 132 positive specimens. Among them, *C. hominis* was detected in 86 specimens, with *18S* rRNA sequences identical to the reference KF679722 obtained from NHPs in China [10]. Compared with nucleotide sequences from most *C. hominis* isolates from humans, they had nine (instead of 11) consecutive T in the hypervariable region of the gene and an A to T substitution upstream from it. In contrast, *C. parvum* was detected in 30 specimens, with nucleotide sequences identical to the reference MF074701 obtained from dairy calves in China [17]. Between the remaining two *Cryptosporidium* species, *C. muris* was identified in 15 specimens while *C. ubiquitum* in one specimen, producing *18S* rRNA sequences that were identical to KF419208 obtained from brown rats in China and KT027449 obtained from eastern gray squirrels in the USA, respectively [18, 19].

### Occurrence of *C. parvum*, *C. hominis* and *C. ubiquitum* subtypes

Among the 117 specimens that were positive for *C. parvum-*, *C. hominis-* and *C. ubiquitum-*positive specimens, 76 were successfully subtyped at the *gp60* locus, namely 20 of the 30 *C. parvum-*positive specimens, 55 of the 86 *C. hominis-*positive specimens and the only *C. ubiquitum-*positive specimen (Table 1). The remaining 15 specimens that were positive for *C. muris* did not produce the expected *gp60* PCR products.

Two known subtypes were identified among *C. parvum*-positive specimens, namely IIoA14G1 (18/20) and IIdA19G1 (2/20), with nucleotide sequences identical to the reference sequence KC885906 and KJ802724, respectively [20, 21].

**Fig. 1:**
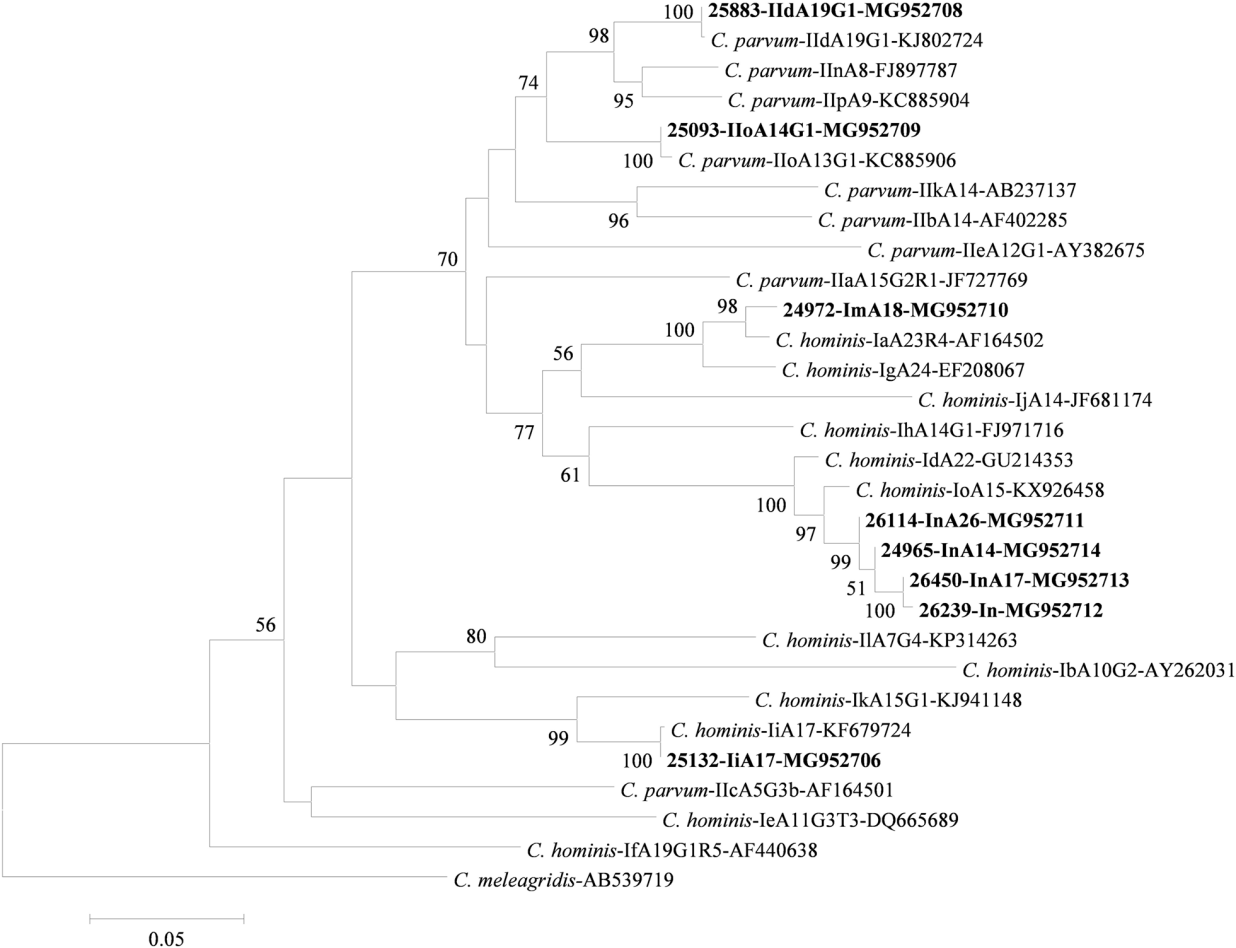
Phylogenetic relationship of *Cryptosporidium parvum* and *Cryptosporidium hominis* subtypes as inferred by a maximum likelihood (ML) analysis of nucleotide sequences of the 60-KDa glycoprotein gene. Bootstrap values were obtained using 1000 pseudo-replicates, with values above 50% being shown on nodes. Bolded sequences are from subtypes found in this study, with specimen IDs being shown before the subtype names and GenBank accession numbers. The tree is rooted with a *gp60* sequence (AB539719) from *C. meleagridis*

Six subtypes were identified within *C. hominis*: one subtype of a new subtype family Im; four subtypes of a new subtype family In; and one subtype of the known subtype family Ii. The new subtype family Im had a nucleotide sequence that was very similar to the Ia family (GenBank: AF164502), with 15 (2%) nucleotide substitutions in the non-repeat region. Only one subtype, ImA18, was present within the subtype family, and was seen in 38 specimens. In contrast, the new family In had nucleotide sequences that were similar to the Id family (GenBank: GU214353), with 28 (3%) nucleotide substitutions in the non-repeat region. It included subtypes InA14, InA17 and InA26, which were detected in 6, 1 and 6 specimens, respectively. One sequence from the In subtype family had un-readable trinucleotide region. As expected, in phylogenetic analysis, Im and In clustered with Ia and Id subtype families, respectively (Fig. 1). The IiA17 subtype in the Ii subtype family had a nucleotide sequence identical to KF679724 [10], and was detected in 3 specimens.

The only *C. ubiquitum* specimen was subtyped as XIId, with a nucleotide sequence identical to the reference sequence JX412924, which was seen in rodents and humans in the USA [15].

### Occurrence of *Cryptosporidium* subtypes by sampling date, gender, age and fecal consistency

The two common subtypes ImA18 (38/55) and IIoA14G1 (18/20) were only found at the second and third sampling (Table 1). Subtype ImA18 was only detected in females (38/53), while IIoA14G1 was the dominant subtype in males (12/17). By age, these two subtypes were almost exclusively found in 1–3-year-old animals. By fecal consistency, ImA18 was more likely detected in animals with loose stools than animals with normal stools (*χ*^2^ = 13.62, *df* = 1, *P* = 0.000). IIoA14G1, on the other hand, was detected in animals with the two types of fecal consistency at similar frequency (6/34 *vs* 12/42). Other subtypes in this study were mostly found in animals with normal stools (Table 2).

### Oocyst shedding intensity of different *Cryptosporidium* species and *C. hominis* subtypes

**Fig. 2:**
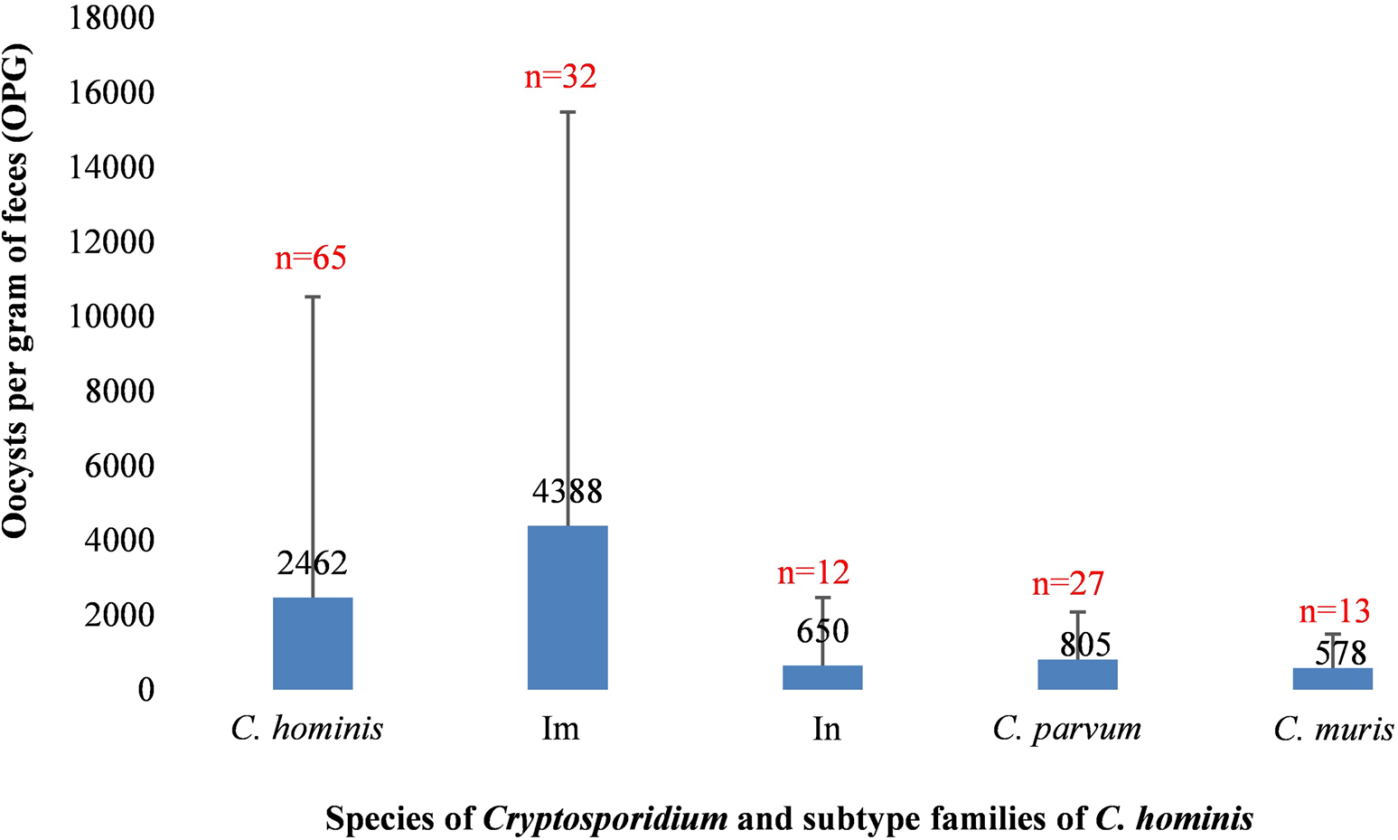
Oocysts per gram of feces in specimens of dominant *Cryptosporidium* species and *Cryptosporidium hominis* subtypes in crab-eating macaques in Hainan, China. The sample size (*n*) for each species or subtype is specified above the bar. None of the differences reached statistical significance (*P* > 0.05)

By species, the average number of oocysts per gram of feces (OPG) of 65 *C. hominis-*positive specimens was 2462, which was higher than that of 27 *C. parvum-*positive specimens (805; *t*_(90)_ = 1.05, *P* = 0.296), 13 *C. muris-*positive specimens (578; *t*_(76)_ = 1.77, *P* = 0.081) and the only *C. ubiquitum*-positive specimen (65). By *C. hominis* subtype, the average OPG of 32 Im-positive specimens was higher than that of 12 In-positive specimens (*t*_(42)_ = 1.93, *P* = 0.063) (Fig. 2).

## Discussion

In this study, *Cryptosporidium* was commonly found in crab-eating macaques on a large commercial farm. The 9.1% (132/1452) prevalence is higher than the prevalence reported in NHPs in Shaanxi (7.0%, 6/86), Anhui (2.3%, 1/44), Guangxi (0.5%, 1/205) and southern provinces (0.7%, 19/2660) of China [8, 10, 11, 22]. However, it is similar to the prevalence in free-range rhesus monkeys in a public park in Guizhou (10.9%, 45/411) [7]. Elsewhere in the world, it is higher than the prevalence of NHPs found in Uganda (6.7%, 10/149), Rwanda (4.0%, 4/100), Madagascar (4.0%, 1/25), Kenya (2.6%, 6/235) and Thailand (1.0%, 2/200) [23–28], but slightly lower than the prevalence in Tanzania (16.0%, 21/131) [9].

A diverse range of *Cryptosporidium* species were identified in crab-eating macaques in the present study, namely *C. hominis* (in 86 animals), *C. parvum* (in 30 animals), *C. muris* (in 15 animals) and *C. ubiquitum* (in one animal). Similarly, studies conducted prior to the present study had identified nine *Cryptosporidium* species in a total of 163 *Cryptosporidium*-positive fecal specimens from NHPs, namely *C. hominis* (50.3%, 82/163), *C. muris* (21.5%, 35/163), *C. parvum* (16.0%, 26/163), *C. suis* (3.7%, 6/163), *C. andersoni* (3.1%, 5/163), *C. ubiquitum* (3.1%, 5/163), *C. meleagridis* (1.2%, 2/163), *C. bovis* (0.6%, 1/163) and *C. felis* (0.6%, 1/163) [4, 6–11, 15, 22–38]. Nevertheless, the frequency (30/132 or 22.7%) of the zoonotic *C. parvum* in crab-eating macaques in the present study is much higher than in previous studies. Previously, most *C. parvum* infections were found in NHPs in Uganda and China [7, 8, 22, 27, 28, 33, 36, 37, 39]. The large number of *C. muris* infections (15/132) in this study support previous observation of this zoonotic species in NHPs elsewhere [4, 10, 22, 24, 38, 40].

The *C. parvum* subtypes found in this study appear to be unique. Previously, *C. parvum* reported in NHPs belonged to the common subtype families IIc and IId [7, 8]. However, in this study, the rare subtype family IIo was detected in NHPs for the first time. Compared with common *C. parvum* subtype families, the IIo subtype family has two nucleotide substitutions in the *18S* rRNA gene. Two subtypes of this subtype family, IIoA16G1 and IIoA13G1, were detected in humans traveling to Thailand as well as bamboo rats in China [20, 41, 42]. The large number of IIoA14G1 infections in this study indicates that the zoonotic subtype family has expanded its range from rodents to NHPs in southern China. In contrast, the IIdA19G1 subtype found in a few monkeys is one of the two common IId subtypes infecting ruminants and rodents in China [17, 43–48]. The detection of both IIoA14G1 and IIdA19G1 in this study supports the previous conclusion that rodents are important reservoirs of *C. parvum* in China, and suggests that the rodents might play an important role in the transmission of these pathogens in NHPs [5].

Similarly, *C. hominis* detected in this study belongs to highly divergent subtype families. Most of *C. hominis* isolates detected belong to two new subtype families Im and In, while the remaining ones belong to the NHP-adapted subtype family Ii. The sequence differences between Im and Ia (15 nucleotides) or In and Id (28 nucleotides) are greater than those between IIe and IIm, which differ from each other by 13 nucleotides in the non-repeat region. In previous studies, human-pathogenic subtype families Ia, Ib, Id, Ie and If were commonly detected in NHPs [7, 9, 10, 22, 23]. The Im and In detected in this study appear to be genetically divergent from them, as they also differ from them by two nucleotides at the *18S* rRNA locus. Among them, the In subtype family was previously seen in a farmed crab-eating macaque in Guangxi, although it was mistakenly named as IdA14 [11]. Similarly, IiA17 of the Ii subtype family has the same sequence variations at the *18S* rRNA gene and had been detected in only a few NHPs [5, 10, 49]. The IiA14 detected in one baboon in Kenya previously [23] was actually IjA14 (JF681174) [50]. As Ii subtype family has been found in some human cases, it is apparently zoonotic [5, 51].

There were apparent differences in the transmission of the two common subtypes of *C. parvum* and *C. hominis*, IIoA14G1 and ImA18. By sex, ImA18 was only found in females, while IIoA14G1 was the major subtype in males (Table 2). However, this could be due to the fact that females and males were kept in different areas in the facility. By fecal consistency, ImA18 and IIoA14G1 were overrepresented in animals with loose stools (in 31/34 subtyped specimens from animals with loose stool, compared with in 25/41 subtyped specimens from animals with normal stools), while other subtypes were mainly found in animals with normal stools (Table 2). In addition, the number of monkeys infected with *C. hominis* (*n* = 86) was higher than those infected with *C. parvum* (*n* = 30) and *C. muris* (*n* = 15). Among them, *C. hominis* had higher oocyst shedding intensity than *C. parvum* and *C. muris*, while within the former, the Im subtype family had higher oocyst shedding intensity than the In subtype family (*P* > 0.05, Fig. 2). Differences in oocyst shedding indicate that virulence is associated to the type of species and subtypes infecting NHPs.

## Conclusions

In conclusion, diverse *Cryptosporidium* species and subtypes are present in farmed crab-eating macaques in Hainan, China. The *C. hominis* subtype families (Im, In and Ii) appear to be NHP-adapted, while *C. parvum* subtype family IIo appears to have expanded its host range. More studies should be conducted in other regions to monitor the dispersal of these divergent *C. parvum* and *C. hominis* subtypes. Comparative genomic analysis should be conducted to further understand their genetic uniqueness.

## Additional files

**Additional file 1: Figure S1.** Alignment of nucleotide sequences of the *18S* rRNA gene of *Cryptosporidium* species.

**Additional file 2: Figure S2.** Alignment of nucleotide sequences of the 60 kDa glycoprotein gene of *Cryptosporidium parvum* and *Cryptosporidium hominis*.

## Data Availability

The data supporting the conclusions of this article are included within the article and its additional files. Representative sequences generated in this study were submitted to the GenBank database under accession numbers MG952704–MG952706, MG952708–MG952714 and MK509808.

## Abbreviations

PCR: polymerase chain reaction
qPCR: quantitative PCR
NHPs: non-human primates
*gp60*: 60 kDa glycoprotein gene
OPG: oocysts per gram of feces
