# Supplementary material for: Cryptosporidium parvum and Cryptosporidium hominis subtypes in crab-eating macaques
Source: Parasit Vectors. 2019 Jul 15;12:350. doi: 10.1186/s13071-019-3604-7 (PMC6631616; doi:10.1186/s13071-019-3604-7)
Supplement: Supplementary file 1 — Additional file 1: Figure S1. Alignment of nucleotide sequences of the 18S rRNA gene of Cryptosporidium species. [file 13071_2019_3604_MOESM1_ESM.pdf]

Additional file 1: Figure S1. Alignment of nucleotide sequences of the 18S rRNA gene of *Cryptosporidium* species.

```
                       ....|....| ....|....| ....|....| ....|....| ....|....| ....|....|
                                10         20         30         40         50         60
25330-C.ubiquitum      ---------- ---------- ---------- ---------- ---------- ----------
C_ubiquitum_KC608030   ---------- ---------- ---------- ---------- ----GGTGAT TCATAATAAC
C_ubiquitum_KC962124   ---------- ---------- ---------- -CCAATTT-A ATTTGGTGAT TCATAATAAC
C_ubiquitum_AB697056   ---------- ---------- ---------- -CCAATTT-A ATTTGGTGAT TCATAATAAC
C_ubiquitum_KU531665   ---------- ---------- ---------- ---------- ---------- ----------
C_felis_JQ312664       ---------- ---------- ---------- -----TTT-T TTTTGGTGAC TCATAATAAC
C_felis_AJ493211       ---------- ---------- ---------- ATTATTTT-T TTTTGGTGAC TCATAATAAC
C_felis_JN833576       ---------- ---------- ---------- ----TTTT-T TTTTGGTGAC TCATAATAAC
C_felis_AF159113       ---------- ---------- --------CC AATATTTT-T TTTTGGTGAC TCATAATAAC
C_felis_KT749819       ---------- ---------- --------CC AATATTTT-T TTTTGGTGAC TCATAATAAC
C_felis_FJ707310       ---------- ---------- --------CC AATATTTT-T TTTTGGTGAC TCATAATAAC
C_felis_KM977642       ---------- ---------- --------CC AATATTTT-T TTTTGGTGAC TCATAATAAC
C_muris_GU319781       ---------- ---------- ---------- --CCAATG-A GCTTGGTGAT TCATAATAAC
C_muris_KF419208       ---------- ---------- ---------- --CCAATG-A GCTTGGTGAT TCATAATAAC
C_muris_EU553592       ---------- ---------- ---------- --CCAATG-A GCTTGGTGAT TCATAATAAC
C_muris_GU319783       ---------- ---------- ---------- --CCAATG-A GCTTGGTGAT TCATAATAAC
C_muris_EU245045       ---------- ---------- ---------- --CCAATG-A GCTTGGTGAT TCATAATAAC
C_muris_GQ227706       ---------- ---------- ---------- --CCAATG-A GCTTGGTGAT TCATAATAAC
C_muris_EU156446       ---------- ---------- ---------- --CCAATG-A GCTTGGTGAT TCATAATAAC
C_muris_AY642591       ---------- ---------- ---------- --CCAATG-A GCTTGGTGAT TCATAATAAC
C_muris_KY490555       ---------- ---------- ---------- ---------- ---TGGTGAT TCATAATAAC
23767-C.muris          ---------- ---------- ---------- ---------- ---------- ----------
C_andersoni_AB449819   ---------- ---------- ---------- ---CANTG-A GCNTGGTGAN NCATNNTAAN
C_andersoni_EF613341   ---------- ---------- ---------- ---------- ---------- ----------
C_andersoni_KF826306   ---------- ---------- ---------- --CCAATG-A GCTTGGTGAT TCATAATAAC
C_andersoni_KT922229   ---------- ---------- ---------- --CCAATG-A GCTTGGTGAT TCATAATAAC
C_andersoni_KT884487   ---------- ---------- ---------- --CCAATG-A GCTTGGTGAT TCATAATAAC
C_andersoni_LC012014   ---------- ---------- ---------- --CCAATG-A GCTTGGTGAT TCATAATAAC
C_andersoni_AB449816   ---------- ---------- ---------- --CCAATG-A GCTTGGTGAT TCATAATAAC
C_andersoni_AY954886   ---------- ---------- ---------- --CCAATG-A GCTTGGTGAT TCATAATAAC
C_andersoni_AY954885   ---------- ---------- ---------- --CCAATG-A GCTTGGTGAT TCATAATAAC
C_andersoni_AB089285   ---------- ---------- ---------- --CCAATG-A GCTTGGTGAT TCATAATAAC
C_andersoni_LC012013   ---------- ---------- ---------- --CCAATG-A GCTTGGTGAT TCATAATAAC
C_andersoni_KF826307   ---------- ---------- ---------- --CCAATG-A GCTTGGTGAT TCATAATAAC
C_andersoni_KF826305   ---------- ---------- ---------- --CCAATG-A GCTTGGTGAT TCATAATAAC
C_andersoni_KF826304   ---------- ---------- ---------- --CCAATG-A GCTTGGTGAT TCATAATAAC
C_andersoni_EU245042   ---------- ---------- ---------- --CCAATG-A GCTTGGTGAT TCATAATAAC
C_andersoni_KT922228   ---------- ---------- ---------- --CCAATG-A GCTTGGTGAT TCATAATAAC
C_andersoni_KT884488   ---------- ---------- ---------- --CCAATG-A GCTTGGTGAT TCATAATAAC
C_andersoni_AB449817   ---------- ---------- ---------- --CCAATG-A GCTTGGTGAT TCATAATAAC
C_andersoni_KT922230   ---------- ---------- ---------- --CCAATG-A GCTTGGTGAT TCATAATAAC
C_hominis_KF826315     ---------- ---------- ---------- ---------- ----GGTGAC TCATAATAAC
C_hominis_HQ149022     ---------- ---------- ---------- ---------- ---------- ----AATAAC
C_hominis_KR296813     ---------- ---------- ---------- --CCAATA-T AATTGGTGAC TCATAATAAC
C_hominis_AF112569     ---------- ---------- ---------- --CCAATA-T AATTGGTGAC TCATAATAAC
C_hominis_KF679723     ---------- ---------- ---------- --CCAATA-T AATTGGTGAC TCATAATAAC
24937-C.hominis        ---------- --------TA TTAGATAAAG AACCAATA-T AATTGGTGAC TCATAATAAC
C_parvum_KP204486      ---------- ---------- ---------- --CCAATA-T AATTGGTGAC TCATAATAAC
C_parvum_KM012042      ---------- ---------- ---------- --CCAATA-T AATTGGTGAC TCATAATAAC
C_parvum_AY268582      ---------- ---------- ---------- ---------- ----GGTGAC TCATAATAAC
C_parvum_KM012045      ---------- ---------- ---------- --CCAATA-T AATTGGTGAC TCATAATAAC
C_parvum_KM012043      ---------- ---------- ---------- --CCAATA-T AATTGGTGAC TCATAATAAC
C_parvum_AF108864      ---------- ---------- ---------- --CCAATA-T AATTGGTGAC TCATAATAAC
C_parvum_KM085018      ---------- ---------- ---------- ---------- -ATTGGTGAC TCATAATAAC
C_parvum_LC01201       ---------- ---------- ---------- --CCAATA-T AATTGGTGAC TCATAATAAC
C_parvum_KJ808688      ---------- ---------- ---------- --CCAATA-T AATTGGTGAC TCATAATAAC
C_parvum_LC012016      ---------- ---------- ---------- --CCAATA-T AATTGGTGAC TCATAATAAC
C_parvum_KM012040      ---------- ---------- ---------- --CCAATA-T AATTGGTGAC TCATAATAAC
C_parvum_KJ808687      ---------- ---------- ---------- --CCAATA-T AATTGGTGAC TCATAATAAC
C_parvum_KM012044      ---------- ---------- ---------- --CCAATA-T AATTGGTGAC TCATAATAAC
C_parvum_KP334136      ---------- ---------- ---------- --CCAATA-T AATTGGTGAC TCATAATAAC
C_parvum_KJ808689      ---------- ---------- ---------- --CCAATA-T AATTGGTGAC TCATAATAAC
```

```
C_parvum_KM012046          ---------- ---------- ---------- --CCAATA-T AATTGGTGAC TCATAATAAC
C_parvum_LC012015          ---------- ---------- ---------- --CCAATA-T AATTGGTGAC TCATAATAAC
C_parvum_K16X154           ---------- ---------- ---------- --CCAATA-T AATTGGTGAC TCATAATAAC
C_parvum_KJ808691          ---------- ---------- ---------- --CCAATA-T AATTGGTGAC TCATAATAAC
C_parvum_KU892559          ---------- ---------- ---------- --CCAATA-T AATTGGTGAC TCATAATAAC
C_parvum_AB968048          ---------- ---------- ---------- --CCAATA-T AATTGGTGAC TCATAATAAC
C_parvum_KJ808690          ---------- ---------- ---------- --CCAATA-T AATTGGTGAC TCATAATAAC
C_parvum_KJ808692          ---------- ---------- ---------- --CCAATA-T AATTGGTGAC TCATAATAAC
C_parvum_AF093493          ---------- ---------- ---------- --CCAATA-T AATTGGTGAC TCATAATAAC
C_parvum_EU553550          ---------- ---------- ---------- --CCAATA-T AATTGGTGAC TCATAATAAC
C_parvum_EU553557          ---------- ---------- ---------- --CCAATA-T AATTGGTGAC TCATAATAAC
C_parvum_KJ808693          ---------- ---------- ---------- --CCAATA-T AATTGGTGAC TCATAATAAC
C_parvum_KJ808694          ---------- ---------- ---------- --CCAATA-T AATTGGTGAC TCATAATAAC
C_parvum_KJ808695          ---------- ---------- ---------- --CCAATA-T AATTGGTGAC TCATAATAAC
25115-C.parvum             ---------- ---------- ---------- ---------- ---------- ----------
C_parvum_KP004204          ---------- ---------- ---------- ---------- -----GTGAC TCATAATAAC
C_parvum_KP004203          ---------- ---------- ---------- ---------- ---------C TTATAATAAC
C_parvum_AB271070          ---------- ---------- ---------- ---------- ---------- ----------


                           ....|....| ....|....| ....|....| ....|....| ....|....| ....|....|
                                   70         80         90        100        110        120
25330-C.ubiquitum          --------AT CACA-----T TTATATGTGA CATATCATTC AAGTTTCTGA CC-TATCAGC
C_ubiquitum_KC608030       TTT-ACGGAT CACA-----T TTATATGTGA CATATCATTC AAGTTTCTGA CC-TATCAGC
C_ubiquitum_KC962124       TTT-ACGGAT CACA-----T TTATATGTGA CATATCATTC AAGTTTCTGA CC-TATCAGC
C_ubiquitum_AB697056       TTT-ACGGAT CACA-----T TTATATGTGA CATATCATTC AAGTTTCTGA CC-TATCAGC
C_ubiquitum_KU531665       ---------- ---------- ---------- ---------- ---------- ----------
C_felis_JQ312664           TTT-ACGGAT CACAATTAT- -ATTTTGTGA CATATCATTC ATGTTTCTGA CC-TATCAGC
C_felis_AJ493211           TTT-ACGGAT CACAATAATT TATTTTGTGA CATATCATTC AAGTTTCTGA CC-TATCAGC
C_felis_JN833576           TTT-ACGGAT CACAATTT-- -ATTTTGTGA CATATCATTC AAGTTTCTGA CC-TATCAGC
C_felis_AF159113           TTT-ACGGAT CACAATAATT TATTTTGTGA CATATCATTC AAGTTTCTGA CC-TATCAGC
C_felis_KT749819           TTT-ACGGAT CACAATAATT TATTTTGTGA CATATCATTC AAGTTTCTGA CC-TATCAGC
C_felis_FJ707310           TTT-ACGGAT CACAATAATT TATTTTGTGA CATATCATTC AAGTTTCTGA CC-TATCAGC
C_felis_KM977642           TTT-ACGGAT CACAATAATT TATTTTGTGA CATATCATTC AAGTTTCTGA CC-TATCAGC
C_muris_GU319781           TTT-ACGGAT CGCA----TC TCTGATGCGA CATATCATTC AAGTTTCTGA CC-TATCAGC
C_muris_KF419208           TTT-ACGGAT CGCA----TC TCTGATGCGA CATATCATTC AAGTTTCTGA CC-TATCAGC
C_muris_EU553592           TTT-ACGGAT CGCA----TC TCTGATGCGA CATATCATTC AAGTTTCTGA CC-TATCAGC
C_muris_GU319783           TTT-ACGGAT CGCA----TC TCTGATGCGA CATATCATTC AAGTTTCTGA CC-TATCAGC
C_muris_EU245045           TTT-ACGGAT CGCA----TC TCTGATGCGA CATATCATTC AAGTTTCTGA CC-TATCAGC
C_muris_GQ227706           TTT-ACGGAT CGCA----TC TCTGATGCGA CATATCATTC AAGTTTCTGA CC-TATCAGC
C_muris_EU156446           TTT-ACGGAT CGCA----TC TCTGATGCGA CATATCATTC AAGTTTCTGA CC-TATCAGC
C_muris_AY642591           TTT-ACGGAT CGCA----TC TTTGATGCGA CATATCATTC AAGTTTCTGA CC-TATCAGC
C_muris_KY490555           TTTTACGGAT CGCA----TC TCTGATGCGA CATATCATTC AAGTTTCTGA CC-TATCAGC
23767-C.muris              ---------- ---------- ---------- ---------- ---------- ---------C
C_andersoni_AB449819       TTT-ACGGAT CGCA----TC TCTGATGCGA CATATCATTC AAGTTTCTGA CC-TATCAGC
C_andersoni_EF613341       ---------- ---------- ---------- ---------- ---------- ----------
C_andersoni_KF826306       TTT-ACGGAT CGCA----TC TCTGATGCGA CATATCATTC AAGTTTCTGA CC-TATCAGC
C_andersoni_KT922229       TTT-ACGGAT CGCA----TC TCTGATGCGA CATATCATTC AAGTTTCTGA CC-TATCAGC
C_andersoni_KT884487       TTT-ACGGAT CGCA----TC TCTGATGCGA CATATCATTC AAGTTTCTGA CC-TATCAGC
C_andersoni_LC012014       TTT-ACGGAT CGCA----TC TCTGATGCGA CATATCATTC AAGTTTCTGA CC-TATCAGC
C_andersoni_AB449816       TTT-ACGGAT CGCA----TC TCTGATGCGA CATATCATTC AAGTTTCTGA CC-TATCAGC
C_andersoni_AY954886       TTT-ACGGAT CGCA----TC TCTGATGCGA CATATCATTC AAGTTTCTGA CC-TATCAGC
C_andersoni_AY954885       TTT-ACGGAT CGCA----TC TCTGATGCGA CATATCATTC AAGTTTCTGA CC-TATCAGC
C_andersoni_AB089285       TTT-ACGGAT CGCA----TC TCTGATGCGA CATATCATTC AAGTTTCTGA CC-TATCAGC
C_andersoni_LC012013       TTT-ACGGAT CGCA----TC TCTGATGCGA CATATCATTC AAGTTTCTGA CC-TATCAGC
C_andersoni_KF826307       TTT-ACGGAT CGCA----TC TCTGATGCGA CATATCATTC AAGTTTCTGA CC-TATCAGC
C_andersoni_KF826305       TTT-ACGGAT CGCA----TC TCTGATGCGA CATATCATTC AAGTTTCTGA CC-TATCAGC
C_andersoni_KF826304       TTT-ACGGAT CGCA----TC TCTGATGCGA CATATCATTC AAGTTTCTGA CC-TATCAGC
C_andersoni_EU245042       TTT-ACGGAT CGCA----TC TCTGATGCGA CATATCATTC AAGTTTCTGA CC-TATCAGC
C_andersoni_KT922228       TTT-ACGGAT CGCA----TC TCTGATGCGA CATATCATTC AAGTTTCTGA CC-TATCAGC
C_andersoni_KT884488       TTT-ACGGAT CGCA----TC TCTGATGCGA CATATCATTC AAGTTTCTGA CC-TATCAGC
C_andersoni_AB449817       TTT-ACGGAT CGCA----TC TCTGATGCGA CATATCATTC AAGTTTCTGA CC-TATCAGC
C_andersoni_KT922230       TTT-ACGGAT CGCA----TT TCTGATGCGA CATATCATTC AAGTTTCTGA CC-TATCAGC
C_hominis_KF826315         TTT-ACGGAT CACAA----- -TTAATGTGA CATATCATTC AAGTTTCTGA CC-TATCAGC
C_hominis_HQ149022         TTT-ACGGAT CACAA----- -TTAATGTGA CATATCATTC AAGTTTCTGA CC-TATCAGC
C_hominis_KR296813         TTT-ACGGAT CACAA----- -TTAATGTGA CATATCATTC AAGTTTCTGA CC-TATCAGC
C_hominis_AF112569         TTT-ACGGAT CACAA----- -TTAATGTGA CATATCATTC AAGTTTCTGA CC-TATCAGC
C_hominis_KF679723         TTT-ACGGAT CACAA----- -TTAATGTGA CATATCATTC AAGTTTCTGA CC-TATCAGC
24937-C.hominis            TTT-ACGGAT CACAA----- -TTAATGTGA CATATCATTC AAGTTTCTGA CC-TATCAGC
C_parvum_KP204486          TTT-ACGGAT CACAT----- -TAAATGTGA CATATCATTC AAGTTTCTGA CC-TATCAGC
C_parvum_KM012042          TTT-ACGGAT CACAT----- -TAAATGTGA CATATCATTC AAGTTTCTGA CC-TATCAGC
```

```
C_parvum_AY268582         TTT-ACGGAT CACAT----- -TAAATGTGA CATATCATTC AAGTTTCTGA CC-TATCAGC
C_parvum_KM012045         TTT-ACGGAT CACAT----- -TAAATGTGA CATATCATTC AAGTTTCTGA CC-TATCAGC
C_parvum_KM012043         TTT-ACGGAT CACAT----- -TAAATGTGA CATATCATTC AAGTTTCTGA CC-TATCAGC
C_parvum_AF108864         TTT-ACGGAT CACAT----- -TAAATGTGA CATATCATTC AAGTTTCTGA CC-TATCAGC
C_parvum_KM085018         TTT-ACGGAT CACAT----- -TAAATGTGA CATATCATTC AAGTTTCTGA CC-TATCAGC
C_parvum_LC01201          TTT-ACGGAT CACAT----- -TAAATGTGA CATATCATTC AAGTTTCTGA CC-TATCAGC
C_parvum_KJ808688         TTT-ACGGAT CACAT----- -TAAATGTGA CATATCATTC AAGTTTCTGA CC-TATCAGC
C_parvum_LC012016         TTT-ACGGAT CACAT----- -TAAATGTGA CATATCATTC AAGTTTCTGA CC-TATCAGC
C_parvum_KM012040         TTT-ACGGAT CACAT----- -TAAATGTGA CATATCATTC AAGTTTCTGA CC-TATCAGC
C_parvum_KJ808687         TTT-ACGGAT CACAT----- -TAAATGTGA CATATCATTC AAGTTTCTGA CC-TATCAGC
C_parvum_KM012044         TTT-ACGGAT CACAT----- -TAAATGTGA CATATCATTC AAGTTTCTGA CC-TATCAGC
C_parvum_KP334136         TTT-ACGGAT CACAT----- -TAAATGTGA CATATCATTC AAGTTTCTGA CC-TATCAGC
C_parvum_KJ808689         TTT-ACGGAT CACAT----- -TAAATGTGA CATATCATTC AAGTTTCTGA CC-TATCAGC
C_parvum_KM012046         TTT-ACGGAT CACAT----- -TAAATGTGA CATATCATTC AAGTTTCTGA CC-TATCAGC
C_parvum_LC012015         TTT-ACGGAT CACAT----- -TAAATGTGA CATATCATTC AAGTTTCTGA CC-TATCAGC
C_parvum_K16X154          TTT-ACGGAT CACAT----- -TAAATGTGA CATATCATTC AAGTTTCTGA CC-TATCAGC
C_parvum_KJ808691         TTT-ACGGAT CACAT----- -TAAATGTGA CATATCATTC AAGTTTCTGA CC-TATCAGC
C_parvum_KU892559         TTT-ACGGAT CACAT----- -TAAATGTGA CATATCATTC AAGTTTCTGA CC-TATCAGC
C_parvum_AB968048         TTT-ACGGAT CACAT----- -TAAATGTGA CATATCATTC AAGTTTCTGA CC-TATCAGC
C_parvum_KJ808690         TTT-ACGGAT CACAT----- -TAAATGTGA CATATCATTC AAGTTTCTGA CC-TATCAGC
C_parvum_KJ808692         TTT-ACGGAT CACAT----- -TAAATGTGA CATATCATTC AAGTTTCTGA CC-TATCAGC
C_parvum_AF093493         TTT-ACGGAT CACAT----- -TAAATGTGA CATATCATTC AAGTTTCTGA CC-TATCAGC
C_parvum_EU553550         TTT-ACGGAT CACAT----- -TAAATGTGA CATATCATTC AAGTTTCTGA CC-TATCAGC
C_parvum_EU553557         TTT-ACGGAT CACAT----- -TAAATGTGA CATATCATTC AAGTTTCTGA CC-TATCAGC
C_parvum_KJ808693         TTT-ACGGAT CACAT----- -TAAATGTGA CATATCATTC AAGTTTCTGA CC-TATCAGC
C_parvum_KJ808694         TTT-ACGGAT CACAT----- -TAAATGTGA CATATCATTC AAGTTTCTGA CC-TATCAGC
C_parvum_KJ808695         TTT-ACGGAT CACAT----- -TAAATGTGA CATATCATTC AAGTTTCTGA CC-TATCAGC
25115-C.parvum            ---------- ---------- -----TGTGA CATATCATTC AAGTTTCTGA CC-TATCAGC
C_parvum_KP004204         TTT-ACGGAT CACAT----- -TAAATGTGA CATATCATTC AAGTTTCTGA CC-TATCAGC
C_parvum_KP004203         TTTTACGGAT CACAT----- TTAAATGTGA CATATCATTC AAGTTTCTGA CC-TATCAGC
C_parvum_AB271070         ---------- ---------- ---------- ---------- ---------- ----------


                          ....|....| ....|....| ....|....| ....|....| ....|....| ....|....|
                                 130        140        150        160        170        180
25330-C.ubiquitum         TTTAGACGGT AGGGTATTGG CCTACCGTGG CAA-TGACGG GTAACGGGGA A-TTAGGGTT
C_ubiquitum_KC608030      TTTAGACGGT AGGGTATTGG CCTACCGTGG CAA-TGACGG GTAACGGGGA A-TTAGGGTT
C_ubiquitum_KC962124      TTTAGACGGT AGGGTATTGG CCTACCGTGG CAA-TGACGG GTAACGGGGA A-TTAGGGTT
C_ubiquitum_AB697056      TTTAGACGGT AGGGTATTGG CCTACCGTGG CAA-TGACGG GTAACGGGGA A-TTAGGGTT
C_ubiquitum_KU531665      ---AGACGGT AGGGTATTGG CCTACCGTGG CAA-TGACGG GTAACGGGGA A-TTAGGGTT
C_felis_JQ312664          TTTATACGGA AGGGTATTGG CCTACCGTGG CTA-TGACGG GTAACGGGGA A-TTAGGGTT
C_felis_AJ493211          TTTAGACGGT AGGGTATTGG CCTACCGTGG CTA-TGACGG GTAACGGGGA A-TTAGGGTT
C_felis_JN833576          TTTAGACGGT AGGGTATTGG CCTACCGTGG CTA-TGACGG GTAACGGGGA A-TTAGGGTT
C_felis_AF159113          TTTAGACGGT AGGGTATTGG CCTACCGTGG CTA-TGACGG GTAACGGGGA A-TTAGGGTT
C_felis_KT749819          TTTAGACGGT AGGGTATTGG CCTACCGTGG CTA-TGACGG GTAACGGGGA A-TTAGGGTT
C_felis_FJ707310          TTTAGACGGT AGGGTATTGG CCTACCGTGG CTA-TGACGG GTAACGGGGA A-TTAGGGTT
C_felis_KM977642          TTTAGACGGT AGGGTATTGG CCTACCGTGG CTA-TGACGG GTAACGGGGA A-TTAGGGTT
C_muris_GU319781          TTTAGACGGT AGGGTATTGG CCTACCGTGG CTA-TGACGG GTAACGGGGA A-TTAGGGTT
C_muris_KF419208          TTTAGACGGT AGGGTATTGG CCTACCGTGG CTA-TGACGG GTAACGGGGA A-TTAGGGTT
C_muris_EU553592          TTTAGACGGT AGGGTATTGG CCTACCGTGG CTA-TGACGG GTAACGGGGA A-TTAGGGTT
C_muris_GU319783          TTTAGACGGT AGGGTATTGG CCTACCGTGG CTA-TGACGG GTAACGGGGA A-TTAGGGTT
C_muris_EU245045          TTTAGACGGT AGGGTATTGG CCTACCGTGG CTA-TGACGG GTAACGGGGA A-TTAGGGTT
C_muris_GQ227706          TTTAGACGGT AGGGTATTGG CCTACCGTGG CTA-TGACGG GTAACGGGGA A-TTAGGGTT
C_muris_EU156446          TTTAGACGGT AGGGTATTGG CCTACCGTGG CTA-TGACGG GTAACGGGGA A-TTAGGGTT
C_muris_AY642591          TTTAGACGGT AGGGTATTGG CCTACCGTGG CTA-TGACGG GTAACGGGGA A-TTAGGGTT
C_muris_KY490555          TTTAGACGGT AGGGTATTGG CCTACCGTGG CTA-TGACGG GTAACGGGGA A-TTAGGGTT
23767-C.muris             TTTAGACGGT AGGGTATTGG CCTACCGTGG CTA-TGACGG GTAACGGGGA A-TTAGGGTT
C_andersoni_AB449819      TTTAGACGGT AGGGTATTGG CCTACCGTGG CTA-TGACGG GTAACGGGGA A-TTAGGGTT
C_andersoni_EF613341      ---------- ---------- ---------- ---------- ---------- ----------
C_andersoni_KF826306      TTTAGACGGT AGGGTATTGG CCTACCGTGG CTA-TGACGG GTAACGGGGA A-TTAGGGTT
C_andersoni_KT922229      TTTAGACGGT AGGGTATTGG CCTACCGTGG CTA-TGACGG GTAACGGGGA A-TTAGGGTT
C_andersoni_KT884487      TTTAGACGGT AGGGTATTGG CCTACCGTGG CTA-TGACGG GTAACGGGGA A-TTAGGGTT
C_andersoni_LC012014      TTTAGACGGT AGGGTATTGG CCTACCGTGG CTA-TGACGG GTAACGGGGA A-TTAGGGTT
C_andersoni_AB449816      TTTAGACGGT AGGGTATTGG CCTACCGTGG CTA-TGACGG GTAACGGGGA A-TTAGGGTT
C_andersoni_AY954886      TTTAGACGGT AGGGTATTGG CCTACCGTGG CTA-TGACGG GTAACGGGGA A-TTAGGGTT
C_andersoni_AY954885      TTTAGACGGT AGGGTATTGG CCTACCGTGG CTA-TGACGG GTAACGGGGA A-TTAGGGTT
C_andersoni_AB089285      TTTAGACGGT AGGGTATTGG CCTACCGTGG CTA-TGACGG GTAACGGGGA A-TTAGGGTT
C_andersoni_LC012013      TTTAGACGGT AGGGTATTGG CCTACCGTGG CTA-TGACGG GTAACGGGGA A-TTAGGGTT
C_andersoni_KF826307      TTTAGACGGT AGGGTATTGG CCTACCGTGG CTA-TGACGG GTAACGGGGA A-TTAGGGTT
C_andersoni_KF826305      TTTAGACGGT AGGGTATTGG CCTACCGTGG CTA-TGACGG GTAACGGGGA A-TTAGGGTT
C_andersoni_KF826304      TTTAGACGGT AGGGTATTGG CCTACCGTGG CTA-TGACGG GTAACGGGGA A-TTAGGGTT
```

```
C_andersoni_EU245042   TTTAGACGGT AGGGTATTGG CCTACCGTGG CTA-TGACGG GTAACGGGGA A-TTAGGGTT
C_andersoni_KT922228   TTTAGACGGT AGGGTATTGG CCTACCGTGG CTA-TGACGG GTAACGGGGA A-TTAGGGTT
C_andersoni_KT884488   TTTAGACGGT AGGGTATTGG CCTACCGTGG CTA-TGACGG GTAACGGGGA A-TTAGGGTT
C_andersoni_AB449817   TTTAGACGGT AGGGTATTGG CCTACCGTGG CTA-TGACGG GTAACGGGGA A-TTAGGGTT
C_andersoni_KT922230   TTTAGACGGT AGGGTATTGG CCTACCGTGG CTA-TGACGG GTAACGGGGA A-TTAGGGTT
C_hominis_KF826315     TTTAGACGGT AGGGTATTGG CCTACCGTGG CAA-TGACGG GTAACGGGGA A-TTAGGGTT
C_hominis_HQ149022     TTTAGACGGT AGGGTATTGG CCTACCGTGG CAA-TGACGG GTAACGGGGA A-TTAGGGTT
C_hominis_KR296813     TTTAGACGGT AGGGTATTGG CCTACCGTGG CAA-TGACGG GTAACGGGGA A-TTAGGGTT
C_hominis_AF112569     TTTAGACGGT AGGGTATTGG CCTACCGTGG CAA-TGACGG GTAACGGGGA A-TTAGGGTT
C_hominis_KF679723     TTTAGACGGT AGGGTATTGG CCTACCGTGG CAA-TGACGG GTAACGGGGA A-TTAGGGTT
24937-C.hominis        TTTAGACGGT AGGGTATTGG CCTACCGTGG CAA-TGACGG GTAACGGGGA A-TTAGGGTT
C_parvum_KP204486      TTTAGACGGT AGGGTATTGG CCTACCGTGG CAA-TGACGG GTAACGGGGA A-TTAGGGTT
C_parvum_KM012042      TTTAGACGGT AGGGTATTGG CCTACCGTGG CAA-TGACGG GTAACGGGGA A-TTAGGGTT
C_parvum_AY268582      TTTAGACGGT AGGGTATTGG CCTACCGTGG CAA-TGACGG GTAACGGGGA A-TTAGGGTT
C_parvum_KM012045      TTTAGACGGT AGGGTATTGG CCTACCGTGG CAA-TGACGG GTAACGGGGA A-TTAGGGTT
C_parvum_KM012043      TTTAGACGGT AGGGTATTGG CCTACCGTGG CAA-TGACGG GTAACGGGGA A-TTAGGGTT
C_parvum_AF108864      TTTAGACGGT AGGGTATTGG CCTACCGTGG CAA-TGACGG GTAACGGGGA A-TTAGGGTT
C_parvum_KM085018      TTTAGACGGT AGGGTATTGG CCTACCGTGG CAA-TGACGG GTAACGGGGA A-TTAGGGTT
C_parvum_LC01201       TTTAGACGGT AGGGTATTGG CCTACCGTGG CAA-TGACGG GTAACGGGGA A-TTAGGGTT
C_parvum_KJ808688      TTTAGACGGT AGGGTATTGG CCTACCGTGG CAA-TGACGG GTAACGGGGA A-TTAGGGTT
C_parvum_LC012016      TTTAGACGGT AGGGTATTGG CCTACCGTGG CAA-TGACGG GTAACGGGGA A-TTAGGGTT
C_parvum_KM012040      TTTAGACGGT AGGGTATTGG CCTACCGTGG CAA-TGACGG GTAACGGGGA A-TTAGGGTT
C_parvum_KJ808687      TTTAGACGGT AGGGTATTGG CCTACCGTGG CAA-TGACGG GTAACGGGGA A-TTAGGGTT
C_parvum_KM012044      TTTAGACGGT AGGGTATTGG CCTACCGTGG CAA-TGACGG GTAACGGGGA A-TTAGGGTT
C_parvum_KP334136      TTTAGACGGT AGGGTATTGG CCTACCGTGG CAA-TGACGG GTAACGGGGA A-TTAGGGTT
C_parvum_KJ808689      TTTAGACGGT AGGGTATTGG CCTACCGTGG CAA-TGACGG GTAACGGGGA A-TTAGGGTT
C_parvum_KM012046      TTTAGACGGT AGGGTATTGG CCTACCGTGG CAA-TGACGG GTAACGGGGA A-TTAGGGTT
C_parvum_LC012015      TTTAGACGGT AGGGTATTGG CCTACCGTGG CAA-TGACGG GTAACGGGGA A-TTAGGGTT
C_parvum_K16X154       TTTAGACGGT AGGGTATTGG CCTACCGTGG CAA-TGACGG GTAACGGGGA A-TTAGGGTT
C_parvum_KJ808691      TTTAGACGGT AGGGTATTGG CCTACCGTGG CAA-TGACGG GTAACGGGGA A-TTAGGGTT
C_parvum_KU892559      TTTAGACGGT AGGGTATTGG CCTACCGTGG CAA-TGACGG GTAACGGGGA A-TTAGGGTT
C_parvum_AB968048      TTTAGACGGT AGGGTATTGG CCTACCGTGG CAA-TGACGG GTAACGGGGA A-TTAGGGTT
C_parvum_KJ808690      TTTAGACGGT AGGGTATTGG CCTACCGTGG CAA-TGACGG GTAACGGGGA A-TTAGGGTT
C_parvum_KJ808692      TTTAGACGGT AGGGTATTGG CCTACCGTGG CAA-TGACGG GTAACGGGGA A-TTAGGGTT
C_parvum_AF093493      TTTAGACGGT AGGGTATTGG CCTACCGTGG CAA-TGACGG GTAACGGGGA A-TTAGGGTT
C_parvum_EU553550      TTTAGACGGT AGGGTATTGG CCTACCGTGG CAA-TGACGG GTAACGGGGA A-TTAGGGTT
C_parvum_EU553557      TTTAGACGGT AGGGTATTGG CCTACCGTGG CAA-TGACGG GTAACGGGGA A-TTAGGGTT
C_parvum_KJ808693      TTTAGACGGT AGGGTATTGG CCTACCGTGG CAA-TGACGG GTAACGGGGA A-TTAGGGTT
C_parvum_KJ808694      TTTAGACGGT AGGGTATTGG CCTACCGTGG CAA-TGACGG GTAACGGGGA A-TTAGGGTT
C_parvum_KJ808695      TTTAGACGGT AGGGTATTGG CCTACCGTGG CAA-TGACGG GTAACGGGGA A-TTAGGGTT
25115-C.parvum         TTTAGGCGGT AGGGTATTGG CCTACCGTGG CAA-TGACGG GTAACGGGGA A-TTAGGGTT
C_parvum_KP004204      TTTAGACGGT AGGGTATTGG CCTACCGTGG CAA-TGACGG GTAACGGGGA A-TTAGGGTT
C_parvum_KP004203      TTTAGACGGT AGGGTATTGG CCTACCGTGG CAA-TGACGG GTAACGGGGA A-TTAGGGTT
C_parvum_AB271070      ---------- ---------- ---------- ---------- ---------- ----------


                       ....|....| ....|....| ....|....| ....|....| ....|....| ....|....|
                                190        200        210        220        230        240
25330-C.ubiquitum      CGATTCC-GG AGAGGGAGCC --TGAGAAAC ----GGCTAC CACATCT-AA GGAAGGCAGC
C_ubiquitum_KC608030   CGATTCC-GG AGAGGGAGCC --TGAGAAAC ----GGCTAC CACATCT-AA GGAAGGCAGC
C_ubiquitum_KC962124   CGATTCC-GG AGAGGGAGCC --TGAGAAAC ----GGCTAC CACATCT-AA GGAAGGCAGC
C_ubiquitum_AB697056   CGATTCC-GG AGAGGGAGCC --TGAGAAAC ----GGCTAC CACATCT-AA GGAAGGCAGC
C_ubiquitum_KU531665   CGATTCC-GG AGAGGGAGCC --TGAGAAAC ----GGCTAC CACATCT-AA GGAAGGCAGC
C_felis_JQ312664       CGATTCC-GG AGAGGGGGCC --TGACAAAC ----GGCTAC CACCTCT-AT GGAAGGCAGC
C_felis_AJ493211       CGATTCC-GG AGAGGGAGCC --TGAGAAAC ----GGCTAC CACATCT-AA GGAAGGCAGC
C_felis_JN833576       CGATTCC-GG AGAGGGAGCC --TGAGAAAC ----GGCTAC CACATCT-AA GGAAGGCAGC
C_felis_AF159113       CGATTCC-GG AGAGGGAGCC --TGAGAAAC ----GGCTAC CACATCT-AA GGAAGGCAGC
C_felis_KT749819       CGATTCC-GG AGAGGGAGCC --TGAGAAAC ----GGCTAC CACATCT-AA GGAAGGCAGC
C_felis_FJ707310       CGATTCC-GG AGAGGGAGCC --TGAGAAAC ----GGCTAC CACATCT-AA GGAAGGCAGC
C_felis_KM977642       CGATTCC-GG AGAGGGAGCC --TGAGAAAC ----GGCTAC CACATCT-AA GGAAGGCAGC
C_muris_GU319781       CGATTCC-GG AGAGGGAGCC --TGAGAAAC ----GGCTAC CACATCT-AA GGAAGGCAGC
C_muris_KF419208       CGATTCC-GG AGAGGGAGCC --TGAGAAAC ----GGCTAC CACATCT-AA GGAAGGCAGC
C_muris_EU553592       CGATTCC-GG AGAGGGAGCC --TGAGAAAC ----GGCTAC CACATCT-AA GGAAGGCAGC
C_muris_GU319783       CGATTCC-GG AGAGGGAGCC --TGAGAAAC ----GGCTAC CACATCT-AA GGAAGGCAGC
C_muris_EU245045       CGATTCC-GG AGAGGGAGCC --TGAGAAAC ----GGCTAC CACATCT-AA GGAAGGCAGC
C_muris_GQ227706       CGATTCC-GG AGAGGGAGCC --TGAGAAAC ----GGCTAC CACATCT-AA GGAAGGCAGC
C_muris_EU156446       CGATTCC-GG AGAGGGAGCC --TGAGAAAC ----GGCTAC CACATCT-AA GGAAGGCAGC
C_muris_AY642591       CGATTCC-GG AGAGGGAGCC --TGAGAAAC ----GGCTAC CACATCT-AA GGAAGGCAGC
C_muris_KY490555       CGATTCC-GG AGAGGGAGCC --TGAGAAAC ----GGCTAC CACATCT-AA GGAAGGCAGC
23767-C.muris          CGATTCC-GG AGAGGGAGCC --TGAGAAAC ----GGCTAC CACATCT-AA GGAAGGCAGC
C_andersoni_AB449819   CGATTCC-GG AGAGGGAGCC --TGAGAAAC ----GGCTAC CACATCT-AA GGAAGGCAGC
```

```
C_andersoni_EF613341      ---------- ---------- ---------- ---------- ---------- ----------
C_andersoni_KF826306      CGATTCC-GG AGAGGGAGCC --TGAGAAAC ----GGCTAC CACATCT-AA GGAAGGCAGC
C_andersoni_KT922229      CGATTCC-GG AGAGGGAGCC --TGAGAAAC ----GGCTAC CACATCT-AA GGAAGGCAGC
C_andersoni_KT884487      CGATTCC-GG AGAGGGAGCC --TGAGAAAC ----GGCTAC CACATCT-AA GGAAGGCAGC
C_andersoni_LC012014      CGATTCC-GG AGAGGGAGCC --TGAGAAAC ----GGCTAC CACATCT-AA GGAAGGCAGC
C_andersoni_AB449816      CGATTCC-GG AGAGGGAGCC --TGAGAAAC ----GGCTAC CACATCT-AA GGAAGGCAGC
C_andersoni_AY954886      CGATTCC-GG AGAGGGAGCC --TGAGAAAC ----GGCTAC CACATCT-AA GGAAGGCAGC
C_andersoni_AY954885      CGATTCC-GG AGAGGGAGCC --TGAGAAAC ----GGCTAC CACATCT-AA GGAAGGCAGC
C_andersoni_AB089285      CGATTCC-GG AGAGGGAGCC --TGAGAAAC ----GGCTAC CACATCT-AA GGAAGGCAGC
C_andersoni_LC012013      CGATTCC-GG AGAGGGAGCC --TGAGAAAC ----GGCTAC CACATCT-AA GGAAGGCAGC
C_andersoni_KF826307      CGATTCC-GG AGAGGGAGCC --TGAGAAAC ----GGCTAC CACATCT-AA GGAAGGCAGC
C_andersoni_KF826305      CGATTCC-GG AGAGGGAGCC --TGAGAAAC ----GGCTAC CACATCT-AA GGAAGGCAGC
C_andersoni_KF826304      CGATTCC-GG AGAGGGAGCC --TGAGAAAC ----GGCTAC CACATCT-AA GGAAGGCAGC
C_andersoni_EU245042      CGATTCC-GG AGAGGGAGCC --TGAGAAAC ----GGCTAC CACATCT-AA GGAAGGCAGC
C_andersoni_KT922228      CGATTCC-GG AGAGGGAGCC --TGAGAAAC ----GGCTAC CACATCT-AA GGAAGGCAGC
C_andersoni_KT884488      CGATTCC-GG AGAGGGAGCC --TGAGAAAC ----GGCTAC CACATCT-AA GGAAGGCAGC
C_andersoni_AB449817      CGATTCC-GG AGAGGGAGCC --TGAGAAAC ----GGCTAC CACATCT-AA GGAAGGCAGC
C_andersoni_KT922230      CGATTCC-GG AGAGGGAGCC --TGAGAAAC ----GGCTAC CACATCT-AA GGAAGGCAGC
C_hominis_KF826315        CGATTCC-GG AGAGGGAGCC --TGAGAAAC ----GGCTAC CACATCT-AA GGAAGGCAGC
C_hominis_HQ149022        CGATTCC-GG AGAGGGAGCC --TGAGAAAC ----GGCTAC CACATCT-AA GGAAGGCAGC
C_hominis_KR296813        CGATTCC-GG AGAGGGAGCC --TGAGAAAC ----GGCTAC CACATCT-AA GGAAGGCAGC
C_hominis_AF112569        CGATTCC-GG AGAGGGAGCC --TGAGAAAC ----GGCTAC CACATCT-AA GGAAGGCAGC
C_hominis_KF679723        CGATTCC-GG AGAGGGAGCC --TGAGAAAC ----GGCTAC CACATCT-AA GGAAGGCAGC
24937-C.hominis           CGATTCC-GG AGAGGGAGCC --TGAGAAAC ----GGCTAC CACATCT-AA GGAAGGCAGC
C_parvum_KP204486         CGATTCC-GG AGAGGGAGCC --TGAGAAAC ----GGCTAC CACATCT-AA GGAAGGCAGC
C_parvum_KM012042         CGATTCC-GG AGAGGGAGCC --TGAGAAAC ----GGCTAC CACATCT-AA GGAAGGCAGC
C_parvum_AY268582         CGATTCC-GG AGAGGGAGCC --TGAGAAAC ----GGCTAC CACATCT-AA GGAAGGCAGC
C_parvum_KM012045         CGATTCC-GG AGAGGGAGCC --TGAGAAAC ----GGCTAC CACATCT-AA GGAAGGCAGC
C_parvum_KM012043         CGATTCC-GG AGAGGGAGCC --TGAGAAAC ----GGCTAC CACATCT-AA GGAAGGCAGC
C_parvum_AF108864         CGATTCC-GG AGAGGGAGCC --TGAGAAAC ----GGCTAC CACATCT-AA GGAAGGCAGC
C_parvum_KM085018         CGATTCC-GG AGAGGGAGCC --TGAGAAAC ----GGCTAC CACATCT-AA GGAAGGCAGC
C_parvum_LC01201          CGATTCC-GG AGAGGGAGCC --TGAGAAAC ----GGCTAC CACATCT-AA GGAAGGCAGC
C_parvum_KJ808688         CGATTCC-GG AGAGGGAGCC --TGAGAAAC ----GGCTAC CACATCT-AA GGAAGGCAGC
C_parvum_LC012016         CGATTCC-GG AGAGGGAGCC --TGAGAAAC ----GGCTAC CACATCT-AA GGAAGGCAGC
C_parvum_KM012040         CGATTCC-GG AGAGGGAGCC --TGAGAAAC ----GGCTAC CACATCT-AA GGAAGGCAGC
C_parvum_KJ808687         CGATTCC-GG AGAGGGAGCC --TGAGAAAC ----GGCTAC CACATCT-AA GGAAGGCAGC
C_parvum_KM012044         CGATTCC-GG AGAGGGAGCC --TGAGAAAC ----GGCTAC CACATCT-AA GGAAGGCAGC
C_parvum_KP334136         CGATTCC-GG AGAGGGAGCC --TGAGAAAC ----GGCTAC CACATCT-AA GGAAGGCAGC
C_parvum_KJ808689         CGATTCC-GG AGAGGGAGCC --TGAGAAAC ----GGCTAC CACATCT-AA GGAAGGCAGC
C_parvum_KM012046         CGATTCC-GG AGAGGGAGCC --TGAGAAAC ----GGCTAC CACATCT-AA GGAAGGCAGC
C_parvum_LC012015         CGATTCC-GG AGAGGGAGCC --TGAGAAAC ----GGCTAC CACATCT-AA GGAAGGCAGC
C_parvum_K16X154          CGATTCC-GG AGAGGGAGCC --TGAGAAAC ----GGCTAC CACATCT-AA GGAAGGCAGC
C_parvum_KJ808691         CGATTCC-GG AGAGGGAGCC --TGAGAAAC ----GGCTAC CACATCT-AA GGAAGGCAGC
C_parvum_KU892559         CGATTCC-GG AGAGGGAGCC --TGAGAAAC ----GGCTAC CACATCT-AA GGAAGGCAGC
C_parvum_AB968048         CGATTCC-GG AGAGGGAGCC --TGAGAAAC ----GGCTAC CACATCT-AA GGAAGGCAGC
C_parvum_KJ808690         CGATTCC-GG AGAGGGAGCC --TGAGAAAC ----GGCTAC CACATCT-AA GGAAGGCAGC
C_parvum_KJ808692         CGATTCC-GG AGAGGGAGCC --TGAGAAAC ----GGCTAC CACATCT-AA GGAAGGCAGC
C_parvum_AF093493         CGATTCC-GG AGAGGGAGCC --TGAGAAAC ----GGCTAC CACATCT-AA GGAAGGCAGC
C_parvum_EU553550         CGATTCC-GG AGAGGGAGCC --TGAGAAAC ----GGCTAC CACATCT-AA GGAAGGCAGC
C_parvum_EU553557         CGATTCC-GG AGAGGGAGCC --TGAGAAAC ----GGCTAC CACATCT-AA GGAAGGCAGC
C_parvum_KJ808693         CGATTCC-GG AGAGGGAGCC --TGAGAAAC ----GGCTAC CACATCT-AA GGAAGGCAGC
C_parvum_KJ808694         CGATTCC-GG AGAGGGAGCC --TGAGAAAC ----GGCTAC CACATCT-AA GGAAGGCAGC
C_parvum_KJ808695         CGATTCC-GG AGAGGGAGCC --TGAGAAAC ----GGCTAC CACATCT-AA GGAAGGCAGC
25115-C.parvum            CGATTCC-GG AGAGGGAGCC --TGAGAAGC ----GGCTAC CACATCT-AA GGAAGGCAGC
C_parvum_KP004204         CGATTCC-GG AGAGGGAGCC --TGAGAAAC ----GGCTAC CACATCT-AA GGAAGGCAGC
C_parvum_KP004203         CGATTCC-GG AGAGGGAGCC --TGAGAAAC ----GGCTAC CACATCT-AA GGAAGGCAGC
C_parvum_AB271070         ---------- ---------- ---------- ---------- ---------- ----------


                          ....|....| ....|....| ....|....| ....|....| ....|....| ....|....|
                                 250        260        270        280        290        300
25330-C.ubiquitum         AGGCGCGC-A AATTACCCAA TCC-TAATAC AGGGAGGTAG -TGACAAGAA ATAAC-AATA
C_ubiquitum_KC608030      AGGCGCGC-A AATTACCCAA TCC-TAATAC AGGGAGGTAG -TGACAAGAA ATAAC-AATA
C_ubiquitum_KC962124      AGGCGCGC-A AATTACCCAA TCC-TAATAC AGGGAGGTAG -TGACAAGAA ATAAC-AATA
C_ubiquitum_AB697056      AGGCGCGC-A AATTACCCAA TCC-TAATAC AGGGAGGTAG -TGACAAGAA ATAAC-AATA
C_ubiquitum_KU531665      AGGCGCGC-A AATTACCCAA TCC-TAATAC AGGGAGGTAG -TGACAAGAA ATAAC-AATA
C_felis_JQ312664          AGGCGCGC-A AATTACCCAA TCC-TAATAC AGGGAGGTAG -GGACAAGAA ATAAC-AATA
C_felis_AJ493211          AGGCGCGC-A AATTACCCAA TCC-TAATAC AGGGAGGTAG -TGACAAGAA ATAAC-AATA
C_felis_JN833576          AGGCGCGC-A AATTACCCAA TCC-TAATAC AGGGAGGTAG -TGACAAGAA ATAAC-AATA
C_felis_AF159113          AGGCGCGC-A AATTACCCAA TCC-TAATAC AGGGAGGTAG -TGACAAGAA ATAAC-AATA
C_felis_KT749819          CGGCGCGC-A AATTACCCAA TCC-TAATAC AGGGAGGTAG -TGACAAGAA ATAAC-AATA
```

```
C_felis_FJ707310        AGGCGCGC-A AATTACCCAA TCC-TAATAC AGGGAGGTAG -TGACAAGAA ATAAC-AATA
C_felis_KM977642        AGGCGCGC-A AATTACCCAA TCC-TAATAC AGGGAGGTAG -TGACAAGAA ATAAC-AATA
C_muris_GU319781        AGGCGCGC-A AATTACCCAA TCC-TGACAC AGGGAGGTAG -TGACAAGAA ATAAC-AATA
C_muris_KF419208        AGGCGCGC-A AATTACCCAA TCC-TGACAC AGGGAGGTAG -TGACAAGAA ATAAC-AATA
C_muris_EU553592        AGGCGCGC-A AATTACCCAA TCC-TGACAC AGGGAGGTAG -TGACAAGAA ATAAC-AATA
C_muris_GU319783        AGGCGCGC-A AATTACCCAA TCC-TGACAC AGGGAGGTAG -TGACAAGAA ATAAC-AATA
C_muris_EU245045        AGGCGCGC-A AATTACCCAA TCC-TGACAC AGGGAGGTAG -TGACAAGAA ATAAC-AATA
C_muris_GQ227706        AGGCGCGC-A AATTACCCAA TCC-TGACAC AGGGAGGTAG -TGACAAGAA ATAAC-AATA
C_muris_EU156446        AGGCGCGC-A AATTACCCAA TCC-TGACAC AGGGAGGTAG -TGACAAGAA ATAAC-AATA
C_muris_AY642591        AGGCGCGC-A AATTACCCAA TCC-TGACAC AGGGAGGTAG -TGACAAGAA ATAAC-AATA
C_muris_KY490555        AGGCGCGC-A AATTACCCAA TCC-TGACAC AGGGAGGTAG -TGACAAGAA ATAAC-AATA
23767-C.muris           AGGCGCGC-A AATTACCCAA TCC-TGACAC AGGGAGGTAG -TGACAAGAA ATAAC-AATA
C_andersoni_AB449819    AGGCGCGC-A AATTACCCAA TCC-TGACAC AGGGAGGTAG -TGACAAGAA ATAAC-AATA
C_andersoni_EF613341    ---------- ---------- ---------- ---------- ---------- ----------
C_andersoni_KF826306    AGGCGCGC-A AATTACCCAA TCC-TGACAC AGGGAGGTAG -TGACAAGAA ATAAC-AATA
C_andersoni_KT922229    AGGCGCGC-A AATTACCCAA TCC-TGACAC AGGGAGGTAG -TGACAAGAA ATAAC-AATA
C_andersoni_KT884487    AGGCGCGC-A AATTACCCAA TCC-TGACAC AGGGAGGTAG -TGACAAGAA ATAAC-AATA
C_andersoni_LC012014    AGGCGCGC-A AATTACCCAA TCC-TGACAC AGGGAGGTAG -TGACAAGAA ATAAC-AATA
C_andersoni_AB449816    AGGCGCGC-A AATTACCCAA TCC-TGACAC AGGGAGGTAG -TGACAAGAA ATAAC-AATA
C_andersoni_AY954886    AGGCGCGC-A AATTACCCAA TCC-TGACAC AGGGAGGTAG -TGACAAGAA ATAAC-AATA
C_andersoni_AY954885    AGGCGCGC-A AATTACCCAA TCC-TGACAC AGGGAGGTAG -TGACAAGAA ATAAC-AATA
C_andersoni_AB089285    AGGCGCGC-A AATTACCCAA TCC-TGACAC AGGGAGGTAG -TGACAAGAA ATAAC-AATA
C_andersoni_LC012013    AGGCGCGC-A AATTACCCAA TCC-TGACAC AGGGAGGTAG -TGACAAGAA ATAAC-AATA
C_andersoni_KF826307    AGGCGCGC-A AATTACCCAA TCC-TGACAC AGGGAGGTAG -TGACAAGAA ATAAC-AATA
C_andersoni_KF826305    AGGCGCGC-A AATTACCCAA TCC-TGACAC AGGGAGGTAG -TGACAAGAA ATAAC-AATA
C_andersoni_KF826304    AGGCGCGC-A AATTACCCAA TCC-TGACAC AGGGAGGTAG -TGACAAGAA ATAAC-AATA
C_andersoni_EU245042    AGGCGCGC-A AATTACCCAA TCC-TGACAC AGGGAGGTAG -TGACAAGAA ATAAC-AATA
C_andersoni_KT922228    AGGCGCGC-A AATTACCCAA TCC-TGACAC AGGGAGGTAG -TGACAAGAA ATAAC-AATA
C_andersoni_KT884488    AGGCGCGC-A AATTACCCAA TCC-TGACAC AGGGAGGTAG -TGACAAGAA ATAAC-AATA
C_andersoni_AB449817    AGGCGCGC-A AATTACCCAA TCC-TGACAC AGGGAGGTAG -TGACAAGAA ATAAC-AATA
C_andersoni_KT922230    AGGCGCGC-A AATTACCCAA TCC-TGACAC AGGGAGGTAG -TGACAAGAA ATAAC-AATA
C_hominis_KF826315      AGGCGCGC-A AATTACCCAA TCC-TAATAC AGGGAGGTAG -TGACAAGAA ATAAC-AATA
C_hominis_HQ149022      AGGCGCGC-A AATTACCCAA TCC-TAATAC AGGGAGGTAG -TGACAAGAA ATAAC-AATA
C_hominis_KR296813      AGGCGCGC-A AATTACCCAA TCC-TAATAC AGGGAGGTAG -TGACAAGAA ATAAC-AATA
C_hominis_AF112569      AGGCGCGC-A AATTACCCAA TCC-TAATAC AGGGAGGTAG -TGACAAGAA ATAAC-AATA
C_hominis_KF679723      AGGCGCGC-A AATTACCCAA TCC-TAATAC AGGGAGGTAG -TGACAAGAA ATAAC-AATA
24937-C.hominis         AGGCGCGC-A AATTACCCAA TCC-TAATAC AGGGAGGTAG -TGACAAGAA ATAAC-AATA
C_parvum_KP204486       AGGCGCGC-A AATTACCCAA TCC-TAATAC AGGGAGGTAG -TGACAAGAA ATAAC-AATA
C_parvum_KM012042       AGGCGCGC-A AATTACCCAA TCC-TAATAC AGGGAGGTAG -TGACAAGAA ATAAC-AATA
C_parvum_AY268582       AGGCGCGC-A AATTACCCAA TCC-TAATAC AGGGAGGTAG -TGACAAGAA ATAAC-AATA
C_parvum_KM012045       AGGCGCGC-A AATTACCCAA TCC-TAATAC AGGGAGGTAG -TGACAAGAA ATAAC-AATA
C_parvum_KM012043       AGGCGCGC-A AATTACCCAA TCC-TAATAC AGGGAGGTAG -TGACAAGAA ATAAC-AATA
C_parvum_AF108864       AGGCGCGC-A AATTACCCAA TCC-TAATAC AGGGAGGTAG -TGACAAGAA ATAAC-AATA
C_parvum_KM085018       AGGCGCGC-A AATTACCCAA TCC-TAATAC AGGGAGGTAG -TGACAAGAA ATAAC-AATA
C_parvum_LC01201        AGGCGCGC-A AATTACCCAA TCC-TAATAC AGGGAGGTAG -TGACAAGAA ATAAC-AATA
C_parvum_KJ808688       AGGCGCGC-A AATTACCCAA TCC-TAATAC AGGGAGGTAG -TGACAAGAA ATAAC-AATA
C_parvum_LC012016       AGGCGCGC-A AATTACCCAA TCC-TAATAC AGGGAGGTAG -TGACAAGAA ATAAC-AATA
C_parvum_KM012040       AGGCGCGC-A AATTACCCAA TCC-TAATAC AGGGAGGTAG -TGACAAGAA ATAAC-AATA
C_parvum_KJ808687       AGGCGCGC-A AATTACCCAA TCC-TAATAC AGGGAGGTAG -TGACAAGAA ATAAC-AATA
C_parvum_KM012044       AGGCGCGC-A AATTACCCAA TCC-TAATAC AGGGAGGTAG -TGACAAGAA ATAAC-AATA
C_parvum_KP334136       AGGCGCGC-A AATTACCCAA TCC-TAATAC AGGGAGGTAG -TGACAAGAA ATAAC-AATA
C_parvum_KJ808689       AGGCGCGC-A AATTACCCAA TCC-TAATAC AGGGAGGTAG -TGACAAGAA ATAAC-AATA
C_parvum_KM012046       AGGCGCGC-A AATTACCCAA TCC-TAATAC AGGGAGGTAG -TGACAAGAA ATAAC-AATA
C_parvum_LC012015       AGGCGCGC-A AATTACCCAA TCC-TAATAC AGGGAGGTAG -TGACAAGAA ATAAC-AATA
C_parvum_K16X154        AGGCGCGC-A AATTACCCAA TCC-TAATAC AGGGAGGTAG -TGACAAGAA ATAAC-AATA
C_parvum_KJ808691       AGGCGCGC-A AATTACCCAA TCC-TAATAC AGGGAGGTAG -TGACAAGAA ATAAC-AATA
C_parvum_KU892559       AGGCGCGC-A AATTACCCAA TCC-TAATAC AGGGAGGTAG -TGACAAGAA ATAAC-AATA
C_parvum_AB968048       AGGCGCGC-A AATTACCCAA TCC-TAATAC AGGGAGGTAG -TGACAAGAA ATAAC-AATA
C_parvum_KJ808690       AGGCGCGC-A AATTACCCAA TCC-TAATAC AGGGAGGTAG -TGACAAGAA ATAAC-AATA
C_parvum_KJ808692       AGGCGCGC-A AATTACCCAA TCC-TAATAC AGGGAGGTAG -TGACAAGAA ATAAC-AATA
C_parvum_AF093493       AGGCGCGC-A AATTACCCAA TCC-TAATAC AGGGAGGTAG -TGACAAGAA ATAAC-AATA
C_parvum_EU553550       AGGCGCGC-A AATTACCCAA TCC-TAATAC AGGGAGGTAG -TGACAAGAA ATAAC-AATA
C_parvum_EU553557       AGGCGCGC-A AATTACCCAA TCC-TAATAC AGGGAGGTAG -TGACAAGAA ATAAC-AATA
C_parvum_KJ808693       AGGCGCGC-A AATTACCCAA TCC-TAATAC AGGGAGGTAG -TGACAAGAA ATAAC-AATA
C_parvum_KJ808694       AGGCGCGC-A AATTACCCAA TCC-TAATAC AGGGAGGTAG -TGACAAGAA ATAAC-AATA
C_parvum_KJ808695       AGGCGCGC-A AATTACCCAA TCC-TAATAC AGGGAGGTAG -TGACAAGAA ATAAC-AATA
25115-C.parvum          AGGCGCGC-A GATTACCCAA TCC-TAATAC AGGGAGGTAG -TGACAAGAA ATAAC-AATA
C_parvum_KP004204       AGGCGCGC-A AATTACCCAA TCC-TAATAC AGGGAGGTAG -TGACAAGAA ATAAC-AATA
C_parvum_KP004203       AGGCGCGC-A AATTACCCAA TCC-TAATAC AGGGAGGTAG -TGACAAGAA ATAAC-AATA
C_parvum_AB271070       ---------- ---------- ---------- ---------- ---------- ----------
```

```
                          ....|....| ....|....| ....|....| ....|....| ....|....| ....|....|
                                 310        320        330        340        350        360
25330-C.ubiquitum         CAGGACTTTA AATAGTTTTG TAAT--TGGA ATGAGTTAAG TATAAACCC- CTTTACAAGT
C_ubiquitum_KC608030      CAGGACTTTA AATAGTTTTG TAAT--TGGA ATGAGTTGAG TATAAACCC- CTTTACAAGT
C_ubiquitum_KC962124      CAGGACTTTA AATAGTTTTG TAAT--TGGA ATGAGTTAAG TATAAACCC- CTTTACAAGT
C_ubiquitum_AB697056      CAGGACTTTA AATAGTTTTG TAAT--TGGA ATGAGTTAAG TATAAACCC- CTTTACAAGT
C_ubiquitum_KU531665      CAGGACTTTA AATAGTTTTG TAAT--TGGA ATGAGTTAAG TATAAACCC- CTTTACAAGT
C_felis_JQ312664          CAGGACTT-- TACGGTTTTG TAAT--TGGA ATGAGTTAGT TATAAACCC- CTTTCCCAGT
C_felis_AJ493211          CAGGACTT-- TACGGTTTTG TAAT--TGGA ATGAGTTAAG TATAAACCC- CTTTACAAGT
C_felis_JN833576          CAGGACTT-- TACGGTTTTG TAAT--TGGA ATGAGTTAAG TATAAACCC- CTTTACAAGT
C_felis_AF159113          CAGGACTT-- TACGGTTTTG TAAT--TGGA ATGAGTTAAG TATAAACCC- CTTTACAAGT
C_felis_KT749819          CAGGACTT-- TCCGGTTTTG TAAT--TGGA ATGAGTTAAG TATAAACCC- CTTTACAAGT
C_felis_FJ707310          CAGGACTT-- TACGGTTTTG TAAT--TGGA ATGAGTTAAG TATAAACCC- CTTTACAAGT
C_felis_KM977642          CAGGACTT-- TACGGTTTTG TAAT--TGGA ATGAGTTAAG TATAAACCC- CTTTACAAGT
C_muris_GU319781          CAGGGCCT-- AACGGTCTTG TAAT--TGGA ATGAGTGAAG TATAAACCC- CTTTACGAGT
C_muris_KF419208          CAGGGCCT-- AACGGTCTTG TAAT--TGGA ATGAGTGAAG TATAAACCC- CTTTACGAGT
C_muris_EU553592          CAGGGCCT-- AACGGTCTTG TAAT--TGGA ATGAGTGAAG TATAAACCC- CTTTACGAGT
C_muris_GU319783          CAGGGCCT-- AACGGTCTTG TAAT--TGGA ATGAGTGAAG TATAAACCC- CTTTACGAGT
C_muris_EU245045          CAGGGCCT-- AACGGTCTTG TAAT--TGGA ATGAGTGAAG TATAAACCC- CTTTACGAGT
C_muris_GQ227706          CAGGGCCT-- AACGGTCTTG TAAT--TGGA ATGAGTGAAG TATAAACCC- CTTTACGAGT
C_muris_EU156446          CAGGGCCT-- AACGGTCTTG TAAT--TGGA ATGAGTGAAG TATAAACCC- CTTTACGAGT
C_muris_AY642591          CAGGGCCT-- AACGGTCTTG TAAT--TGGA ATGAGTGAAG TATAAACCC- CTTTACGAGT
C_muris_KY490555          CAGGGCCT-- AACGGTCTTG TAAT--TGGA ATGAGTGAAG TATAAACCC- CTTTACGAGT
23767-C.muris             CAGGGCCT-- AACGGTCTTG TAAT--TGGA ATGAGTGAAG TATAAACCC- CTTTACGAGT
C_andersoni_AB449819      CAGGGCCT-- AACGGTCTTG TAAT--TGGA ATGAGTGAAG TATAAACCC- CTTTACGAGT
C_andersoni_EF613341      ---------- ---------- ---------- ---------- ---------- ----------
C_andersoni_KF826306      CAGGGCCT-- AACGGTCTTG TAAT--TGGA ATGAGTGAAG TATAAACCC- CTTTACGAGT
C_andersoni_KT922229      CAGGGCCT-- AACGGTCTTG TAAT--TGGA ATGAGTGAAG TATAAACCC- CTTTACGAGT
C_andersoni_KT884487      CAGGGCCT-- AACGGTCTTG TAAT--TGGA ATGAGTGAAG TATAAACCC- CTTTACGAGT
C_andersoni_LC012014      CAGGGCCT-- AACGGTCTTG TAAT--TGGA ATGAGTGAAG TATAAACCC- CTTTACGAGT
C_andersoni_AB449816      CAGGGCCT-- AACGGTCTTG TAAT--TGGA ATGAGTGAAG TATAAACCC- CTTTACGAGT
C_andersoni_AY954886      CAGGGCCT-- AACGGTCTTG TAAT--TGGA ATGAGTGAAG TATAAACCC- CTTTACGAGT
C_andersoni_AY954885      CAGGGCCT-- AACGGTCTTG TAAT--TGGA ATGAGTGAAG TATAAACCC- CTTTACGAGT
C_andersoni_AB089285      CAGGGCCT-- AACGGTCTTG TAAT--TGGA ATGAGTGAAG TATAAACCC- CTTTACGAGT
C_andersoni_LC012013      CAGGGCCT-- AACGGTCTTG TAAT--TGGA ATGAGTGAAG TATAAACCC- CTTTACGAGT
C_andersoni_KF826307      CAGGGCCT-- AACGGTCTTG TAAT--TGGA ATGAGTGAAG TATAAACCC- CTTTACGAGT
C_andersoni_KF826305      CAGGGCCT-- AACGGTCTTG TAAT--TGGA ATGAGTGAAG TATAAACCC- CTTTACGAGT
C_andersoni_KF826304      CAGGGCCT-- AACGGTCTTG TAAT--TGGA ATGAGTGAAG TATAAACCC- CTTTACGAGT
C_andersoni_EU245042      CAGGGCCT-- AACGGTCTTG TAAT--TGGA ATGAGTGAAG TATAAACCC- CTTTACGAGT
C_andersoni_KT922228      CAGGGCCT-- AACGGTCTTG TAAT--TGGA ATGAGTGAAG TATAAACCC- CTTTACGAGT
C_andersoni_KT884488      CAGGGCCT-- AACGGTCTTG TAAT--TGGA ATGAGTGAAG TATAAACCC- CTTTACGAGT
C_andersoni_AB449817      CAGGGCCT-- AACGGTCTTG TAAT--TGGA ATGAGTGAAG TATAAACCC- CTTTACGAGT
C_andersoni_KT922230      CAGGGCCT-- AACGGTCTTG TAAT--TGGA ATGAGTGAAG TATAAACCC- CTTTACGAGT
C_hominis_KF826315        CAGGACTT-- TTTGGTTTTG TAAT--TGGA ATGAGTTAAG TATAAACCC- CTTTACAAGT
C_hominis_HQ149022        CAGGACTT-- TTTGGTTTTG TAAT--TGGA ATGAGTTAAG TATAAACCC- CTTTACAAGT
C_hominis_KR296813        CAGGACTT-- TTTGGTTTTG TAAT--TGGA ATGAGTTAAG TATAAACCC- CTTTACAAGT
C_hominis_AF112569        CAGGACTT-- TTTGGTTTTG TAAT--TGGA ATGAGTTAAG TATAAACCC- CTTTACAAGT
C_hominis_KF679723        CAGGACTT-- TTTGGTTTTG TAAT--TGGA ATGAGTTAAG TATAAACCC- CTTTACAAGT
24937-C.hominis           CAGGACTT-- TTTGGTTTTG TAAT--TGGA ATGAGTTAAG TATAAACCC- CTTTACAAGT
C_parvum_KP204486         CAGGACTT-- TTTGGTTTTG TAAT--TGGA ATGAGTTAAG TATAAACCC- CTTTACAAGT
C_parvum_KM012042         CAGGACTT-- TTTGGTTTTG TAAT--TGGA ATGAGTTAAG TATAAACCC- CTTTACAAGT
C_parvum_AY268582         CAGGACTT-- TTTGGTTTTG TAAT--TGGA ATGAGTTAAG TATAAACCC- CTTTACAAGT
C_parvum_KM012045         CAGGACTT-- TTTGGTTTTG TAAT--TGGA ATGAGTTAAG TATAAACCC- CTTTACAAGT
C_parvum_KM012043         CAGGACTT-- TTTGGTTTTG TAAT--TGGA ATGAGTTAAG TATAAACCC- CTTTACAAGT
C_parvum_AF108864         CAGGACTT-- TTTGGTTTTG TAAT--TGGA ATGAGTTAAG TATAAACCC- CTTTACAAGT
C_parvum_KM085018         CAGGACTT-- TTTGGTTTTG TAAT--TGGA ATGAGTTAAG TATAAACCC- CTTTACAAGT
C_parvum_LC01201          CAGGACTT-- TTTGGTTTTG TAAT--TGGA ATGAGTTAAG TATAAACCC- CTTTACAAGT
C_parvum_KJ808688         CAGGACTT-- TTTGGTTTTG TAAT--TGGA ATGAGTTAAG TATAAACCC- CTTTACAAGT
C_parvum_LC012016         CAGGACTT-- TTTGGTTTTG TAAT--TGGA ATGAGTTAAG TATAAACCC- CTTTACAAGT
C_parvum_KM012040         CAGGACTT-- TTTGGTTTTG TAAT--TGGA ATGAGTTAAG TATAAACCC- CTTTACAAGT
C_parvum_KJ808687         CAGGACTT-- TTTGGTTTTG TAAT--TGGA ATGAGTTAAG TATAAACCC- CTTTACAAGT
C_parvum_KM012044         CAGGACTT-- TTTGGTTTTG TAAT--TGGA ATGAGTTAAG TATAAACCC- CTTTACAAGT
C_parvum_KP334136         CAGGACTT-- TTTGGTTTTG TAAT--TGGA ATGAGTTAAG TATAAACCC- CTTTACAAGT
C_parvum_KJ808689         CAGGACTT-- TTTGGTTTTG TAAT--TGGA ATGAGTTAAG TATAAACCC- CTTTACAAGT
C_parvum_KM012046         CAGGACTT-- TTTGGTTTTG TAAT--TGGA ATGAGTTAAG TATAAACCC- CTTTACAAGT
C_parvum_LC012015         CAGGACTT-- TTTGGTTTTG TAAT--TGGA ATGAGTTAAG TATAAACCC- CTTTACAAGT
C_parvum_K16X154          CAGGACTT-- TTTGGTTTTG TAAT--TGGA ATGAGTTAAG TATAAACCC- CTTTACAAGT
C_parvum_KJ808691         CAGGACTT-- TTTGGTTTTG TAAT--TGGA ATGAGTTAAG TATAAACCC- CTTTACAAGT
C_parvum_KU892559         CAGGACTT-- TTTGGTTTTG TAAT--TGGA ATGAGTTAAG TATAAACCC- CTTTACAAGT
C_parvum_AB968048         CAGGACTT-- TTTGGTTTTG TAAT--TGGA ATGAGTTAAG TATAAACCC- CTTTACAAGT
```

```
C_parvum_KJ808690        CAGGACTT-- TTTGGTTTTG TAAT--TGGA ATGAGTTAAG TATAAACCC- CTTTACAAGT
C_parvum_KJ808692        CAGGACTT-- TTTGGTTTTG TAAT--TGGA ATGAGTTAAG TATAAACCC- CTTTACAAGT
C_parvum_AF093493        CAGGACTT-- TTTGGTTTTG TAAT--TGGA ATGAGTTAAG TATAAACCC- CTTTACAAGT
C_parvum_EU553550        CAGGACTT-- TTTGGTTTTG TAAT--TGGA ATGAGTTAAG TATAAACCC- CTTTACAAGT
C_parvum_EU553557        CAGGACTT-- TTTGGTTTTG TAAT--TGGA ATGAGTTAAG TATAAACCC- CTTTACAAGT
C_parvum_KJ808693        CAGGACTT-- TTTGGTTTTG TAAT--TGGA ATGAGTTAAG TATAAACCC- CTTTACAAGT
C_parvum_KJ808694        CAGGACTT-- TTTGGTTTTG TAAT--TGGA ATGAGTTAAG TATAAACCC- CTTTACAAGT
C_parvum_KJ808695        CAGGACTT-- TTTGGTTTTG TAAT--TGGA ATGAGTTAAG TATAAACCC- CTTTACAAGT
25115-C.parvum           CAGGACTT-- TTTGGTTTTG TAAT--TGGA ATGAGTTAAG TATAAACCC- CTTTACAAGT
C_parvum_KP004204        CAGGACTT-- TTTGGTTTTG TAAT--TGGA ATGAGTTAAG TATAAACCC- CTTTACAAGT
C_parvum_KP004203        CAGGACTT-- TTTGGTTTTG TAAT--TGGA ATGAGTTAAG TATAAACCC- CTTTACAAGT
C_parvum_AB271070        ---------- ---------- ---------- ---------- ---------- ----------


                         ....|....| ....|....| ....|....| ....|....| ....|....| ....|....|
                                370        380        390        400        410        420
25330-C.ubiquitum        ATCAATTGGA GGGCAA-GTC TGGTGCCAGC -AGCC-GCGG TAATTCCAGC T-CCAATAGC
C_ubiquitum_KC608030     ATCAATTGGA GGGCAA-GTC TGGTGCCAGC -AGCC-GCGG TAATTCCAGC T-CCAATAGC
C_ubiquitum_KC962124     ATCAATTGGA GGGCAA-GTC TGGTGCCAGC -AGCC-GCGG TAATTCCAGC T-CCAATAGC
C_ubiquitum_AB697056     ATCAATTGGA GGGCAA-GTC TGGTGCCAGC -AGCC-GCGG TAATTCCAGC T-CCAATAGC
C_ubiquitum_KU531665     ATCAATTGGA GGGCAA-GTC TGGTGCCAGC -AGCC-GCGG TAATTCCAGC T-CCAATAGC
C_felis_JQ312664         ATCAATTGGA GGGCAA-GTC TGGTGCCAGC -ACCC-GCGG TAATTCCAGC T-CCAATAGC
C_felis_AJ493211         ATCAATTGGA GGGCAA-GTC TGGTGCCAGC -AGCC-GCGG TAATTCCAGC T-CCAATAGC
C_felis_JN833576         ATCAATTGGA GGGCAA-GTC TGGTGCCAGC -AGCC-GCGG TAATTCCAGC T-CCAATAGC
C_felis_AF159113         ATCAATTGGA GGGCAA-GTC TGGTGCCAGC -AGCC-GCGG TAATTCCAGC T-CCAATAGC
C_felis_KT749819         ATCAATTGGA GGGCAA-GTC TGGTGCCAGC -AGCC-GCGG TAATTCCAGC T-CCAATAGC
C_felis_FJ707310         ATCAATTGGA GGGCAA-GTC TGGTGCCAGC -AGCC-GCGG TAATTCCAGC T-CCAATAGC
C_felis_KM977642         ATCAATTGGA GGGCAA-GTC TGGTGCCAGC -AGCC-GCGG TAATTCCAGC T-CCAATAGC
C_muris_GU319781         ATCAATTGGA GGGCAA-GTC TGGTGCCAGC -AGCC-GCGG TAATTCCAGC T-CCAATAGC
C_muris_KF419208         ATCAATTGGA GGGCAA-GTC TGGTGCCAGC -AGCC-GCGG TAATTCCAGC T-CCAATAGC
C_muris_EU553592         ATCAATTGGA GGGCAA-GTC TGGTGCCAGC -AGCC-GCGG TAATTCCAGC T-CCAATAGC
C_muris_GU319783         ATCAATTGGA GGGCAA-GTC TGGTGCCAGC -AGCC-GCGG TAATTCCAGC T-CCAATAGC
C_muris_EU245045         ATCAATTGGA GGGCAA-GTC TGGTGCCAGC -AGCC-GCGG TAATTCCAGC T-CCAATAGC
C_muris_GQ227706         ATCAATTGGA GGGCAA-GTC TGGTGCCAGC -AGCC-GCGG TAATTCCAGC T-CCAATAGC
C_muris_EU156446         ATCAATTGGA GGGCAA-GTC TGGTGCCAGC -AGCC-GCGG TAATTCCAGC T-CCAATAGC
C_muris_AY642591         ATCAATTGGA GGGCAA-GTC TGGTGCCAGC -AGCC-GCGG TAATTCCAGC T-CCAATAGC
C_muris_KY490555         ATCAATTGGA GGGCAA-GTC TGGTGCCAGC -AGCC-GCGG TAATTCCAGC T-CCAATAGC
23767-C.muris            ATCAATTGGA GGGCAA-GTC TGGTGCCAGC -AGCC-GCGG TAATTCCAGC T-CCAATAGC
C_andersoni_AB449819     ATCAATTGGA GGGCAA-GTC TGGTGCCAGC -AGCC-GCGG TAATTCCAGC T-CCAATAGC
C_andersoni_EF613341     ---------- ---------- ---------- ---------- ---------- ----------
C_andersoni_KF826306     ATCAATTGGA GGGCAA-GTC TGGTGCCAGC -AGCC-GCGG TAATTCCAGC T-CCAATAGC
C_andersoni_KT922229     ATCAATTGGA GGGCAA-GTC TGGTGCCAGC -AGCC-GCGG TAATTCCAGC T-CCAATAGC
C_andersoni_KT884487     ATCAATTGGA GGGCAA-GTC TGGTGCCAGC -AGCC-GCGG TAATTCCAGC T-CCAATAGC
C_andersoni_LC012014     ATCAATTGGA GGGCAA-GTC TGGTGCCAGC -AGCC-GCGG TAATTCCAGC T-CCAATAGC
C_andersoni_AB449816     ATCAATTGGA GGGCAA-GTC TGGTGCCAGC -AGCC-GCGG TAATTCCAGC T-CCAATAGC
C_andersoni_AY954886     ATCAATTGGA GGGCAA-GTC TGGTGCCAGC -AGCC-GCGG TAATTCCAGC T-CCAATAGC
C_andersoni_AY954885     ATCAATTGGA GGGCAA-GTC TGGTGCCAGC -AGCT-GCGG TAATTCCAGC T-CCAATAGC
C_andersoni_AB089285     ATCAATTGGA GGGCAA-GTC TGGTGCCAGC -AGCC-GCGG TAATTCCAGC T-CCAATAGC
C_andersoni_LC012013     ATCAATTGGA GGGCAA-GTC TGGTGCCAGC -AGCC-GCGG TAATTCCAGC T-CCAATAGC
C_andersoni_KF826307     ATCAATTGGA GGGCAA-GTC TGGTGCCAGC -AGCC-GCGG TAATTCCAGC T-CCAATAGC
C_andersoni_KF826305     ATCAATTGGA GGGCAA-GTC TGGTGCCAGC -AGCC-GCGG TAATTCCAGC T-CCAATAGC
C_andersoni_KF826304     ATCAATTGGA GGGCAA-GTC TGGTGCCAGC -AGCC-GCGG TAATTCCAGC T-CCAATAGC
C_andersoni_EU245042     ATCAATTGGA GGGCAA-GTC TGGTGCCAGC -AGCC-GCGG TAATTCCAGC T-CCAATAGC
C_andersoni_KT922228     ATCAATTGGA GGGCAA-GTC TGGTGCCAGC -AGCC-GCGG TAATTCCAGC T-CCAATAGC
C_andersoni_KT884488     ATCAATTGGA GGGCAA-GTC TGGTGCCAGC -AGCC-GCGG TAATTCCAGC T-CCAATAGC
C_andersoni_AB449817     ATCAATTGGA GGGCAA-GTC TGGTGCCAGC -AGCC-GCGG TAATTCCAGC T-CCAATAGC
C_andersoni_KT922230     ATCAATTGGA GGGCAA-GTC TGGTGCCAGC -AGCC-GCGG TAATTCCAGC T-CCAATAGC
C_hominis_KF826315       ATCAATTGGA GGGCAA-GTC TGGTGCCAGC -AGCC-GCGG TAATTCCAGC T-CCAATAGC
C_hominis_HQ149022       ATCAATTGGA GGGCAA-GTC TGGTGCCAGC -AGCC-GCGG TAATTCCAGC T-CCAATAGC
C_hominis_KR296813       ATCAATTGGA GGGCAA-GTC TGGTGCCAGC -AGCC-GCGG TAATTCCAGC T-CCAATAGC
C_hominis_AF112569       ATCAATTGGA GGGCAA-GTC TGGTGCCAGC -AGCC-GCGG TAATTCCAGC T-CCAATAGC
C_hominis_KF679723       ATCAATTGGA GGGCAA-GTC TGGTGCCAGC -AGCC-GCGG TAATTCCAGC T-CCAATAGC
24937-C.hominis          ATCAATTGGA GGGCAA-GTC TGGTGCCAGC -AGCC-GCGG TAATTCCAGC T-CCAATAGC
C_parvum_KP204486        ATCAATTGGA GGGCAA-GTC TGGTGCCAGC -AGCC-GCGG TAATTCCAGC T-CCAATAGC
C_parvum_KM012042        ATCAATTGGA GGGCAA-GTC TGGTGCCAGC -AGCC-GCGG TAATTCCAGC T-CCAATAGC
C_parvum_AY268582        ATCAATTGGA GGGCAA-GTC TGGTGCCAGC -AGCC-GCGG TAATTCCAGC T-CCAATAGC
C_parvum_KM012045        ATCAATTGGA GGGCAA-GTC TGGTGCCAGC -AGCC-GCGG TAATTCCAGC T-CCAATAGC
C_parvum_KM012043        ATCAATTGGA GGGCAA-GTC TGGTGCCAGC -AGCC-GCGG TAATTCCAGC T-CCAATAGC
C_parvum_AF108864        ATCAATTGGA GGGCAA-GTC TGGTGCCAGC -AGCC-GCGG TAATTCCAGC T-CCAATAGC
C_parvum_KM085018        ATCAATTGGA GGGCAA-GTC TGGTGCCAGC -AGCC-GCGG TAATTCCAGC T-CCAATAGC
C_parvum_LC01201         ATCAATTGGA GGGCAA-GTC TGGTGCCAGC -AGCC-GCGG TAATTCCAGC T-CCAATAGC
```

```
C_parvum_KJ808688        ATCAATTGGA GGGCAA-GTC TGGTGCCAGC -AGCC-GCGG TAATTCCAGC T-CCAATAGC
C_parvum_LC012016        ATCAATTGGA GGGCAA-GTC TGGTGCCAGC -AGCC-GCGG TAATTCCAGC T-CCAATAGC
C_parvum_KM012040        ATCAATTGGA GGGCAA-GTC TGGTGCCAGC -AGCC-GCGG TAATTCCAGC T-CCAATAGC
C_parvum_KJ808687        ATCAATTGGA GGGCAA-GTC TGGTGCCAGC -AGCC-GCGG TAATTCCAGC T-CCAATAGC
C_parvum_KM012044        ATCAATTGGA GGGCAA-GTC TGGTGCCAGC -AGCC-GCGG TAATTCCAGC T-CCAATAGC
C_parvum_KP334136        ATCAATTGGA GGGCAA-GTC TGGTGCCAGC -AGCC-GCGG TAATTCCAGC T-CCAATAGC
C_parvum_KJ808689        ATCAATTGGA GGGCAA-GTC TGGTGCCAGC -AGCC-GCGG TAATTCCAGC T-CCAATAGC
C_parvum_KM012046        ATCAATTGGA GGGCAA-GTC TGGTGCCAGC -AGCC-GCGG TAATTCCAGC T-CCAATAGC
C_parvum_LC012015        ATCAATTGGA GGGCAA-GTC TGGTGCCAGC -AGCC-GCGG TAATTCCAGC T-CCAATAGC
C_parvum_K16X154         ATCAATTGGA GGGCAA-GTC TGGTGCCAGC -AGCC-GCGG TAATTCCAGC T-CCAATAGC
C_parvum_KJ808691        ATCAATTGGA GGGCAA-GTC TGGTGCCAGC -AGCC-GCGG TAATTCCAGC T-CCAATAGC
C_parvum_KU892559        ATCAATTGGA GGGCAA-GTC TGGTGCCAGC -AGCC-GCGG TAATTCCAGC T-CCAATAGC
C_parvum_AB968048        ATCAATTGGA GGGCAA-GTC TGGTGCCAGC -AGCC-GCGG TAATTCCAGC T-CCAATAGC
C_parvum_KJ808690        ATCAATTGGA GGGCAA-GTC TGGTGCCAGC -AGCC-GCGG TAATTCCAGC T-CCAATAGC
C_parvum_KJ808692        ATCAATTGGA GGGCAA-GTC TGGTGCCAGC -AGCC-GCGG TAATTCCAGC T-CCAATAGC
C_parvum_AF093493        ATCAATTGGA GGGCAA-GTC TGGTGCCAGC -AGCC-GCGG TAATTCCAGC T-CCAATAGC
C_parvum_EU553550        ATCAATTGGA GGGCAA-GTC TGGTGCCAGC -AGCC-GCGG TAATTCCAGC T-CCAATAGC
C_parvum_EU553557        ATCAATTGGA GGGCAA-GTC TGGTGCCAGC -AGCC-GCGG TAATTCCAGC T-CCAATAGC
C_parvum_KJ808693        ATCAATTGGA GGGCAA-GTC TGGTGCCAGC -AGCC-GCGG TAATTCCAGC T-CCAATAGC
C_parvum_KJ808694        ATCAATTGGA GGGCAA-GTC TGGTGCCAGC -AGCC-GCGG TAATTCCAGC T-CCAATAGC
C_parvum_KJ808695        ATCAATTGGA GGGCAA-GTC TGGTGCCAGC -AGCC-GCGG TAATTCCAGC T-CCAATAGC
25115-C.parvum           ATCAATTGGA GGGCAA-GTC TGGTGCCAGC -AGCC-GCGG TAATTCCAGC T-CCAATAGC
C_parvum_KP004204        ATCAATTGGA GGGCAA-GTC TGGTGCCAGC -AGCC-GCGG TAATTCCAGC T-CCAATAGC
C_parvum_KP004203        ATCAATTGGA GGGCAA-GTC TGGTGCCAGC -AGCC-GCGG TAATTCCAGC T-CCAATAGC
C_parvum_AB271070        ---------- ---------- ---------- ---------- ---------- ----------


                         ....|....| ....|....| ....|....| ....|....| ....|....| ....|....|
                                430        440        450        460        470        480
25330-C.ubiquitum        GTATATTAAA GTTGTTGCAG TTAAAAAGCT CGTAGTTGGA TTTCTGTTA- ATAATTT-AT
C_ubiquitum_KC608030     GTATATTAAA GTTGTTGCAG TTAAAAAGCT CGTAGTTGGA TTTCTGTTA- ATAATTT-AT
C_ubiquitum_KC962124     GTATATTAAA GTTGTTGCAG TTAAAAAGCT CGTAGTTGGA TTTCTGTTA- ATAATTT-AT
C_ubiquitum_AB697056     GTATATTAAA GTTGTTGCAG TTAAAAAGCT CGTAGTTGGA TTTCTGTTA- ATAATTT-AT
C_ubiquitum_KU531665     GTATATTAAA GTTGTTGCAG TTAAAAAGCT CGTAGTTGGA TTTCTGTTA- ATAATTT-AT
C_felis_JQ312664         GTATATTAAA GTTGTTGCAG TTAAAAAGCT CGTAGTTGGA TTTCTGTTA- ATACCTT-AT
C_felis_AJ493211         GTATATTAAA GTTGTTGCAG TTAAAAAGCT CGTAGTTGGA TTTCTGTTA- ATACCTT-AT
C_felis_JN833576         GTATATTAAA GTTGTTGCAG TTAAAAAGCT CGTAGTTGGA TTTCTGTTA- ATACCTT-AT
C_felis_AF159113         GTATATTAAA GTTGTTGCAG TTAAAAAGCT CGTAGTTGGA TTTCTGTTA- ATACCTT-AT
C_felis_KT749819         GTATATTAAA GTTGTTGCAG TTAAAAAGCT CGTAGTTGGA TTTCTGTTA- ATACCTT-AT
C_felis_FJ707310         GTATATTAAA GTTGTTGCAG TTAAAAAGCT CGTAGTTGGA TTTCTGTTA- ATACCTT-AT
C_felis_KM977642         GTATATTAAA GTTGTTGCAG TTAAAAAGCT CGTAGTTGGA TTTCTGTTA- ATACCTT-AT
C_muris_GU319781         GTATATTAAA GTTGTTGCAG TTAAAAAGCT CGTAGTTGGA TTTCTGTTGT ATAATCT-AT
C_muris_KF419208         GTATATTAAA GTTGTTGCAG TTAAAAAGCT CGTAGTTGGA TTTCTGTTGT ATAATCT-AT
C_muris_EU553592         GTATATTAAA GTTGTTGCAG TTAAAAAGCT CGTAGTTGGA TTTCTGTTGT ATAATCT-AT
C_muris_GU319783         GTATATTAAA GTTGTTGCAG TTAAAAAGCT CGTAGTTGGA TTTCTGTTGT ATAATCT-AT
C_muris_EU245045         GTATATTAAA GTTGTTGCAG TTAAAAAGCT CGTAGTTGGA TTTCTGTTGT ATAATCT-AT
C_muris_GQ227706         GTATATTAAA GTTGTTGCAG TTAAAAAGCT CGTAGTTGGA TTTCTGTTGT ATAATCT-AT
C_muris_EU156446         GTATATTAAA GTTGTTGCAG TTAAAAAGCT CGTAGTTGGA TTTCTGTTGT ATAATCT-AT
C_muris_AY642591         GTATATTAAA GTTGTTGCAG TTAAAAAGCT CGTAGTTGGA TTTCTGTTGT ATAATCT-AT
C_muris_KY490555         GTATATTAAA GTTGTTGCAG TTAAAAAGCT CGTAGTTGGA TTTCTGTTGT ATAATCTTAT
23767-C.muris            GTATATTAAA GTTGTTGCAG TTAAAAAGCT CGTAGTTGGA TTCCTGTTGT ATAATCT-AT
C_andersoni_AB449819     GTATATTAAA GTTGTTGCAG TTAAAAAGCT CGTAGTTGGA TTTCTGTTGT ATAATTT-AT
C_andersoni_EF613341     ---------- ---------- ---------- --------GA TTTCTGTTGT ATAATTTTAT
C_andersoni_KF826306     GTATATTAAA GTTGTTGCAG TTAAAAAGCT CGTAGTTGGA TTTCTGTTGT ATAATTTTAT
C_andersoni_KT922229     GTATATTAAA GTTGTTGCAG TTAAAAAGCT CGTAGTTGGA TTTCTGTTGT ATAATTTTAT
C_andersoni_KT884487     GTATATTAAA GTTGTTGCAG TTAAAAAGCT CGTAGTTGGA TTTCTGTTGT ATAATTTTAT
C_andersoni_LC012014     GTATATTAAA GTTGTTGCAG TTAAAAAGCT CGTAGTTGGA TTTCTGTTGT ATAATTTTAT
C_andersoni_AB449816     GTATATTAAA GTTGTTGCAG TTAAAAAGCT CGTAGTTGGA TTTCTGTTGT ATAATTTTAT
C_andersoni_AY954886     GTATATTAAA GTTGTTGCAG TTAAAAAGCT GGTAGTTGGA TTTCTGTTGT ATAATTT-AT
C_andersoni_AY954885     GTATATTAAA GTTGTTGCAG TTAAAAAGCT CGTAGTTGGA TTTCTGTTGT ATAATTT-AT
C_andersoni_AB089285     GTATATTAAA GTTGTTGCAG TTAAAAAGCT CGTAGTTGGA TTTCTGTTGT ATAATTT-AT
C_andersoni_LC012013     GTATATTAAA GTTGTTGCAG TTAAAAAGCT CGTAGTTGGA TTTCTGTTGT ATAATTT-AT
C_andersoni_KF826307     GTATATTAAA GTTGTTGCAG TTAAAAAGCT CGTAGTTGGA TTTCTGTTGT ATAATTT-AT
C_andersoni_KF826305     GTATATTAAA GTTGTTGCAG TTAAAAAGCT CGTAGTTGGA TTTCTGTTGT ATAATTT-AT
C_andersoni_KF826304     GTATATTAAA GTTGTTGCAG TTAAAAAGCT CGTAGTTGGA TTTCTGTTGT ATAATTT-AT
C_andersoni_EU245042     GTATATTAAA GTTGTTGCAG TTAAAAAGCT CGTAGTTGGA TTTCTGTTGT ATAATTT-AT
C_andersoni_KT922228     GTATATTAAA GTTGTTGCAG TTAAAAAGCT CGTAGTTGGA TTTCTGTTGT ATAATTT-AT
C_andersoni_KT884488     GTATATTAAA GTTGTTGCAG TTAAAAAGCT CGTAGTTGGA TTTCTGTTGT ATAATTT-AT
C_andersoni_AB449817     GTATATTAAA GTTGTTGCAG TTAAAAAGCT CGTAGTTGGA TTTCTGTTGT ATAATTT-AT
C_andersoni_KT922230     GTATATTAAA GTTGTTGCAG TTAAAAAGCT CGTAGTTGGA TTTCTGTTGT ATAATTT-AT
C_hominis_KF826315       GTATATTAAA GTTGTTGCAG TTAAAAAGCT CGTAGTTGGA TTTCTGTTA- ATAATTT-AT
```

```
C_hominis_HQ149022      GTATATTAAA GTTGTTGCAG TTAAAAAGCT CGTAGTTGGA TTTCTGTTA- ATAATTT-AT
C_hominis_KR296813      GTATATTAAA GTTGTTGCAG TTAAAAAGCT CGTAGTTGGA TTTCTGTTA- ATAATTT-AT
C_hominis_AF112569      GTATATTAAA GTTGTTGCAG TTAAAAAGCT CGTAGTTGGA TTTCTGTTA- ATAATTT-AT
C_hominis_KF679723      GTATATTAAA GTTGTTGCAG TTAAAAAGCT CGTAGTTGGA TTTCTGTTA- ATAATTT-AT
24937-C.hominis         GTATATTAAA GTTGTTGCAG TTAAAAAGCT CGTAGTTGGA TTTCTGTTA- ATAATTT-AT
C_parvum_KP204486       GTATATTAAA GTTGTTGCAG TTAAAAAGCT CGTAGTTGGA TTTCTGTTA- ATAATTT-AT
C_parvum_KM012042       GTATATTAAA GTTGTTGCAG TTAAAAAGCT CGTAGTTGGA TTTCTGTTA- ATAATTT-AT
C_parvum_AY268582       GTATATTAAA GTTGTTGCAG TTAAAAAGCT CGTAGTTGGA TTTCTGTTA- ATAATTT-AT
C_parvum_KM012045       GTATATTAAA GTTGTTGCAG TTAAAAAGCT CGTAGTTGGA TTTCTGTTA- ATAATTT-AT
C_parvum_KM012043       GTATATTAAA GTTGTTGCAG TTAAAAAGCT CGTAGTTGGA TTTCTGTTA- ATAATTT-AT
C_parvum_AF108864       GTATATTAAA GTTGTTGCAG TTAAAAAGCT CGTAGTTGGA TTTCTGTTA- ATAATTT-AT
C_parvum_KM085018       GTATATTAAA GTTGTTGCAG TTAAAAAGCT CGTAGTTGGA TTTCTGTTA- ATAATTT-AT
C_parvum_LC01201        GTATATTAAA GTTGTTGCAG TTAAAAAGCT CGTAGTTGGA TTTCTGTTA- ATAATTT-AT
C_parvum_KJ808688       GTATATTAAA GTTGTTGCAG TTAAAAAGCT CGTAGTTGGA TTTCTGTTA- ATAATTT-AT
C_parvum_LC012016       GTATATTAAA GTTGTTGCAG TTAAAAAGCT CGTAGTTGGA TTTCTGTTA- ATAATTT-AT
C_parvum_KM012040       GTATATTAAA GTTGTTGCAG TTAAAAAGCT CGTAGTTGGA TTTCTGTTA- ATAATTT-AT
C_parvum_KJ808687       GTATATTAAA GTTGTTGCAG TTAAAAAGCT CGTAGTTGGA TTTCTGTTA- ATAATTT-AT
C_parvum_KM012044       GTATATTAAA GTTGTTGCAG TTAAAAAGCT CGTAGTTGGA TTTCTGTTA- ATAATTT-AT
C_parvum_KP334136       GTATATTAAA GTTGTTGCAG TTAAAAAGCT CGTAGTTGGA TTTCTGTTA- ATAATTT-AT
C_parvum_KJ808689       GTATATTAAA GTTGTTGCAG TTAAAAAGCT CGTAGTTGGA TTTCTGTTA- ATAATTT-AT
C_parvum_KM012046       GTATATTAAA GTTGTTGCAG TTAAAAAGCT CGTAGTTGGA TTTCTGTTA- ATAATTT-AT
C_parvum_LC012015       GTATATTAAA GTTGTTGCAG TTAAAAAGCT CGTAGTTGGA TTTCTGTTA- ATAATTT-AT
C_parvum_K16X154        GTATATTAAA GTTGTTGCAG TTAAAAAGCT CGTAGTTGGA TTTCTGTTA- ATAATTT-AT
C_parvum_KJ808691       GTATATTAAA GTTGTTGCAG TTAAAAAGCT CGTAGTTGGA TTTCTGTTA- ATAATTT-AT
C_parvum_KU892559       GTATATTAAA GTTGTTGCAG TTAAAAAGCT CGTAGTTGGA TTTCTGTTA- ATAATTT-AT
C_parvum_AB968048       GTATATTAAA GTTGTTGCAG TTAAAAAGCT CGTAGTTGGA TTTCTGTTA- ATAATTT-AT
C_parvum_KJ808690       GTATATTAAA GTTGTTGCAG TTAAAAAGCT CGTAGTTGGA TTTCTGTTA- ATAATTT-AT
C_parvum_KJ808692       GTATATTAAA GTTGTTGCAG TTAAAAAGCT CGTAGTTGGA TTTCTGTTA- ATAATTT-AT
C_parvum_AF093493       GTATATTAAA GTTGTTGCAG TTAAAAAGCT CGTAGTTGGA TTTCTGTTA- ATAATTT-AT
C_parvum_EU553550       GTATATTAAA GTTGTTGCAG TTAAAAAGCT CGTAGTTGGA TTTCTGTTA- ATAATTT-AT
C_parvum_EU553557       GTATATTAAA GTTGTTGCAG TTAAAAAGCT CGTAGTTGGA TTTCTGTTA- ATAATTT-AT
C_parvum_KJ808693       GTATATTAAA GTTGTTGCAG TTAAAAAGCT CGTAGTTGGA TTTCTGTTA- ATAATTT-AT
C_parvum_KJ808694       GTATATTAAA GTTGTTGCAG TTAAAAAGCT CGTAGTTGGA TTTCTGTTA- ATAATTT-AT
C_parvum_KJ808695       GTATATTAAA GTTGTTGCAG TTAAAAAGCT CGTAGTTGGA TTTCTGTTA- ATAATTT-AT
25115-C.parvum          GTATATTAAA GTTGTTGCAG TTAAAAAGCT CGTAGTTGGA TTTCTGTTA- ATAATTT-AT
C_parvum_KP004204       GTATATTAAA GTTGTTGCAG TTAAAAAGCT CGTAGTTGGA TTTCTGTTA- ATAATTT-AT
C_parvum_KP004203       GTATATTAAA GTTGTTGCAG TTAAAAAGCT CGTAGTTGGA TTTCTGTTA- ATAATTT-AT
C_parvum_AB271070       ---------- ---------- ------AGCT CGTAGTTGGA TTTCTGTTA- ATAATTT-AT


                        ....|....| ....|....| ....|....| ....|....| ....|....| ....|....|
                                490        500        510        520        530        540
25330-C.ubiquitum       ATATAATATT TTAT----TA ATATTTAT-- ATAGTATTAA CATAATTCAT ATTACT----
C_ubiquitum_KC608030    ATATAATATT TTAT----TA ATATTTAT-- ATAGTATTAA CATAATTCAT ATTACT----
C_ubiquitum_KC962124    ATATAATATT TTAA----AG ATATTTAT-- ATAATATTAA CATAATTCAT ATTACT----
C_ubiquitum_AB697056    ATATAATATT TTAA----TG ATATTTAT-- ATAATATTAA CATAATTCAT ATTACT----
C_ubiquitum_KU531665    ATACAATATT TTAT----TA ATATTTAT-- ATAGTATTAA CATAATTCAT ATTACT----
C_felis_JQ312664        ATATAATATT TTTTT---AA ATATTAATAT GTAAGATTAA CAT---TCAT ATTTTT-AAG
C_felis_AJ493211        ATATAATATT TTTTTTTTAA ATATTAATAT GTAAGATTAA CATAATTCAT ATTTTT-AAG
C_felis_JN833576        ATATAATATT TTTTTTT-AA ATATTAATAT GTAAGATTAA CATAATTCAT ATTTTT-AAG
C_felis_AF159113        ATATAATATT TTTTTTTTAA ATATTAATAT GTAAGATTAA CATAATTCAT ATTTTT-AAG
C_felis_KT749819        ATATAATATT TTTTTTT-AA ATATTAATAT GTAAGATTAA CATAATTCAT ATTTTT-AAG
C_felis_FJ707310        ATATAATATT TTTTTTT-AA ATATTAATAT GTAAGATTAA CATAATTCAT ATTTTT-AAG
C_felis_KM977642        ATATAATATT TTTTTTT-AA ATATTATTAT GTAAGATTAA CATAATTCAT ATTTTTTAAG
C_muris_GU319781        A-ATATTACT A-------AG GTATATAT-T ATATTATCAA CATCCTTCCT ATTAT-----
C_muris_KF419208        A-ATATTACT A-------AG GTATATAT-T ATATTATCAA CATCCTTCCT ATTAT-----
C_muris_EU553592        A-ATATTACT A-------AG GTATATAT-T ATATTATCAA CATCCTTCCT ATTAT-----
C_muris_GU319783        A-ATATTACT A-------AG GTATATAT-T ATATTATCAA CATCCTTCCT ATTAT-----
C_muris_EU245045        A-ATATTACT A-------AG GTATATAT-T ATATTATCAA CATCCTTCCT ATTAT-----
C_muris_GQ227706        A-ATATTACT A-------AG GTATATAT-T ATATTATCAA CATCCTTCCT ATTAT-----
C_muris_EU156446        A-ATATTACT A-------AG GTATATAT-T ATATTATCGA CATCCTTCCT ATTAT-----
C_muris_AY642591        A-ATATTACC A-------AG GTATATAT-T ATATTATCAA CATCCTTCCT ATTAT-----
C_muris_KY490555        A-ATATTACT A-------AG GTATATAT-T ATATTATCAA CATCCTTCCT ATTAT-----
23767-C.muris           A-ATATTACT A-------AG GTATATAT-T ATATTATCAA CATCCTTCCT ATTAT-----
C_andersoni_AB449819    A-ATATTACC A-------AG GTAATTAT-T ATATTATCAA CATCCTTCCT ATTAT-----
C_andersoni_EF613341    A-ATATTACC A-------AG GTAATTAT-T ATATTATCAA CATCCTTCCT ATTAT-----
C_andersoni_KF826306    A-ATATTACC A-------AG GTAATTAT-T ATATTATCAA CATCCTTCCT ATTAT-----
C_andersoni_KT922229    A-ATATTACC A-------AG GTAATTAT-T ATATTATCAA CATCCTTCCT ATTAT-----
C_andersoni_KT884487    A-ATATTACC A-------AG GTAATTAT-T ATATTATCAA CATCCTTCCT ATTAT-----
C_andersoni_LC012014    A-ATATTACC A-------AG GTAATTAT-T ATATTATCAA CATCCTTCCT ATTAT-----
C_andersoni_AB449816    A-ATATTACC A-------AG GTAATTAT-T ATATTATCAA CATCCTTCCT ATTAT-----
```

```
C_andersoni_AY954886   A-ATATTACC A-------AG GTAATTAT-T ATATTATCAA CATCCTTCCT ATTAT-----
C_andersoni_AY954885   A-ATATTACC A-------AG GTAATTAT-T ATATTATCAA CATCCTTCCT ATTAT-----
C_andersoni_AB089285   A-ATATTACC A-------AG GTAATTAT-T ATATTATCAA CATCCTTCCT ATTAT-----
C_andersoni_LC012013   A-ATATTACC A-------AG GTAATTAT-T ATATTATCAA CATCCTTCCT ATTAT-----
C_andersoni_KF826307   A-ATATTACC A-------AG GTAATTAT-T ATATTATCAA CATCCTTCCT ATTAT-----
C_andersoni_KF826305   A-ATATTACC A-------AG GTAATTAT-T ATATTATCAA CATCCTTCCT ATTAT-----
C_andersoni_KF826304   A-ATATTACC A-------AG GTAATTAT-T ATATTATCAA CATCCTTCCT ATTAT-----
C_andersoni_EU245042   A-ATATTACC A-------AG GTAATTAT-T ATATTATCAA CATCCTTCCT ATTAT-----
C_andersoni_KT922228   A-ATATTACC A-------AG GTAATTAT-T ATATTATCAA CATCCTTCCT ATTAT-----
C_andersoni_KT884488   A-ATATTACC A-------AG GTAATTAT-T ATATTATCAA CATCCTTCCT ATTAT-----
C_andersoni_AB449817   A-ATATTACC A-------AG GTAATTAT-T ATATTATCAA CATCCTTCCT ATTAT-----
C_andersoni_KT922230   A-ATATTACC A-------AG GTAATTAT-T ATATTATCAA CATCCTTCCT ATTAT-----
C_hominis_KF826315     ATAAAATATT TTGAT---GA ATATTTAT-- ATAATATTAA CATAATTCAT ATTACT----
C_hominis_HQ149022     ATAATATATT TTGAT---GA ATATTTAT-- ATAATATTAA CATAATTCAT ATTACT----
C_hominis_KR296813     ATAATATATT TTGAT---GA ATATTTAT-- ATAATATTAA CATAATTCAT ATTACT----
C_hominis_AF112569     ATAATATATT TTGAT---GA ATATTTAT-- ATAATATTAA CATAATTCAT ATTACT----
C_hominis_KF679723     ATAATGTATT TTGAT---GA ATATTTAT-- ATAATATTAA CATAATTCAT ATTACT----
24937-C.hominis        ATAATATATT TTGAT---GA ATATTTAT-- ATAATATTAA CATGATTCAT ATTACT----
C_parvum_KP204486      ATAAAATATT TTGAT---GA ATATTTAT-- ATAATATTAA CATAATTCAT ATTACT----
C_parvum_KM012042      ATAAAATATT TTGAT---GA ATATTTAT-- ATAATATTAA CATAATTCAT ATTACT----
C_parvum_AY268582      ATAAAATATT TTGAT---GA ATATTTAT-- ATAATATTAA CATAATTCAT ATTACT----
C_parvum_KM012045      ATAAAATATT TTGAT---GA ATATTTAT-- ATAATATTAA CATAATTCAT ATTACT----
C_parvum_KM012043      ATAAAATATT TTGAT---GA ATATTTAT-- ATAATATTAA CATAATTCAT ATTACT----
C_parvum_AF108864      ATAAAATATT TTGAT---GA ATATTTAT-- ATAATATTAA CATAATTCAT ATTACT----
C_parvum_KM085018      ATAAAATATT TTGAT---GA ATATTTAT-- ATAATATTAA CATAATTCAT ATTACT----
C_parvum_LC01201       ATAAAATATT TTGAT---GA ATATTTAT-- ATAATATTAA CATAATTCAT ATTACT----
C_parvum_KJ808688      ATAAAATATT TTGAT---GA ATATTTAT-- ATAATATTAA CATAATTCAT ATTACT----
C_parvum_LC012016      ATAAAATATT TTGAT---GA ATATTTAT-- ATAATATTAA CATAATTCAT ATTACT----
C_parvum_KM012040      ATAAAATATT TTGAT---GA ATATTTAT-- ATAATATTAA CATAATTCAT ATTACT----
C_parvum_KJ808687      ATAAAATATT TTGAT---GA ATATTTAT-- ATAATATTAA CATAATTCAT ATTACT----
C_parvum_KM012044      ATAAAATATT TTGAT---GA ATATTTAT-- ATAATATTAA CATAATTCAT ATTACT----
C_parvum_KP334136      ATAAAATATT TTGAT---GA ATATTTAT-- ATAATATTAA CATAATTCAT ATTACT----
C_parvum_KJ808689      ATAAAATATT TTGAT---GA ATATTTAT-- ATAATATTAA CATAATTCAT ATTACT----
C_parvum_KM012046      ATAAAATATT TTGAT---GA ATATTTAT-- ATAATATTAA CATAATTCAT ATTACT----
C_parvum_LC012015      ATAAAATATT TTGAT---GA ATATTTAT-- ATAATATTAA CATAATTCAT ATTACT----
C_parvum_K16X154       ATAAAATATT TTGAT---GA ATATTTAT-- ATAATATTAA CATAATTCAT ATTACT----
C_parvum_KJ808691      ATAAAATATT TTGAT---GA ATATTTAT-- ATAATATTAA CATAATTCAT ATTACT----
C_parvum_KU892559      ATAAAATATT TTGAT---GA ATATTTAT-- ATAATATTAA CATAATTCAT ATTACT----
C_parvum_AB968048      ATAAAATATT TTGAT---GA ATATTTAT-- ATAATATTAA CATAATTCAT ATTACT----
C_parvum_KJ808690      ATAAAATATT TTGAT---GA ATATTTAT-- ATAATATTAA CATAATTCAT ATTACT----
C_parvum_KJ808692      ATAAAATATT TTGAT---GA ATATTTAT-- ATAATATTAA CATAATTCAT ATTACT----
C_parvum_AF093493      ATAAAATATT TTGAT---GA ATATTTAT-- ATAATATTAA CATAATTCAT ATTACT----
C_parvum_EU553550      ATAAAATATT TTGA------ ATATTTAT-- ATAACATTAA CATAATTCAT ATTACT----
C_parvum_EU553557      ATAAAATATT TTGA------ ATATTTAT-- ATAACATTAA CATAATTCAT ATTACT----
C_parvum_KJ808693      ATAAAATATT TTGA------ ATATTTAT-- ATAACATTAA CATAATTCAT ATTACT----
C_parvum_KJ808694      ATAAAATATT TTGA------ ATATTTAT-- ATAACATTAA CATAATTCAT ATTACT----
C_parvum_KJ808695      ATAAAATATT TTGA------ ATATTTAT-- ATAACATTAA CATAATTCAT ATTACT----
25115-C.parvum         ATAAAATATT TTGAT---GA ATATTTAT-- ATAATATTAA CATAATTCAT ATTACT----
C_parvum_KP004204      ATAAAATATT TTGAT---GA ATATTTAT-- ATAATATTAA CATAATTCAT ATTACT----
C_parvum_KP004203      ATAAAATATT TT------GA ATATTTAT-- ATAACATTAA CATAATTCAT ATTACT----
C_parvum_AB271070      ATAAAATATT TT------GA ATATTTAT-- ATAACATTAA CATAATTCAT ATTACT----


                       ....|....| ....|....| ....|....| ....|....| ....|....| ....|....|
                               550        560        570        580        590        600
25330-C.ubiquitum      -----ATATT TTAT----AG TATATGAAAT TTTACTTTGA GAAAATTAGA GTGCTTAAAG
C_ubiquitum_KC608030   -----A--TT TTAT----AG TATATGAAAT TTTACTTTGA GAAAATTAGA GTGCTTAAAG
C_ubiquitum_KC962124   -----AA--- TTAA--TTAG TATATGAAAT TTTACTTTGA GAAAATTAGA GTGCTTAAAG
C_ubiquitum_AB697056   -----AAATT TTTT--ATAG TATATGAAAT TTTACTTTGA GAAAATTAGA GTGCTTAAAG
C_ubiquitum_KU531665   -----ATTTT TTTT--ATAG TATGTGAAAT TTTACTTTGA GAAAATTAGA GTGCTTAAAG
C_felis_JQ312664       ACTGAATTTT TAGTTTTGAT AATATGAAAT TTTACTTTGA GAAAATTAGA GTGCTTAAA-
C_felis_AJ493211       ACTGAATTTT TAGTTTCGAT AATATGAAAT TTTACTTTGA GAAAATTAGA GTGCTTAAAG
C_felis_JN833576       ACTGAATTTT TAGTTTTGAT AATATGAAAT TTTACTTTGA GAAAATTAGA GTGCTTAAAG
C_felis_AF159113       ACTGAATTTT TAGTTTTGAT AATATGAAAT TTTACTTTGA GAAAATTAGA GTGCTTAAAG
C_felis_KT749819       ACTGAATTTT TAGTTTTGAT AATATGAAAT TTTACTTTGA GAAAATTAGA GTGCTTAAAG
C_felis_FJ707310       ACTGAATTTT TAGTTTTGAT AATATGAAAT TTTACTTTGA GAAAATTAGA GTGCTTAAAG
C_felis_KM977642       ACTGAATTTT TAGTTTTGAT AATATGAAAT TTTACTTTGA GAAAATTAGA GTGCTTAAAG
C_muris_GU319781       -----ATTTC TAAATAT--- -ATAGGAAAC TTTACTTTGA GAAAATTAGA GTGCTTAAAG
C_muris_KF419208       -----ATTTC TAAATAT--- -ATAGGAAAC TTTACTTTGA GAAAATTAGA GTGCTTAAAG
C_muris_EU553592       -----ATTTC TAAATAT--- -ATAGGAAAC TTTACTTTGA GAAAATTAGA GTGCTTAAAG
C_muris_GU319783       -----ATTTC TAAATAT--- -ATAGGAAAC TTTACTTTGA GAAAATTAGA GTGCTTAAAG
```

```
C_muris_EU245045         -----ATTTC TAAATAT--- -ATAGGAAAC TTTACTTTGA GAAAATTAGA GTGCTTAAAG
C_muris_GQ227706         -----ATTTC TAAATAT--- -ATAGGAAAC TTTACTTTGA GAAAATTAGA GTGCTTAAAG
C_muris_EU156446         -----ATATC TAAATAT--- -ATAGGAAAC TTTACTTTGA GAAAATTAGA GTGCTTAAAG
C_muris_AY642591         -----ATT-C TAAATAT--- -ATAGGAAAT TTTACTTTGA GAAAATTAGA GTGCTTAAAG
C_muris_KY490555         -----ATCTC TAAATAT--- -ATAGGAAAC TTTACTTTGA GAAAATTAGA GTGCTTAAAG
23767-C.muris            -----ATTTC TAAATAT--- -ATAGGAAAC TTTACTTTGA GAAAATTAGA GTGCTTAAAG
C_andersoni_AB449819     -----AT-TC TAAATAT--- -ATAGGAAAT TTTACTTTGA GAAAATTAGA GTGCTTAAAG
C_andersoni_EF613341     -----ATT-C TAAATAT--- -ATAGGAAAT TTTACTTTGA GAAAATTAGA GTGCTTAAAG
C_andersoni_KF826306     -----ATT-C TAAATAT--- -ATAGGAAAT TTTACTTTGA GAAAATTAGA GTGCTTAAAG
C_andersoni_KT922229     -----ATT-C TAAATAT--- -ATAGGAAAT TTTACTTTGA GAAAATTAGA GTGCTTAAAG
C_andersoni_KT884487     -----ATT-C TAAATAT--- -ATAGGAAAT TTTACTTTGA GAAAATTAGA GTGCTTAAAG
C_andersoni_LC012014     -----ATT-C TAAATAT--- -ATAGGAAAT TTTACTTTGA GAAAATTAGA GTGCTTAAAG
C_andersoni_AB449816     -----ATT-C TAAATAT--- -ATAGGAAAT TTTACTTTGA GAAAATTAGA GTGCTTAAAG
C_andersoni_AY954886     -----ATT-C TAAATAT--- -ATAGGAAAT TTTACTTTGA GAAAATTAGA GTGCTTAAAG
C_andersoni_AY954885     -----ATT-C TAAATAT--- -ATAGGAAAT TTTACTTTGA GAAAATTAGA GTGCTTAAAG
C_andersoni_AB089285     -----ATT-C TAAATAT--- -ATAGGAAAT TTTACTTTGA GAAAATTAGA GTGCTTAAAG
C_andersoni_LC012013     -----ATT-C TAAATAT--- -ATAGGAAAT TTTACTTTGA GAAAATTAGA GTGCTTAAAG
C_andersoni_KF826307     -----ATT-C TAAATAT--- -ATAGGAAAT TTTACTTTGA GAAAATTAGA GTGCTTAAAG
C_andersoni_KF826305     -----ATT-C TAAATAT--- -ATAGGAAAT TTTACTTTGA GAAAATTAGA GTGCTTAAAG
C_andersoni_KF826304     -----ATT-C TAAATAT--- -ATAGGAAAT TTTACTTTGA GAAAATTAGA GTGCTTAAAG
C_andersoni_EU245042     -----ATT-C TAAATAT--- -ATAGGAAAT TTTACTTTGA GAAAATTAGA GTGCTTAAAG
C_andersoni_KT922228     -----ATT-C TAAATAT--- -ATAGGAAAT TTTACTTTGA GAAAATTAGA GTGCTTAAAG
C_andersoni_KT884488     -----ATT-C TAAATAT--- -ATAGGAAAT TTTACTTTGA GAAAATTAGA GTGCTTAAAG
C_andersoni_AB449817     -----ATT-C TAAATAT--- -ATAGGAAAT TTTACTTTGA GAAAATTAGA GTGCTTAAAG
C_andersoni_KT922230     -----ATT-C TAAATAT--- -ATAGGAAAT TTTACTTTGA GAAAATTAGA GTGCTTAAAG
C_hominis_KF826315       -----ATTTT TTTTTTT-AG AATATGAAAT TTTACTTTGA AAAAATTAGG GTGCTTAAAG
C_hominis_HQ149022       -----ATTTT TTTTTTA--G TATATGAAAT TTTACTTTGA GAAAATTAGA GTGCTTAAAG
C_hominis_KR296813       -----ATTTT TTTTTAG--- TATATGAAAT TTTACTTTGA GAAAATTAGA GTGCTTAAAG
C_hominis_AF112569       -----ATTTT TTTTTAG--- TATATGAAAT TTTACTTTGA GAAAATTAGA GTGCTTAAAG
C_hominis_KF679723       -----ATTTT TTTTTAG--- TATATGAAAT TTTACTTTGA GAAAATTAGA GTGCTTAAAG
24937-C.hominis          -----ATTTT TTTTTAG--- TATATGAAAT TTTACTTTGA GAAAATTAGA GTGCTTAAAG
C_parvum_KP204486        -----ATATA TTTTAGT--- -ATATGAAAT TTTACTTTGA GAAAATTAGA GTGCTTAAAG
C_parvum_KM012042        -----ATATA TTTTAGT--- -ATATGAAAT TTTACTTTGA GAAAATTAGA GTGCTTAAAG
C_parvum_AY268582        -----ATATA TTTTAGT--- -ATATGAAAT TTTACTTTGA GAAAATTAGA GTGCTTAAAG
C_parvum_KM012045        -----ATATA TTTTAGT--- -ATATGAAAT TTTACTTTGA GAAAATTAGA GTGCTTAAAG
C_parvum_KM012043        -----ATATA TTTTAGT--- -ATATGAAAT TTTACTTTGA GAAAATTAGA GTGCTTAAAG
C_parvum_AF108864        -----ATATA TTTTAGT--- -ATATGAAAT TTTACTTTGA GAAAATTAGA GTGCTTAAAG
C_parvum_KM085018        -----ATATA TTTTAGT--- -ATATGAAAT TTTACTTTGA GAAAATTAGA GTGCTTAAAG
C_parvum_LC01201         -----ATATA TTTTAGT--- -ATATGAAAT TTTACTTTGA GAAAATTAGA GTGCTTAAAG
C_parvum_KJ808688        -----ATATA TTTTAGT--- -ATATGAAAT TTTACTTTGA GAAAATTAGA GTGCTTAAAG
C_parvum_LC012016        -----ATATA TTTTAGT--- -ATATGAAAT TTTACTTTGA GAAAATTAGA GTGCTTAAAG
C_parvum_KM012040        -----ATATA TTTTAGT--- -ATATGAAAT TTTACTTTGA GAAAATTAGA GTGCTTAAAG
C_parvum_KJ808687        -----ATATA TTTTAGT--- -ATATGAAAT TTTACTTTGA GAAAATTAGA GTGCTTAAAG
C_parvum_KM012044        -----ATATA TTTTAGT--- -ATATGAAAT TTTACTTTGA GAAAATTAGA GTGCTTAAAG
C_parvum_KP334136        -----ATATA TTTTAGT--- -ATATGAAAT TTTACTTTGA GAAAATTAGA GTGCTTAAAG
C_parvum_KJ808689        -----ATATA TTTTAGT--- -ATATGAAAT TTTACTTTGA GAAAATTAGA GTGCTTAAAG
C_parvum_KM012046        -----ATATA TTTTAGT--- -ATATGAAAT TTTACTTTGA GAAAATTAGA GTGCTTAAAG
C_parvum_LC012015        -----ATATA TTTTAGT--- -ATATGAAAT TTTACTTTGA GAAAATTAGA GTGCTTAAAG
C_parvum_K16X154         -----ATATA TTTTAGT--- -ATATGAAAT TTTACTTTGA GAAAATTAGA GTGCTTAAAG
C_parvum_KJ808691        -----ATATA TTTTAGT--- -ATATGAAAT TTTACTTTGA GAAAATTAGA GTGCTTAAAG
C_parvum_KU892559        -----ATATA TTTTAGT--- -ATATGAAAT TTTACTTTGA GAAAATTAGA GTGCTTAAAG
C_parvum_AB968048        -----ATATA TTTTAGT--- -ATATGAAAT TTTACTTTGA GAAAATTAGA GTGCTTAAAG
C_parvum_KJ808690        -----ATATA TTTTAGT--- -ATATGAAAT TTTACTTTGA GAAAATTAGA GTGCTTAAAG
C_parvum_KJ808692        -----ATATA TTTTAGT--- -ATATGAAAT TTTACTTTGA GAAAATTAGA GTGCTTAAAG
C_parvum_AF093493        -----ATATA TTTTAGT--- -ATATGAAAT TTTACTTTGA GAAAATTAGA GTGCTTAAAG
C_parvum_EU553550        -----ATATT TTTTAGT--- -ATATGAAAT TTTACTTTGA GAAAATTAGA GTGCTTAAAG
C_parvum_EU553557        -----ATATT TTTTAGT--- -ATATGAAAT TTTACTTTGA GAAAATTAGA GTGCTTAAAG
C_parvum_KJ808693        -----ATATT TTTTAGT--- -ATATGAAAT TTTACTTTGA GAAAATTAGA GTGCTTAAAG
C_parvum_KJ808694        -----ATATT TTTTAGT--- -ATATGAAAT TTTACTTTGA GAAAATTAGA GTGCTTAAAG
C_parvum_KJ808695        -----ATATT TTTTAGT--- -ATATGAAAT TTTACTTTGA GAAAATTAGA GTGCTTAAAG
25115-C.parvum           -----ATATA TTTTAGT--- -ATATGAAAT TTTACTTTGA GAAAATTAGA GTGCTTAAAG
C_parvum_KP004204        -----ATATA TTTTAGT--- -ATATGAAAT TTTACTTTGA GAAAATTAGA GTGCTTAAAG
C_parvum_KP004203        -----ATATT TTTTAGT--- -ATATGAAAT TTTACTTTGA GAAAATTAGA GTGCTTAAAG
C_parvum_AB271070        -----ATATT TTTTAGT--- -ATATGAAAT TTTACTTTGA GAAAATTAGA GTGCTTAAAG


                         ....|....| ....|....| ....|....| ....|....| ....|....| ....|....|
                                610        620        630        640        650        660
25330-C.ubiquitum        CAGGCAT--T AGCCTTGAAT ACTCCAGC-A TGGAA-TAAT AT-AAAAG-A TTTTTATCTT
C_ubiquitum_KC608030     CAGGCAT--T AGCCTTGAAT ACTCCAGC-A TGGAA-TAAT AT-AAAAG-A TTTTTATCTT
C_ubiquitum_KC962124     CAGGCAT--T AGCCTTGAAT ACTCCAGC-A TGGAA-TAAT AT-AAAAG-A TTTTTATCTT
```

**C_ubiquitum_AB697056**	CAGGCTT--T	AGCCTTGAAT	ACTCCAGC-A	TGGAA-TAAT	AT-AAAAG-A	TTTTTATCTT
**C_ubiquitum_KU531665**	CAGGCATATT	AGCCTTGAAT	ACTCCAGC-A	TGGAA-TAAT	AT-AAAAG-A	TTTTTATCTT
**C_felis_JQ312664**	CAGGCTT--T	TCCCTTGAAT	ACTCCAGC-A	TGGAA-TAAT	AATAAAAG-A	TTTTTATCTT
**C_felis_AJ493211**	CAGGCTT--T	TGCCTTGAAT	ACTCCAGC-A	TGGAA-TAAT	AATAAAAG-A	TTTTTATCTT
**C_felis_JN833576**	CAGGCTT--T	TGCCTTGAAT	ACTCCAGC-A	TGGAA-TAAT	AATAAAAG-A	TTTTTATCTT
**C_felis_AF159113**	CAGGCTT--T	TGCCTTGAAT	ACTCCAGC-A	TGGAA-TAAT	AATAAAAG-A	TTTTTATCTT
**C_felis_KT749819**	CAGGCTT--T	TGCCTTGAAT	ACTCCAGC-A	TGGAA-TAAT	AATAAAAG-A	TTTTTATCTT
**C_felis_FJ707310**	CAGGCTT--T	TGCCTTGAAT	ACTCCAGC-A	TGGAA-TAAT	AATAAAAG-A	TTTTTATCTT
**C_felis_KM977642**	CAGGCTT--T	TGCCTTGAAT	ACTCCAGC-A	TGGAA-TAAT	AATAAAAG-A	TTTTTATCTT
**C_muris_GU319781**	CAGGCAA--C	TGCCTTGAAT	ACTCCAGC-A	TGGAA-TAAT	AAGTAAGG-A	CTTTTGTCTT
**C_muris_KF419208**	CAGGCAA--C	TGCCTTGAAT	ACTCCAGC-A	TGGAA-TAAT	AAGTAAGG-A	CTTTTGTCTT
**C_muris_EU553592**	CAGGCAA--C	TGCCTTGAAT	ACTCCAGC-A	TGGAA-TAAT	AAGTAAGG-A	CTTTTGTCTT
**C_muris_GU319783**	CAGGCAA--C	TGCCTTGAAT	ACTCCAGC-A	TGGAA-TAAT	AAGTAAGG-A	CTTTTGTCTT
**C_muris_EU245045**	CAGGCAA--C	TGCCTTGAAT	ACTCCAGC-A	TGGAA-TAAT	AAGTAAGG-A	CTTTTGTCTT
**C_muris_GQ227706**	CAGGCAA--C	TGCCTTGAAT	ACTCCAGC-A	TGGAA-TAAT	AAGTAAGG-A	CTTTTGTCTT
**C_muris_EU156446**	CAGGCAA--C	TGCCTTGAAT	ACTCCAGC-A	TGGAA-TAAT	AAGTAAGG-A	CTTTTGTCTT
**C_muris_AY642591**	CAGGCAA--C	TGCCTTGAAT	ACTCCAGC-A	TGGAA-TAAT	AAGTAAGG-A	CTTTTGTCTT
**C_muris_KY490555**	CAGGCAA--C	TGCCTTGAAT	ACTCCAGC-A	TGGAA-TAAT	AAGTAAGG-A	CTTTTGTCTT
**23767-C.muris**	CAGGCAA--C	TGCCTTGAAT	ACTCCAGC-A	TGGAA-TAAT	AAGTAAGG-A	CTTTTGTCTT
**C_andersoni_AB449819**	CAGGCAA--C	TGCCTTGAAT	ACTCCAGC-A	TGGAA-TAAT	AAGTAAGG-A	CTTTTGTCTT
**C_andersoni_EF613341**	CAGGCAA--C	TGCCTTGAAT	ACTCCAGC-A	TGGAA-TAAT	AAGTAAGG-A	CTTTTGTCTT
**C_andersoni_KF826306**	CAGGCAA--C	TGCCTTGAAT	ACTCCAGC-A	TGGAA-TAAT	AAGTAAGG-A	CTTTTGTCTT
**C_andersoni_KT922229**	CAGGCAA--C	TGCCTTGAAT	ACTCCAGC-A	TGGAA-TAAT	AAGTAAGG-A	CTTTTGTCTT
**C_andersoni_KT884487**	CAGGCAA--C	TGCCTTGAAT	ACTCCAGC-A	TGGAA-TAAT	AAGTAAGG-A	CTTTTGTCTT
**C_andersoni_LC012014**	CAGGCAA--C	TGCCTTGAAT	ACTCCAGC-A	TGGAA-TAAT	AAGTAAGG-A	CTTTTGTCTT
**C_andersoni_AB449816**	CAGGCAA--C	TGCCTTGAAT	ACTCCAGC-A	TGGAA-TAAT	AAGTAAGG-A	CTTTTGTCTT
**C_andersoni_AY954886**	CAGGCAA--C	TGCCTTGAAT	ACTCCAGC-A	TGGAA-TAAT	AAGTAAGG-A	CTTTTGTCTT
**C_andersoni_AY954885**	CAGGCAA--C	TGCCTTGAAT	ACTCCAGC-A	TGGAA-TAAT	AAGTAAGG-A	CTTTTGTCTT
**C_andersoni_AB089285**	CAGGCAA--C	TGCCTTGAAT	ACTCCAGC-A	TGGAA-TAAT	AAGTAAGG-A	CTTTTGTCTT
**C_andersoni_LC012013**	CAGGCAA--C	TGCCTTGAAT	ACTCCAGC-A	TGGAA-TAAT	AAGTAAGG-A	CTTTTGTCTT
**C_andersoni_KF826307**	CAGGCAA--C	TGCCTTGAAT	ACTCCAGC-A	TGGAA-TAAT	AAGTAAGG-A	CTTTTGTCTT
**C_andersoni_KF826305**	CAGGCAA--C	TGCCTTGAAT	ACTCCAGC-A	TGGAA-TAAT	AAGTAAGG-A	CTTTTGTCTT
**C_andersoni_KF826304**	CAGGCAA--C	TGCCTTGAAT	ACTCCAGC-A	TGGAA-TAAT	AAGTAAGG-A	CTTTTGTCTT
**C_andersoni_EU245042**	CAGGCAA--C	TGCCTTGAAT	ACTCCAGC-A	TGGAA-TAAT	AAGTAAGG-A	CTTTTGTCTT
**C_andersoni_KT922228**	CAGGCAA--C	TGCCTTGAAT	ACTCCAGC-A	TGGAA-TAAT	AAGTAAGG-A	CTTTTGTCTT
**C_andersoni_KT884488**	CAGGCAA--C	TGCCTTGAAT	ACTCCAGC-A	TGGAA-TAAT	AAGTAAGG-A	CTTTTGTCTT
**C_andersoni_AB449817**	CAGGCAA--C	TGCCTTGAAT	ACTCCAGC-A	TGGAA-TAAT	AAGTAAGG-A	CTTTTGTCTT
**C_andersoni_KT922230**	CAGGCAA--C	TGCCTTGAAT	ACTCCAGC-A	TGGAA-TAAT	AAGTAAGG-A	CTTTTGTCTT
**C_hominis_KF826315**	CAGGATT--A	TGCCTTGAAT	ACTCCAGC-A	TGGAA-TAAT	AT-TAAAG-A	TTTTTATCTT
**C_hominis_HQ149022**	CAGGCAT--A	TGCCTTGAAT	ACTCCAGC-A	TGGAA-TAAT	AT-TAAAG-A	TTTTTATCTT
**C_hominis_KR296813**	CAGGCAT--A	TGCCTTGAAT	ACTCCAGC-A	TGGAA-TAAT	AT-TAAAG-A	TTTTTATCTT
**C_hominis_AF112569**	CAGGCAT--A	TGCCTTGAAT	ACTCCAGC-A	TGGAA-TAAT	AT-TAAAG-A	TTTTTATCTT
**C_hominis_KF679723**	CAGGCAT--A	TGCCTTGAAT	ACTCCAGC-A	TGGAA-TAAT	AT-TAAAG-A	TTTTTATCTT
**24937-C.hominis**	CAGGCAT--A	TGCCTTGAAT	ACTCCAGC-A	TGGAA-TAAT	AT-TAAAG-A	TTTTTATCTT
**C_parvum_KP204486**	CAGGCAT--A	TGCCTTGAAT	ACTCCAGC-A	TGGAA-TAAT	AT-TAAAG-A	TTTTTATCTT
**C_parvum_KM012042**	CAGGCAT--A	TGCCTTGAAT	ACTCCAGC-A	TGGAA-TAAT	AT-TAAAG-A	TTTTTATCTT
**C_parvum_AY268582**	CAGGCAT--A	TGCCTTGAAT	ACTCCAGC-A	TGGAA-TAAT	AT-TAAAG-A	TTTTTATCTT
**C_parvum_KM012045**	CAGGCAT--A	TGCCTTGAAT	ACTCCAGC-A	TGGAA-TAAT	AT-TAAAG-A	TTTTTATCTT
**C_parvum_KM012043**	CAGGCAT--A	TGCCTTGAAT	ACTCCAGC-A	TGGAA-TAAT	AT-TAAAG-A	TTTTTATCTT
**C_parvum_AF108864**	CAGGCAT--A	TGCCTTGAAT	ACTCCAGC-A	TGGAA-TAAT	AT-TAAAG-A	TTTTTATCTT
**C_parvum_KM085018**	CAGGCAT--A	TGCCTTGAAT	ACTCCAGC-A	TGGAA-TAAT	AT-TAAAG-A	TTTTTATCTT
**C_parvum_LC01201**	CAGGCAT--A	TGCCTTGAAT	ACTCCAGC-A	TGGAA-TAAT	AT-TAAAG-A	TTTTTATCTT
**C_parvum_KJ808688**	CAGGCAT--A	TGCCTTGAAT	ACTCCAGC-A	TGGAA-TAAT	AT-TAAAG-A	TTTTTATCTT
**C_parvum_LC012016**	CAGGCAT--A	TGCCTTGAAT	ACTCCAGC-A	TGGAA-TAAT	AT-TAAAG-A	TTTTTATCTT
**C_parvum_KM012040**	CAGGCAT--A	TGCCTTGAAT	ACTCCAGC-A	TGGAA-TAAT	AT-TAAAG-A	TTTTTATCTT
**C_parvum_KJ808687**	CAGGCAT--A	TGCCTTGAAT	ACTCCAGC-A	TGGAA-TAAT	AT-TAAAG-A	TTTTTATCTT
**C_parvum_KM012044**	CAGGCAT--A	TGCCTTGAAT	ACTCCAGC-A	TGGAA-TAAT	AT-TAAAG-A	TTTTTATCTT
**C_parvum_KP334136**	CAGGCAT--A	TGCCTTGAAT	ACTCCAGC-A	TGGAA-TAAT	AT-TAAAG-A	TTTTTATCTT
**C_parvum_KJ808689**	CAGGCAT--A	TGCCTTGAAT	ACTCCAGC-A	TGGAA-TAAT	AT-TAAAG-A	TTTTTATCTT
**C_parvum_KM012046**	CAGGCAT--A	TGCCTTGAAT	ACTCCAGC-A	TGGAA-TAAT	AT-TAAAG-A	TTTTTATCTT
**C_parvum_LC012015**	CAGGCAT--A	TGCCTTGAAT	ACTCCAGC-A	TGGAA-TAAT	AT-TAAAG-A	TTTTTATCTT
**C_parvum_K16X154**	CAGGCAT--A	TGCCTTGAAT	ACTCCAGC-A	TGGAA-TAAT	AT-TAAAG-A	TTTTTATCTT
**C_parvum_KJ808691**	CAGGCAT--A	TGCCTTGAAT	ACTCCAGC-A	TGGAA-TAAT	AT-TAAAG-A	TTTTTATCTT
**C_parvum_KU892559**	CAGGCAT--A	TGCCTTGAAT	ACTCCAGC-A	TGGAA-TAAT	AT-TAAAG-A	TTTTTATCTT
**C_parvum_AB968048**	CAGGCAT--A	TGCCTTGAAT	ACTCCAGC-A	TGGAA-TAAT	AT-TAAAG-A	TTTTTATCTT
**C_parvum_KJ808690**	CAGGCAT--A	TGCCTTGAAT	ACTCCAGC-A	TGGAA-TAAT	AT-TAAAG-A	TTTTTATCTT
**C_parvum_KJ808692**	CAGGCAT--A	TGCCTTGAAT	ACTCCAGC-A	TGGAA-TAAT	AT-TAAAG-A	TTTTTATCTT
**C_parvum_AF093493**	CAGGCAT--A	TGCCTTGAAT	ACTCCAGC-A	TGGAA-TAAT	AT-TAAAG-A	TTTTTATCTT
**C_parvum_EU553550**	CAGGCAT--A	TGCCTTGAAT	ACTCCAGC-A	TGGAA-TAAT	AT-TAAAG-A	TTTTTATCTT
**C_parvum_EU553557**	CAGGCAT--A	TGCCTTGAAT	ACTCCAGC-A	TGGAA-TAAT	AT-TAAAG-A	TTTTTATCTT
**C_parvum_KJ808693**	CAGGCAT--A	TGCCTTGAAT	ACTCCAGC-A	TGGAA-TAAT	AT-TAAAG-A	TTTTTATCTT

```
C_parvum_KJ808694         CAGGCAT--A TGCCTTGAAT ACTCCAGC-A TGGAA-TAAT AT-TAAAG-A TTTTTATCTT
C_parvum_KJ808695         CAGGCAT--A TGCCTTGAAT ACTCCAGC-A TGGAA-TAAT AT-TAAAG-A TTTTTATCTT
25115-C.parvum            CAGGCAT--A TGCCTTGAAT ACTCCAGC-A TGGAA-TAAT AT-TAAAG-A TTTTTATCTT
C_parvum_KP004204         CAGGCAT--A TGCCTTGAAT ACTCCAGC-A TGGAA-TAAT AT-TAAAG-A TTTTTATCTT
C_parvum_KP004203         CAGGCAT--A TGCCTTGAAT ACTCCAGC-A TGGAA-TAAT AT-TAAAG-A TTTTTATCTT
C_parvum_AB271070         CAGGCAT--A TGCCTTGAAT ACTCCAGC-A TGGAA-TAAT AT-TAAAG-A TTTTTATCTT


                          ....|....| ....|....| ....|....| ....|....| ....|....| ....|....|
                                 670        680        690        700        710        720
25330-C.ubiquitum         TTTT--ATTG GTTCTAAGAT AAAAATAATG ATTAATAGGG ACAGTTGGGG GCAT--TTGT
C_ubiquitum_KC608030      TTTT--ATTG GTTCTAAGAT AAAAATAATG ATTAATAGGG ACAGTTGGGG GCAT--TTGT
C_ubiquitum_KC962124      TTTT--ATTG GTTCTAGGAT AAAAATAATG ATTAATAGGG ACAGTTGGGG GCAT--TTGT
C_ubiquitum_AB697056      TTTT--ATTG GTTCTAAGAT AAAAATAATG ATTAATAGGG ACAGTTGGGG GCAT--TTGT
C_ubiquitum_KU531665      TTTT--ATTG GTTCTAAGAT AAAAATAATG ATTAATAGGG ACAGTTGGGG GCAT--T---
C_felis_JQ312664          TTTTT-ATTG GTTCTAAGAT AAAAATAATG ATTAATAGGG ACAGTTGGGG GCAT--TTGT
C_felis_AJ493211          TTTTT-ATTG GTTCTAAGAT AAAAATAATG ATTAATAGGG ACAGTTGGGG GCAT--TTGT
C_felis_JN833576          TTTTTTATTG GTTCTAAGAT AAAAATAATG ATTAATAGGG ACAGTTGGGG GCAT--TTGT
C_felis_AF159113          TTTTTTATTG GTTCTAAGAT AAAAATAATG ATTAATAGGG ACAGTTGGGG GCAT--TTGT
C_felis_KT749819          TTTTTTATTG GTTCTAAGAT AAAAATAATG ATTAATAGGG ACAGTTGGGG GCAT--TTGT
C_felis_FJ707310          TTTTTTATTG GTTCTAAGAT AAAAATAATG ATTAATAGGG ACAGTTGGGG GCAT--TTGT
C_felis_KM977642          TTTTTTATTG GTTCTAAGAT AAAAATAATG ATTAATAGGG ACAGTTGGGG GCAT--TTGT
C_muris_GU319781          TCTT--ATTG GTTCTAGGAC AAAAGTAATG GTTAATAGGG ACAGTTGGGG GCAT--TCGT
C_muris_KF419208          TCTT--ATTG GTTCTAGGAC AAAAGTAATG GTTAATAGGG ACAGTTGGGG GCAT--TCGT
C_muris_EU553592          TCTT--ATTG GTTCTAGGAC AAAAGTAATG GTTAATAGGG ACAGTTGGGG GCAT--TCGT
C_muris_GU319783          TCTT--ATTG GTTCTAGGAC AAAAGTAATG GTTAATAGGG ACAGTTGGGG GCAT--TCGT
C_muris_EU245045          TCTT--ATTG GTTCTAGGAC AAAAGTAATG GTTAATAGGG ACAGTTGGGG GCAT--TCGT
C_muris_GQ227706          TCTT--ATTG GTTCTAGGAC AAAAGTAATG GTTAATAGGG ACAGTTGGGG GCAT--TCGT
C_muris_EU156446          TCTT--ATTG GTTCTAGGAC AAAAGTAATG GTTAATAGGG ACAGTTGGGG GCAT--TCGT
C_muris_AY642591          TCTT--ATTG GTTCTAGGAC AAAAGTAATG GTTAATAGGG ACAGTTGGGG GCAT--TCGT
C_muris_KY490555          TCTT--ATTG GTTCTAGGAC AAAAGTAATG GTTAATAGGG ACAGTTGGGG GCAT--TCGT
23767-C.muris             TCTT--ATTG GTTCTAGGAC AAAAGTAATG GTTAATAGGG ACAGTTGGGG GCAT--TCGT
C_andersoni_AB449819      TCTT--ATTG GTTCTAGGAC AAAAGTAATG GTTAATAGGG ACAGTTGGGG GCAT--TCGT
C_andersoni_EF613341      TCTT--ATTG GTTCTAGGAC AAAAGTAATG GTTAATAGGG ACAGTTGGGG GCAT--TCGT
C_andersoni_KF826306      TCTT--ATTG GTTCTAGGAC AAAAGTAATG GTTAATAGGG ACAGTTGGGG GCAT--TCGT
C_andersoni_KT922229      TCTT--ATTG GTTCTAGGAC AAAAGTAATG GTTAATAGGG ACAGTTGGGG GCAT--TCGT
C_andersoni_KT884487      TCTT--ATTG GTTCTAGGAC AAAAGTAATG GTTAATAGGG ACAGTTGGGG GCAT--TCGT
C_andersoni_LC012014      TCTT--ATTG GTTCTAGGAC AAAAGTAATG GTTAATAGGG ACAGTTGGGG GCAT--TCGT
C_andersoni_AB449816      TCTT--ATTG GTTCTAGGAC AAAAGTAATG GTTAATAGGG ACAGTTGGGG GCAT--TCGT
C_andersoni_AY954886      TCTT--ATTG GTTCTAGGAC AAAAGTAATG GTTAATAGGG ACAGTTGGGG GCAT--TCGT
C_andersoni_AY954885      TCTT--ATTG GTTCTAGGAC AAAAGTAATG GTTAATAGGG ACAGTTGGGG GCAT--TCGT
C_andersoni_AB089285      TCTT--ATTG GTTCTAGGAC AAAAGTAATG GTTAATAGGG ACAGTTGGGG GCAT--TCGT
C_andersoni_LC012013      TCTT--ATTG GTTCTAGGAC AAAAGTAATG GTTAATAGGG ACAGTTGGGG GCAT--TCGT
C_andersoni_KF826307      TCTT--ATTG GTTCTAGGAC AAAAGTAATG GTTAATAGGG ACAGTTGGGG GCAT--TCGT
C_andersoni_KF826305      TCTT--ATTG GTTCTAGGAC AAAAGTAATG GTTAATAGGG ACAGTTGGGG GCAT--TCGT
C_andersoni_KF826304      TCTT--ATTG GTTCTAGGAC AAAAGTAATG GTTAATAGGG ACAGTTGGGG GCAT--TCGT
C_andersoni_EU245042      TCTT--ATTG GTTCTAGGAC AAAAGTAATG GTTAATAGGG ACAGTTGGGG GCAT--TCGT
C_andersoni_KT922228      TCTT--ATTG GTTCTAGGAC AAAAGTAATG GTTAATAGGG ACAGTTGGGG GCAT--TCGT
C_andersoni_KT884488      TCTT--ATTG GTTCTAGGAC AAAAGTAATG GTTAATAGGG ACAGTTGGGG GCAT--TCGT
C_andersoni_AB449817      TCTT--ATTG GTTCTAGGAC AAAAGTAATG GTTAATAGGG ACAGTTGGGG GCAT--TCGT
C_andersoni_KT922230      TCTT--ATTG GTTCTAGGAC AAAAGTAATG GTTAATAGGG ACAGTTGGGG GCAT--TCGT
C_hominis_KF826315        TTTT--ATTG GTTCTAAAAT AAAAATAATG ATTAATAGGG ACAGTTGGGG GCAT--TTGT
C_hominis_HQ149022        TTTT--ATTG GTTCTAAGAT AAGAATAATG ATTAATAGGG ACAGTTGGGG GCAT--TTGT
C_hominis_KR296813        TTTT--ATTG GTTCTAAGAT AAGAATAATG ATTAATAGGG ACAGTTGGGG GCAT--TTGT
C_hominis_AF112569        TTTT--ATTG GTTCTAAGAT AAGAATAATG ATTAATAGGG ACAGTTGGGG GCAT--TTGT
C_hominis_KF679723        TTTT--ATTG GTTCTAAGAT AAGAATAATG ATTAATAGGG ACAGTTGGGG GCAT--TTGT
24937-C.hominis           TCTT--ATTG GTTCTAAGAT AAGAATGATG ATTAATAGGG ACAGTTGGGG GCAT--TTGT
C_parvum_KP204486         TCTT--ATTG GTTCTAAGAT AAGAATAATG ATTAATAGGG ACAGTTGGGG GCAT--TTGT
C_parvum_KM012042         TCTT--ATTG GTTCTAAGAT AAGAATAATG ATTAATAGGG ACAGTTGGGG GCAT--TTGT
C_parvum_AY268582         TCTT--ATTG GTTCTAAGAT AAGAATAATG ATTAATAGGG ACAGTTGGGG GCAT--TTGT
C_parvum_KM012045         TCTT--ATTG GTTCTAAGAT AAGAATAATG ATTAATAGGG ACAGTTGGGG GCAT--TTGT
C_parvum_KM012043         TCTT--ATTG GTTCTAAGAT AAGAATAATG ATTAATAGGG ACAGTTGGGG GCAT--TTGT
C_parvum_AF108864         TCTT--ATTG GTTCTAAGAT AAGAATAATG ATTAATAGGG ACAGTTGGGG GCAT--TTGT
C_parvum_KM085018         TCTT--ATTG GTTCTAAGAT AAGAATAATG ATTAATAGGG ACAGTTGGGG GCAT--TTGT
C_parvum_LC01201          TCTT--ATTG GTTCTAAGAT AAGAATAATG ATTAATAGGG ACAGTTGGGG GCAT--TTGT
C_parvum_KJ808688         TCTT--ATTG GTTCTAAGAT AAGAATAATG ATTAATAGGG ACAGTTGGGG GCAT--TTGT
C_parvum_LC012016         TCTT--ATTG GTTCTAAGAT AAGAATAATG ATTAATAGGG ACAGTTGGGG GCAT--TTGT
C_parvum_KM012040         TCTT--ATTG GTTCTAAGAT AAGAATAATG ATTAATAGGG ACAGTTGGGG GCAT--TTGT
C_parvum_KJ808687         TCTT--ATTG GTTCTAAGAT AAGAATAATG ATTAATAGGG ACAGTTGGGG GCAT--TTGT
C_parvum_KM012044         TCTT--ATTG GTTCTAAGAT AAGAATAATG ATTAATAGGG ACAGTTGGGG GCAT--TTGT
C_parvum_KP334136         TCTT--ATTG GTTCTAAGAT AAGAATAATG ATTAATAGGG ACAGTTGGGG GCAT--TTGT
```

```
C_parvum_KJ808689        TCTT--ATTG GTTCTAAGAT AAGAATAATG ATTAATAGGG ACAGTTGGGG GCAT--TTGT
C_parvum_KM012046        TCTT--ATTG GTTCTAAGAT AAGAATAATG ATTAATAGGG ACAGTTGGGG GCAT--TTGT
C_parvum_LC012015        TCTT--ATTG GTTCTAAGAT AAGAATAATG ATTAATAGGG ACAGTTGGGG GCAT--TTGT
C_parvum_K16X154         TCTT--ATTG GTTCTAAGAT AAGAATAATG ATTAATAGGG ACAGTTGGGG GCAT--TTGT
C_parvum_KJ808691        TCTT--ATTG GTTCTAAGAT AAGAATAATG ATTAATAGGG ACAGTTGGGG GCAT--TTGT
C_parvum_KU892559        TCTT--ATTG GTTCTAAGAT AAGAATAATG ATTAATAGGG ACAGTTGGGG GCAT--TTGT
C_parvum_AB968048        TCTT--ATTG GTTCTAAGAT AAGAATAATG ATTAATAGGG ACAGTTGGGG GCAT--TTGT
C_parvum_KJ808690        TCTT--ATTG GTTCTAAGAT AAGAATAATG ATTAATAGGG ACAGTTGGGG GCAT--TTGT
C_parvum_KJ808692        TCTT--ATTG GTTCTAAGAT AAGAATAATG ATTAATAGGG ACAGTTGGGG GCAT--TTGT
C_parvum_AF093493        TCTT--ATTG GTTCTAAGAT AAGAATAATG ATTAATAGGG ACAGTTGGGG GCAT--TTGT
C_parvum_EU553550        TCTT--ATTG GTTCTAAGAT AAGAATAATG ATTAATAGGG ACAGTTGGGG GCAT--TTGT
C_parvum_EU553557        TCTT--ATTG GTTCTAAGAT AAGAATAATG ATTAATAGGG ACAGTTGGGG GCAT--TTGT
C_parvum_KJ808693        TCTT--ATTG GTTCTAAGAT AAGAATAATG ATTAATAGGG ACAGTTGGGG GCAT--TTGT
C_parvum_KJ808694        TCTT--ATTG GTTCTAAGAT AAGAATAATG ATTAATAGGG ACAGTTGGGG GCAT--TTGT
C_parvum_KJ808695        TCTT--ATTG GTTCTAAGAT AAGAATAATG ATTAATAGGG ACAGTTGGGG GCAT--TTGT
25115-C.parvum           TCTT--ATTG GTTCTAAGAT AAGAATAATG ATTAATAGGG ACAGTTGGGG GCAT--TTGT
C_parvum_KP004204        TCTT--ATTG GTTCTAAGAT AAGAATAATG ATTAATAGGG ACAGTTGGGG GCAT--TTGT
C_parvum_KP004203        TCTT--ATTG GTTCTAAGAT AAGAATAATG ATTAATAGGG ACAGTTGGGG GCAT--TTGT
C_parvum_AB271070        TCTT--ATTG GTTCTAAGAT AAGAATAATG ATTAATAGGG ACAGTTGGGG GCAT--TTGT


                         ....|....| ....|....| ....|....| ....|....| ....|....| ....|....|
                                730        740        750        760        770        780
25330-C.ubiquitum        ATTTAACAGT C-AGAGGTGA AATTCTTAGA TTTG--TTAA AGACAAACTA GTGCGAAAGC
C_ubiquitum_KC608030     ATTTAACAGT C-AGAGGTGA AATTCTTAGA TTTG--TTAA AGACAAACTA GTGCGAAAGC
C_ubiquitum_KC962124     ATTTAACAGT C-AGAGGTGA AATTCTTAGA TTTG--TTAA AGACAAACTA GTGCGAAAGC
C_ubiquitum_AB697056     ATTTAACAGT C-AGAGGTGA AATTCTTAGA TTTG--TTAA AGACAAACTA GTGCGAAAGC
C_ubiquitum_KU531665     ---------- ---------- ---------- ---------- ---------- ----------
C_felis_JQ312664         ATTTAACAGT C-AGAGGTGA TATTCTTAGA TTTG--TTAA AGACAAACTA ATGCGAAAGC
C_felis_AJ493211         ATTTAACAGT C-AGAGGTGA TATTCTTAGA TTTG--TTAA AGACAAACTA ATGCGAAAGC
C_felis_JN833576         ATTTAACAGT C-AGAGGTGA TATTCTTAGA TTTG--TTAA AGACAAACTA ATGCGAAAGC
C_felis_AF159113         ATTTAACAGT C-AGAGGTGA TATTCTTAGA TTTG--TTAA AGACAAACTA ATGCGAAAGC
C_felis_KT749819         ATTTAACAGT C-AGAGGTGA TATTCTTAGA TTTG--TTAA AGACAAACTA ATGCGAAAGC
C_felis_FJ707310         ATTTAACAGT C-AGAGGTGA TATTCTTAGA TTTG--TTAA AGACAAACTA ATGCGAAAGC
C_felis_KM977642         ATTTAACAGT C-AGAGGTGA TATTCTTAGA TTTG--TTAA AGACAAACTA ATGCGAAAGC
C_muris_GU319781         ATTTAACAGC C-AGAGGTGA AATTCTTAGA TTTG--TTAA AGACGAACTA CTGCGAAAGC
C_muris_KF419208         ATTTAACAGC C-AGAGGTGA AATTCTTAGA TTTG--TTAA AGACGAACTA CTGCGAAAGC
C_muris_EU553592         ATTTAACAGC C-AGAGGTGA AATTCTTAGA TTTG--TTAA AGACGAACTA CTGCGAAAGC
C_muris_GU319783         ATTTAACAGC C-AGAGGTGA AATTCTTAGA TTTG--TTAA AGACGAACTA CTGCGAAAGC
C_muris_EU245045         ATTTAACAGC C-AGAGGTGA AATTCTTAGA TTTG--TTAA AGACGAACTA CTGCGAAAGC
C_muris_GQ227706         ATTTAACAGC C-AGAGGTGA AATTCTTAGA TTTG--TTAA AGACGAACTA CTGCGAAAGC
C_muris_EU156446         ATTTAACAGC C-AGAGGTGA AATTCTTAGA TTTG--TTAA AGACGAACTA CTGCGAAAGC
C_muris_AY642591         ATTTAACAGC C-AGAGGTGA AATTCTTAGA TTTG--TTAA AGACGAACTA CTGCGAAAGC
C_muris_KY490555         ATTTAACAGC C-AGAGGTGA AATTCTTAGA TTTG--TTAA AGACGAACTA CTGCGAAAGC
23767-C.muris            ATTTAACAGC C-AGAGGTGA AATTCTTAGA TTTG--TTAA AGACGAACTA CTGCGAAAGC
C_andersoni_AB449819     ATTTAACAGC C-AGAGGTGA AATTCTTAGA TTTG--TTAA AGACGAACTA CTGCGAAAGC
C_andersoni_EF613341     ATTTAACAGC C-AGAGGTGA AATTCTTAGA TTTG--TTAA AGACGAACTA CTGCGAAAGC
C_andersoni_KF826306     ATTTAACAGC C-AGAGGTGA AATTCTTAGA TTTG--TTAA AGACGAACTA CTGCGAAAGC
C_andersoni_KT922229     ATTTAACAGC C-AGAGGTGA AATTCTTAGA TTTG--TTAA AGACGAACTA CTGCGAAAGC
C_andersoni_KT884487     ATTTAACAGC C-AGAGGTGA AATTCTTAGA TTTG--TTAA AGACGAACTA CTGCGAAAGC
C_andersoni_LC012014     ATTTAACAGC C-AGAGGTGA AATTCTTAGA TTTG--TTAA AGACGAACTA CTGCGAAAGC
C_andersoni_AB449816     ATTTAACAGC C-AGAGGTGA AATTCTTAGA TTTG--TTAA AGACGAACTA CTGCGAAAGC
C_andersoni_AY954886     ATTTAACAGC C-AGAGGTGA AATTCTTAGA TTTG--TTAA AGACGAACTA CTGCGAAAGC
C_andersoni_AY954885     ATTTAACAGC C-AGAGGTGA AATTCTTAGA TTTG--TTAA AGACGAACTA CTGCGAAAGC
C_andersoni_AB089285     ATTTAACAGC C-AGAGGTGA AATTCTTAGA TTTG--TTAA AGACGAACTA CTGCGAAAGC
C_andersoni_LC012013     ATTTAACAGC C-AGAGGTGA AATTCTTAGA TTTG--TTAA AGACGAACTA CTGCGAAAGC
C_andersoni_KF826307     ATTTAACAGC C-AGAGGTGA AATTCTTAGA TTTG--TTAA AGACGAACTA CTGCGAAAGC
C_andersoni_KF826305     ATTTAACAGC C-AGAGGTGA AATTCTTAGA TTTG--TTAA AGACGAACTA CTGCGAAAGC
C_andersoni_KF826304     ATTTAACAGC C-AGAGGTGA AATTCTTAGA TTTG--TTAA AGACGAACTA CTGCGAAAGC
C_andersoni_EU245042     ATTTAACAGC C-AGAGGTGA AATTCTTAGA TTTG--TTAA AGACGAACTA CTGCGAAAGC
C_andersoni_KT922228     ATTTAACAGC C-AGAGGTGA AATTCTTAGA TTTG--TTAA AGACGAACTA CTGCGAAAGC
C_andersoni_KT884488     ATTTAACAGC C-AGAGGTGA AATTCTTAGA TTTG--TTAA AGACGAACTA CTGCGAAAGC
C_andersoni_AB449817     ATTTAACAGC C-AGAGGTGA AATTCTTAGA TTTG--TTAA AGACGAACTA CTGCGAAAGC
C_andersoni_KT922230     ATTTAACAGC C-AGAGGTGA AATTCTTAGA TTTG--TTAA AGACGAACTA CTGCGAAAGC
C_hominis_KF826315       TTTTAACAGT C-AGAGGTGA AATTCTTATA TTTG--TTAA AGACAAACTA ATGCGAAAGC
C_hominis_HQ149022       ATTTAACAGT C-AGAGGTGA AATTCTTAGA TTTG--TTAA AGACAAACTA ATGCGAAAGC
C_hominis_KR296813       ATTTAACAGT C-AGAGGTGA AATTCTTAGA TTTG--TTAA AGACAAACTA ATGCGAAAGC
C_hominis_AF112569       ATTTAACAGT C-AGAGGTGA AATTCTTAGA TTTG--TTAA AGACAAACTA ATGCGAAAGC
C_hominis_KF679723       ATTTAACAGT C-AGAGGTGA AATTCTTAGA TTTG--TTAA AGACAAACTA ATGCGAAAGC
24937-C.hominis          ATTTAACAGT C-AGAGGTGA AATTCTTAGA TTTG--TTAA AGACAAACTA ATGCGAAAGC
C_parvum_KP204486        ATTTAACAGT C-AGAGGTGA AATTCTTAGA TTTG--TTAA AGACAAACTA ATGCGAAAGC
```

```
C_parvum_KM012042       ATTTAACAGT C-AGAGGTGA AATTCTTAGA TTTG--TTAA AGACAAACTA ATGCGAAAGC
C_parvum_AY268582       ATTTAACAGT C-AGAGGTGA AATTCTTAGA TTTG--TTAA AGACAAACTA ATGCGAAAGC
C_parvum_KM012045       ATTTAACAGT C-AGAGGTGA AATTCTTAGA TTTG--TTAA AGACAAACTA ATGCGAAAGC
C_parvum_KM012043       ATTTAACAGT C-AGAGGTGA AATTCTTAGA TTTG--TTAA AGACAAACTA ATGCGAAAGC
C_parvum_AF108864       ATTTAACAGT C-AGAGGTGA AATTCTTAGA TTTG--TTAA AGACAAACTA ATGCGAAAGC
C_parvum_KM085018       ATTTAACAGT C-AGAGGTGA AATTCTTAGA TTTG--TTAA AGACAAACTA ATGCGAAAGC
C_parvum_LC01201        ATTTAACAGT C-AGAGGTGA AATTCTTAGA TTTG--TTAA AGACAAACTA ATGCGAAAGC
C_parvum_KJ808688       ATTTAACAGT C-AGAGGTGA AATTCTTAGA TTTG--TTAA AGACAAACTA ATGCGAAAGC
C_parvum_LC012016       ATTTAACAGT C-AGAGGTGA AATTCTTAGA TTTG--TTAA AGACAAACTA ATGCGAAAGC
C_parvum_KM012040       ATTTAACAGT C-AGAGGTGA AATTCTTAGA TTTG--TTAA AGACAAACTA ATGCGAAAGC
C_parvum_KJ808687       ATTTAACAGT C-AGAGGTGA AATTCTTAGA TTTG--TTAA AGACAAACTA ATGCGAAAGC
C_parvum_KM012044       ATTTAACAGT C-AGAGGTGA AATTCTTAGA TTTG--TTAA AGACAAACTA ATGCGAAAGC
C_parvum_KP334136       ATTTAACAGT C-AGAGGTGA AATTCTTAGA TTTG--TTAA AGACAAACTA ATGCGAAAGC
C_parvum_KJ808689       ATTTAACAGT C-AGAGGTGA AATTCTTAGA TTTG--TTAA AGACAAACTA ATGCGAAAGC
C_parvum_KM012046       ATTTAACAGT C-AGAGGTGA AATTCTTAGA TTTG--TTAA AGACAAACTA ATGCGAAAGC
C_parvum_LC012015       ATTTAACAGT C-AGAGGTGA AATTCTTAGA TTTG--TTAA AGACAAACTA ATGCGAAAGC
C_parvum_K16X154        ATTTAACAGT C-AGAGGTGA AATTCTTAGA TTTG--TTAA AGACAAACTA ATGCGAAAGC
C_parvum_KJ808691       ATTTAACAGT C-AGAGGTGA AATTCTTAGA TTTG--TTAA AGACAAACTA ATGCGAAAGC
C_parvum_KU892559       ATTTAACAGT C-AGAGGTGA AATTCTTAGA TTTG--TTAA AGACAAACTA ATGCGAAAGC
C_parvum_AB968048       ATTTAACAGT C-AGAGGTGA AATTCTTAGA TTTG--TTAA AGACAAACTA ATGCGAAAGC
C_parvum_KJ808690       ATTTAACAGT C-AGAGGTGA AATTCTTAGA TTTG--TTAA AGACAAACTA ATGCGAAAGC
C_parvum_KJ808692       ATTTAACAGT C-AGAGGTGA AATTCTTAGA TTTG--TTAA AGACAAACTA ATGCGAAAGC
C_parvum_AF093493       ATTTAACAGT C-AGAGGTGA AATTCTTAGA TTTG--TTAA AGACAAACTA ATGCGAAAGC
C_parvum_EU553550       ATTTAACAGT C-AGAGGTGA AATTCTTAGA TTTG--TTAA AGACAAACTA ATGCGAAAGC
C_parvum_EU553557       ATTTAACAGT C-AGAGGTGA AATTCTTAGA TTTG--TTAA AGACAAACTA ATGCGAAAGC
C_parvum_KJ808693       ATTTAACAGT C-AGAGGTGA AATTCTTAGA TTTG--TTAA AGACAAACTA ATGCGAAAGC
C_parvum_KJ808694       ATTTAACAGT C-AGAGGTGA AATTCTTAGA TTTG--TTAA AGACAAACTA ATGCGAAAGC
C_parvum_KJ808695       ATTTAACAGT C-AGAGGTGA AATTCTTAGA TTTG--TTAA AGACAAACTA ATGCGAAAGC
25115-C.parvum          ATTTAACAGT C-AGAGGTGA AATTCTTAGA TTTG--TTAA AGACAAACTA ATGCGAAAGC
C_parvum_KP004204       ATTTAACAGT C-AGAGGTGA AATTCTTAGA TTTG--TTAA AGACAAACTA ATGCGAAAGC
C_parvum_KP004203       ATTTAACAGT C-AGAGGTGA AATTCTTAGA TTTG--TTAA AGACAAACTA ATGCGAAAGC
C_parvum_AB271070       ATTTAACAGT C-AGAGGTGA AATTCTTAGA TTTG--TTAA AGACAAACTA ATGCGAAAGC


                        ....|....| ....|....| ....|....| ....|....| ....|....| ....|....|
                               790        800        810        820        830        840
25330-C.ubiquitum       AT--TTGCCA ----AGGATG TTTTCATT-A ATCAAGAAC- -GAAAGTTAG GGGATCGAA-
C_ubiquitum_KC608030    AT--TTGCCA ----AGGATG TTTTCATT-A ATCAAGAAC- -GAAAGTTAG GGGATCGAA-
C_ubiquitum_KC962124    AT--TTGCCA ----AGGATG TTTTCATT-A ATCAAGAAC- -GAAAGTTAG GGGATCGAA-
C_ubiquitum_AB697056    AT--TTGCCA ----AGGATG TTTTCATT-A ATCAAGAAC- -GAAAGTTAG GGGATCGAA-
C_ubiquitum_KU531665    ---------- ---------- ---------- ---------- ---------- ----------
C_felis_JQ312664        AT--TGGCCA ----AGGATG TTTTCATT-A ATCAAGAAC- -GAAAGTTAG GGGATCGAA-
C_felis_AJ493211        AT--TTGCCA ----AGGATG TTTTCATT-A ATCAAGAAC- -GAAAGTTAG GGGATCGAA-
C_felis_JN833576        AT--TTGCCA ----AGGATG TTTTCATT-A ATCAAGAAC- -GAAAGTTAG GGGATCGAA-
C_felis_AF159113        AT--TTGCCA ----AGGATG TTTTCATT-A ATCAAGAAC- -GAAAGTTAG GGGATCGAA-
C_felis_KT749819        AT--TTGCCA ----AGGATG TTTTCATT-C ATCAAGAAC- -GAAAGTTAG GGGATCGAA-
C_felis_FJ707310        AT--TTGCCA ----AGGATG TTTTCATT-A ATCAAGAAC- -GAAAGTTAG GGGATCGAA-
C_felis_KM977642        AT--TTGCCA ----AGGATG TTTTCATT-A ATCAAGAAC- -GAAAGTTAG GGGATCGAA-
C_muris_GU319781        AT--TTGCCA ----AGGATG TTTTCATT-A ATCAAGAAC- -GAAAGTTAG GGGATCGAA-
C_muris_KF419208        AT--TTGCCA ----AGGATG TTTTCATT-A ATCAAGAAC- -GAAAGTTAG GGGATCGAA-
C_muris_EU553592        AT--TTGCCA ----AGGATG TTTTCATT-A ATCAAGAAC- -GAAAGTTAG GGGATCGAA-
C_muris_GU319783        AT--TTGCCA ----AGGATG TTTTCATT-A ATCAAGAAC- -GAAAGTTAG GGGATCGAA-
C_muris_EU245045        AT--TTGCCA ----AGGATG TTTTCATT-A ATCAAGAAC- -GAAAGTTAG GGGATCGAA-
C_muris_GQ227706        AT--TTGCCA ----AGGATG TTTTCATT-A ATCAAGAAC- -GAAAGTTAG GGGATCGAA-
C_muris_EU156446        AT--TTGCCA ----AGGATG TTTTCATT-A ATCAAGAAC- -GAAAGTTAG GGGATCGAA-
C_muris_AY642591        AT--TTGCCA ----AGGATG TTTTCATT-A ATCAAGAAC- -GAAAGTTAG GGGATCGAA-
C_muris_KY490555        AT--T-GCCA ----AGGATG TTTTCATT-A ATCAAGAAC- -GAAAGTTAG ----------
23767-C.muris           AT--TTGCCA ----AGGATG TTTTCATT-A ATCAAG---- ---------- ----------
C_andersoni_AB449819    AT--TTGCCA ----AGGATG TTTTCATT-A ATCAAGAAC- -GAAAGTTAG GGGATCGAA-
C_andersoni_EF613341    AT--TTGCCA ----AGGATG TTTTCATT-A ATCAAGAAC- -GAAAGTTAG GGGATCGAA-
C_andersoni_KF826306    AT--TTGCCA ----AGGATG TTTTCATT-A ATCAAGAAC- -GAAAGTTAG GGGATCGAA-
C_andersoni_KT922229    AT--TTGCCA ----AGGATG TTTTCATT-A ATCAAGAAC- -GAAAGTTAG GGGATCGAA-
C_andersoni_KT884487    AT--TTGCCA ----AGGATG TTTTCATT-A ATCAAGAAC- -GAAAGTTAG GGGATCGAA-
C_andersoni_LC012014    AT--TTGCCA ----AGGATG TTTTCATT-A ATCAAGAAC- -GAAAGTTAG GGGATCGAA-
C_andersoni_AB449816    AT--TTGCCA ----AGGATG TTTTCATT-A ATCAAGAAC- -GAAAGTTAG GGGATCGAA-
C_andersoni_AY954886    AT--TTGCCA ----AGGATG TTTTCATT-A ATCAAGAAC- -GAAAGTTAG GGGATCGAA-
C_andersoni_AY954885    AT--TTGCCA ----AGGATG TTTTCATT-A ATCAAGAAC- -GAAAGTTAG GGGATCGAA-
C_andersoni_AB089285    AT--TTGCCA ----AGGATG TTTTCATT-A ATCAAGAAC- -GAAAGTTAG GGGATCGAA-
C_andersoni_LC012013    AT--TTGCCA ----AGGATG TTTTCATT-A ATCAAGAAC- -GAAAGTTAG GGGATCGAA-
C_andersoni_KF826307    AT--TTGCCA ----AGGATG TTTTCATT-A ATCAAGAAC- -GAAAGTTAG GGGATCGAA-
C_andersoni_KF826305    AT--TTGCCA ----AGGATG TTTTCATT-A ATCAAGAAC- -GAAAGTTAG GGGATCGAA-
```

```
C_andersoni_KF826304    AT--TTGCCA ----AGGATG TTTTCATT-A ATCAAGAAC- -GAAAGTTAG GGGATCGAA-
C_andersoni_EU245042    AT--TTGCCA ----AGGATG TTTTCATT-A ATCAAGAAC- -GAAAGTTAG GGGATCGAA-
C_andersoni_KT922228    AT--TTGCCA ----AGGATG TTTTCATT-A ATCAAGAAC- -GAAAGTTAG GGGATCGAA-
C_andersoni_KT884488    AT--TTGCCA ----AGGATG TTTTCATT-A ATCAAGAAC- -GAAAGTTAG GGGATCGAA-
C_andersoni_AB449817    AT--TTGCCA ----AGGATG TTTTCATT-A ATCAAGAAC- -GAAAGTTAG GGGATCGAA-
C_andersoni_KT922230    AT--TTGCCA ----AGGATG TTTTCATT-A ATCAAGAAC- -GAAAGTTAG GGGATCGAA-
C_hominis_KF826315      TT--TCGCCA ----AGGATG TCTTCATT-A ATCAAGAAC- -AAAAGTTAG GGGATCGAA-
C_hominis_HQ149022      AT--TTGCCA ----AGGATG TTTTCATT-A ATCAAGAAC- -GAAAGTTAG GGGATCGAA-
C_hominis_KR296813      AT--TTGCCA ----AGGATG TTTTCATT-A ATCAAGAAC- -GAAAGTTAG GGGATCGAA-
C_hominis_AF112569      AT--TTGCCA ----AGGATG TTTTCATT-A ATCAAGAAC- -GAAAGTTAG GGGATCGAA-
C_hominis_KF679723      AT--TTGCCA ----AGGATG TTTTCATT-A ATCAAGAAC- -GAAAGTTAG GGGATCGAA-
24937-C.hominis         AT--TTGCCA ----AGGATG TTTTCATT-A ATCAAGAAC- -GAAAGTTAG GGGATCGAA-
C_parvum_KP204486       AT--TTGCCA ----AGGATG TTTTCATT-A ATCAAGAAC- -GAAAGTTAG GGGATCGAA-
C_parvum_KM012042       AT--TTGCCA ----AGGATG TTTTCATT-A ATCAAGAAC- -GAAAGTTAG GGGATCGAA-
C_parvum_AY268582       AT--TTGCCA ----AGGATG TTTTCATT-A ATCAAGAAC- -GAAAGTTAG GGGATCGAA-
C_parvum_KM012045       AT--TTGCCA ----AGGATG TTTTCATT-A ATCAAGAAC- -GAAAGTTAG GGGATCGAA-
C_parvum_KM012043       AT--TTGCCA ----AGGATG TTTTCATT-A ATCAAGAAC- -GAAAGTTAG GGGATCGAA-
C_parvum_AF108864       AT--TTGCCA ----AGGATG TTTTCATT-A ATCAAGAAC- -GAAAGTTAG GGGATCGAA-
C_parvum_KM085018       AT--TTGCCA ----AGGATG TTTTCATT-A ATCAAGAAC- -GAAAGTTAG GGGATCGAA-
C_parvum_LC01201        AT--TTGCCA ----AGGATG TTTTCATT-A ATCAAGAAC- -GAAAGTTAG GGGATCGAA-
C_parvum_KJ808688       AT--TTGCCA ----AGGATG TTTTCATT-A ATCAAGAAC- -GAAAGTTAG GGGATCGAA-
C_parvum_LC012016       AT--TTGCCA ----AGGATG TTTTCATT-A ATCAAGAAC- -GAAAGTTAG GGGATCGAA-
C_parvum_KM012040       AT--TTGCCA ----AGGATG TTTTCATT-A ATCAAGAAC- -GAAAGTTAG GGGATCGAA-
C_parvum_KJ808687       AT--TTGCCA ----AGGATG TTTTCATT-A ATCAAGAAC- -GAAAGTTAG GGGATCGAA-
C_parvum_KM012044       AT--TTGCCA ----AGGATG TTTTCATT-A ATCAAGAAC- -GAAAGTTAG GGGATCGAA-
C_parvum_KP334136       AT--TTGCCA ----AGGATG TTTTCATT-A ATCAAGAAC- -GAAAGTTAG GGGATCGAA-
C_parvum_KJ808689       AT--TTGCCA ----AGGATG TTTTCATT-A ATCAAGAAC- -GAAAGTTAG GGGATCGAA-
C_parvum_KM012046       AT--TTGCCA ----AGGATG TTTTCATT-A ATCAAGAAC- -GAAAGTTAG GGGATCGAA-
C_parvum_LC012015       AT--TTGCCA ----AGGATG TTTTCATT-A ATCAAGAAC- -GAAAGTTAG GGGATCGAA-
C_parvum_K16X154        AT--TTGCCA ----AGGATG TTTTCATT-A ATCAAGAAC- -GAAAGTTAG GGGATCGAA-
C_parvum_KJ808691       AT--TTGCCA ----AGGATG TTTTCATT-A ATCAAGAAC- -GAAAGTTAG GGGATCGAA-
C_parvum_KU892559       AT--TTGCCA ----AGGATG TTTTCATT-A ATCAAGAAC- -GAAAGTTAG GGGATCGAA-
C_parvum_AB968048       AT--TTGCCA ----AGGATG TTTTCATT-A ATCAAGAAC- -GAAAGTTAG GGGATCGAA-
C_parvum_KJ808690       AT--TTGCCA ----AGGATG TTTTCATT-A ATCAAGAAC- -GAAAGTTAG GGGATCGAA-
C_parvum_KJ808692       AT--TTGCCA ----AGGATG TTTTCATT-A ATCAAGAAC- -GAAAGTTAG GGGATCGAA-
C_parvum_AF093493       AT--TTGCCA ----AGGATG TTTTCATT-A ATCAAGAAC- -GAAAGTTAG GGGATCGAA-
C_parvum_EU553550       AT--TTGCCA ----AGGATG TTTTCATT-A ATCAAGAAC- -GAAAGTTAG GGGATCGAA-
C_parvum_EU553557       AT--TTGCCA ----AGGATG TTTTCATT-A ATCAAGAAC- -GAAAGTTAG GGGATCGAA-
C_parvum_KJ808693       AT--TTGCCA ----AGGATG TTTTCATT-A ATCAAGAAC- -GAAAGTTAG GGGATCGAA-
C_parvum_KJ808694       AT--TTGCCA ----AGGATG TTTTCATT-A ATCAAGAAC- -GAAAGTTAG GGGATCGAA-
C_parvum_KJ808695       AT--TTGCCA ----AGGATG TTTTCATT-A ATCAAGAAC- -GAAAGTTAG GGGATCGAA-
25115-C.parvum          AT--TTGCCA ----AGGATG TTTTCATT-A ATCAAGAAC- -GAAAGTTAG GGGATCGAA-
C_parvum_KP004204       AT--TTGCCA ----AGGATG TTTTCATT-A ATCAAGAAC- -GAAAGTTAG GGGATCGAA-
C_parvum_KP004203       AT--TTGCCA ----AGGATG TTTTCATT-A ATCAAGAAC- -GAAAGTTAG GGGATCGAA-
C_parvum_AB271070       AT--TTGCCA ----AGGATG TTTTCATT-A ATCAAGAAC- -GAAAGTTAG GGGATCGAA-


                        ....|....| ....|....| ....|....| ....|....| ....|....| ....|....|
                               850        860        870        880        890        900
25330-C.ubiquitum       GACGAT-CAG AT--ACCGTC GTAGTCTTAA CCATAAACTA -TGCCAACTA G--AG-----
C_ubiquitum_KC608030    GACGAT-CAG AT--ACCGTC GTAGTCTTAA CCATAAACTA -TGCCAACTA G--AGAT---
C_ubiquitum_KC962124    GACGAT-CAG AT--ACCGTC GTAGTCTTAA CCATAAACTA -TGCCGACTA G--AGAT---
C_ubiquitum_AB697056    GACGAT-CAG AT--ACCGTC GTAGTCTTAA CCATAAACTA -TGCCGACTA G--A------
C_ubiquitum_KU531665    ---------- ---------- ---------- ---------- ---------- ----------
C_felis_JQ312664        GACGAT-CAG AT--ACCGTC GTAGTCTTAA CCATAAACTA -TGCC----- ----------
C_felis_AJ493211        GACGAT-CAG AT--ACCGTC GTAGTCTTAA CCATAAACTA -TGCCAACTA G---------
C_felis_JN833576        GACGAT-CAG AT--ACCGTC GTAGTCTTAA CCATAAACTA -TGCCAAC-- ----------
C_felis_AF159113        GACGAT-CAG AT--ACCGTC GTAGTCTTAA CCATAAACTA -TGCCAACTA G--AGAT---
C_felis_KT749819        GACGAT-CAG AT--ACCGTC G--------- ---------- ---------- ----------
C_felis_FJ707310        GACGAT-CAG AT--ACCGTC GTAGTCTTAA CCATAAA--- ---------- ----------
C_felis_KM977642        GACGAT-CAG AT--ACCGTC GTAGTCTTAA CCATAAACT- ---------- ----------
C_muris_GU319781        GACGAT-CAG AT--ACCGTC GTAGTCTTAA CCATAAACTA -TGCCGACTA G--AG-----
C_muris_KF419208        GACGAT-CAG AT--ACCGTC GTAGTCTTAA CCATAAACTA -TGCCGACTA G--AGAT---
C_muris_EU553592        GACGAT-CAG AT--ACCGTC GTAGTCTTAA CCATAAACTA -TGCCGACTA G--AGAT---
C_muris_GU319783        GACGAT-CAG AT--ACCGTC GTAGTCTTAA CCATAAACTA -TGCCGACTA G--AGAT---
C_muris_EU245045        GACGAT-CAG AT--ACCGTC GTAGTCTTAA CCATAAACTA -TGCCGACTA G--AGAT---
C_muris_GQ227706        GACGAT-CAG AT--ACCGTC GTAGTCTTAA CCATAAACTA -TGCCGACTA G--AGAT---
C_muris_EU156446        GACGAT-CAG AT--ACCGTC GTAGTCTTAA CCATAAACTA -TGCCGACTA G--AGAT---
C_muris_AY642591        GACGAT-CAG AT--ACCGTC GTAGTCTTAA CCATAAACTA -TGCCGACTA G--AGAT---
C_muris_KY490555        ---------- ---------- ---------- ---------- ---------- ----------
23767-C.muris           ---------- ---------- ---------- ---------- ---------- ----------
```

```
C_andersoni_AB449819   GACGAT-CAG AT--ACCGTN NNNNCNTAAC CATAANCTAT -GCCGACTAG A--GAT----
C_andersoni_EF613341   GACGAT-CAG AT--ACCGTC GTAGTCTTAA CCATAAACTA -TGCCGACTA G--AGAT---
C_andersoni_KF826306   GACGAT-CAG AT--ACCGTC GTAGTCTTAA CCATAAACTA -TGCCGACTA G--AGAT---
C_andersoni_KT922229   GACGAT-CAG AT--ACCGTC GTAGTCTTAA CCATAAACTA -TGCCGACTA G--AGAT---
C_andersoni_KT884487   GACGAT-CAG AT--ACCGTC GTAGTCTTAA CCATAAACTA -TGCCGACTA G--AGAT---
C_andersoni_LC012014   GACGAT-CAG AT--ACCGTC GTAGTCTTAA CCATAAACTA -TGCCGACTA G--AGA----
C_andersoni_AB449816   GACGAT-CAG AT--ACCGTC GTAGTCTTAA CCATAAACTA -TGCCGACTA G--AGA----
C_andersoni_AY954886   GACGAT-CAG AT--ACCGTC GTAGTCTTAA CCATAAACTA -TGCCGACTA G--AG-----
C_andersoni_AY954885   GACGAT-CAG AT--ACCGTC GTAGTCTTAA CCATAAACTA -TGCCGACTA G--AGAT---
C_andersoni_AB089285   GACGAT-CAG AT--ACCGTC GTAGTCTTAA CCATAAACTA -TGCCGACTA G--AGAT---
C_andersoni_LC012013   GACGAT-CAG AT--ACCGTC GTAGTCTTAA CCATAAACTA -TGCCGACTA G--AGAT---
C_andersoni_KF826307   GACGAT-CAG AT--ACCGTC GTAGTCTTAA CCATAAACTA -TGCCGACTA G--AGAT---
C_andersoni_KF826305   GACGAT-CAG AT--ACCGTC GTAGTCTTAA CCATAAACTA -TGCCGACTA G--AGAT---
C_andersoni_KF826304   GACGAT-CAG AT--ACCGTC GTAGTCTTAA CCATAAACTA -TGCCGACTA G--AGAT---
C_andersoni_EU245042   GACGAT-CAG AT--ACCGTC GTAGTCTTAA CCATAAACTA -TGCCGACTA G--AGAT---
C_andersoni_KT922228   GACGAT-CAG AT--ACCGTC GTAGTCTTAA CCATAAACTA -TGCCGACTA G--AGAT---
C_andersoni_KT884488   GACGAT-CAG AT--ACCGTC GTAGTCTTAA CCATAAACTA -TGCCGACTA G--AGAT---
C_andersoni_AB449817   GACGAT-CAG AT--ACCGTC GTAGTCTTAA CCNTAAACTA -TGCCGACTA G--AGAT---
C_andersoni_KT922230   GACGAT-CAG AT--ACCGTC GTAGTCTTAA CCATAAACTA -TGCCGACTA G--AGAT---
C_hominis_KF826315     GACGAT-CAA AT--ACCGTC GTATTCTTAA CCATAAACTA -TGCCAACTA A--AGAT---
C_hominis_HQ149022     GACGAT-CAG AT--ACCGTC GTAGTCTTAA CCATAAACTA -TGCCAACTA G--AGA----
C_hominis_KR296813     GACGAT-CAG AT--ACCGTC GTAGTCTTAA CCATAAACTA -TGCCAACTA G--AGAT---
C_hominis_AF112569     GACGAT-CAG AT--ACCGTC GTAGTCTTAA CCATAAACTA -TGCCAACTA G--AGAT---
C_hominis_KF679723     GACGAT-CAG AT--ACCGTC GTAGTCTTAA CCATAAACTA -TGCCAACTA G--AGAT---
24937-C.hominis        GACGAT-CAG AT--ACCGTC GTAGTCTTAA CCATAAACTA -TGCCAACTA GA--GATTGG
C_parvum_KP204486      GACGAT-CAG AT--ACCGTC GTA------- ---------- ---------- ----------
C_parvum_KM012042      GACGAT-CAG AT--ACCGTC GTAGTCTTAA CCATAAACTA -TGCCAACTA G--AG-----
C_parvum_AY268582      GACGAT-CAG AT--ACCGTC GTAGT----- ---------- ---------- ----------
C_parvum_KM012045      GACGAT-CAG AT--ACCGTC GTAGTCTTAA CCATAAACTA -TGCCAACTA G--AGA----
C_parvum_KM012043      GACGAT-CAG AT--ACCGTC GTAGTCTTAA CCATAAACTA -TGCCAACTA G--AGAT---
C_parvum_AF108864      GACGAT-CAG AT--ACCGTC GTAGTCTTAA CCATAAACTA -TGCCAACTA G--AGAT---
C_parvum_KM085018      GACGAT-CAG AT--ACCGTC GTAGTCTTAA CCATAAACTA -TGCCAACTA G--AGAT---
C_parvum_LC01201       GACGAT-CAG AT--ACCGTC GTAGTCTTAA CCATAAACTA -TGCCAACTA G--AGAT---
C_parvum_KJ808688      GACGAT-CAG AT--ACCGTC GTAGTCTTAA CCATAAACTA -TGCCAACTA G--AGAT---
C_parvum_LC012016      GACGAT-CAG AT--ACCGTC GTAGTCTTAA CCATAAACTA -TGCCAACTA G--AGAT---
C_parvum_KM012040      GACGAT-CAG AT--ACCGTC GTAGTCTTAA CCATAAACTA -TGCCAACTA G--AGAT---
C_parvum_KJ808687      GACGAT-CAG AT--ACCGTC GTAGTCTTAA CCATAAACTA -TGCCAACTA G--AGAT---
C_parvum_KM012044      GACGAT-CAG AT--ACCGTC GTAGTCTTAA CCATAAACTA -TGCCAACTA G--AGAT---
C_parvum_KP334136      GACGAT-CAG AT--ACCGTC GTAGTCTTAA CCATAAACTA -TGCCAACTA G--AGAT---
C_parvum_KJ808689      GACGAT-CAG AT--ACCGTC GTAGTCTTAA CCATAAACTA -TGCCAACTA G--AGAT---
C_parvum_KM012046      GACGAT-CAG AT--ACCGTC GTAGTCTTAA CCATAAACTA -TGCCAACTA G--AGAT---
C_parvum_LC012015      GACGAT-CAG AT--ACCGTC GTAGTCTTAA CCATAAACTA -TGCCAACTA G--AGAT---
C_parvum_K16X154       GACGAT-CAG AT--ACCGTC GTAGTCTTAA CCATAAACTA -TGCCAACTA G--AGAT---
C_parvum_KJ808691      GACGAT-CAG AT--ACCGTC GTAGTCTTAA CCATAAACTA -TGCCAACTA G--AGAT---
C_parvum_KU892559      GACGAT-CAG AT--ACCGTC GTAGTCTTAA CCATAAACTA -TGCCAACTA G--AGAT---
C_parvum_AB968048      GACGAT-CAG AT--ACCGTC GTAGTCTTAA CCATAAACTA -TGCCAACTA G--AGAT---
C_parvum_KJ808690      GACGAT-CAG AT--ACCGTC GTAGTCTTAA CCATAAACTA -TGCCAACTA G--AGAT---
C_parvum_KJ808692      GACGAT-CAG AT--ACCGTC GTAGTCTTAA CCATAAACTA -TGCCAACTA G--AGAT---
C_parvum_AF093493      GACGAT-CAG AT--ACCGTC GTAGTCTTAA CCATAAACTA -TGCCAACTA G--AGAT---
C_parvum_EU553550      GACGAT-CAG AT--ACCGTC GTAGTCTTAA CCATAAACTA -TGCCAACTA G--AGAT---
C_parvum_EU553557      GACGAT-CAG AT--ACCGTC GTAGTCTTAA CCATAAACTA -TGCCAACTA G--AGAT---
C_parvum_KJ808693      GACGAT-CAG AT--ACCGTC GTAGTCTTAA CCATAAACTA -TGCCAACTA G--AGAT---
C_parvum_KJ808694      GACGAT-CAG AT--ACCGTC GTAGTCTTAA CCATAAACTA -TGCCAACTA G--AGAT---
C_parvum_KJ808695      GACGAT-CAG AT--ACCGTC GTAGTCTTAA CCATAAACTA -TGCCAACTA G--AGAT---
25115-C.parvum         GACGAT-CAG AT--ACCGTC GTAG------ ---------- ---------- ----------
C_parvum_KP004204      GACGAT-CAG AT--ACCGTC GTAGTCTTAA CCATAAACTA -TGCCAACTA G--AGAT---
C_parvum_KP004203      GACGAT-CAG AT--ACCGTC GTAGTCTTAA CCATAAACTA -TGCCAACTA G--AGAT---
C_parvum_AB271070      GACGAT-CAG AT--ACCGTC GTAGTCTTAA CCATAAACTA -TGCCAACTA G--AGAT---


                       ....|....| ....|....| ....|....| ....|....
                               910        920        930
25330-C.ubiquitum      ---------- ---------- ---------- ---------
C_ubiquitum_KC608030   ---------- ---------- ---------- ---------
C_ubiquitum_KC962124   ---------- ---------- ---------- ---------
C_ubiquitum_AB697056   ---------- ---------- ---------- ---------
C_ubiquitum_KU531665   ---------- ---------- ---------- ---------
C_felis_JQ312664       ---------- ---------- ---------- ---------
C_felis_AJ493211       ---------- ---------- ---------- ---------
C_felis_JN833576       ---------- ---------- ---------- ---------
C_felis_AF159113       ---------- ---------- ---------- ---------
```

```
C_felis_KT749819         ---------- ---------- ---------- ---------
C_felis_FJ707310         ---------- ---------- ---------- ---------
C_felis_KM977642         ---------- ---------- ---------- ---------
C_muris_GU319781         ---------- ---------- ---------- ---------
C_muris_KF419208         ---------- ---------- ---------- ---------
C_muris_EU553592         ---------- ---------- ---------- ---------
C_muris_GU319783         ---------- ---------- ---------- ---------
C_muris_EU245045         ---------- ---------- ---------- ---------
C_muris_GQ227706         ---------- ---------- ---------- ---------
C_muris_EU156446         ---------- ---------- ---------- ---------
C_muris_AY642591         ---------- ---------- ---------- ---------
C_muris_KY490555         ---------- ---------- ---------- ---------
23767-C.muris            ---------- ---------- ---------- ---------
C_andersoni_AB449819     ---------- ---------- ---------- ---------
C_andersoni_EF613341     ---------- ---------- ---------- ---------
C_andersoni_KF826306     ---------- ---------- ---------- ---------
C_andersoni_KT922229     ---------- ---------- ---------- ---------
C_andersoni_KT884487     ---------- ---------- ---------- ---------
C_andersoni_LC012014     ---------- ---------- ---------- ---------
C_andersoni_AB449816     ---------- ---------- ---------- ---------
C_andersoni_AY954886     ---------- ---------- ---------- ---------
C_andersoni_AY954885     ---------- ---------- ---------- ---------
C_andersoni_AB089285     ---------- ---------- ---------- ---------
C_andersoni_LC012013     ---------- ---------- ---------- ---------
C_andersoni_KF826307     ---------- ---------- ---------- ---------
C_andersoni_KF826305     ---------- ---------- ---------- ---------
C_andersoni_KF826304     ---------- ---------- ---------- ---------
C_andersoni_EU245042     ---------- ---------- ---------- ---------
C_andersoni_KT922228     ---------- ---------- ---------- ---------
C_andersoni_KT884488     ---------- ---------- ---------- ---------
C_andersoni_AB449817     ---------- ---------- ---------- ---------
C_andersoni_KT922230     ---------- ---------- ---------- ---------
C_hominis_KF826315       ---------- ---------- ---------- ---------
C_hominis_HQ149022       ---------- ---------- ---------- ---------
C_hominis_KR296813       ---------- ---------- ---------- ---------
C_hominis_AF112569       ---------- ---------- ---------- ---------
C_hominis_KF679723       ---------- ---------- ---------- ---------
24937-C.hominis          AGGTTGTTCC TTACTCCTTC ---------- ---------
C_parvum_KP204486        ---------- ---------- ---------- ---------
C_parvum_KM012042        ---------- ---------- ---------- ---------
C_parvum_AY268582        ---------- ---------- ---------- ---------
C_parvum_KM012045        ---------- ---------- ---------- ---------
C_parvum_KM012043        ---------- ---------- ---------- ---------
C_parvum_AF108864        ---------- ---------- ---------- ---------
C_parvum_KM085018        ---------- ---------- ---------- ---------
C_parvum_LC01201         ---------- ---------- ---------- ---------
C_parvum_KJ808688        ---------- ---------- ---------- ---------
C_parvum_LC012016        ---------- ---------- ---------- ---------
C_parvum_KM012040        ---------- ---------- ---------- ---------
C_parvum_KJ808687        ---------- ---------- ---------- ---------
C_parvum_KM012044        ---------- ---------- ---------- ---------
C_parvum_KP334136        ---------- ---------- ---------- ---------
C_parvum_KJ808689        ---------- ---------- ---------- ---------
C_parvum_KM012046        ---------- ---------- ---------- ---------
C_parvum_LC012015        ---------- ---------- ---------- ---------
C_parvum_K16X154         ---------- ---------- ---------- ---------
C_parvum_KJ808691        ---------- ---------- ---------- ---------
C_parvum_KU892559        ---------- ---------- ---------- ---------
C_parvum_AB968048        ---------- ---------- ---------- ---------
C_parvum_KJ808690        ---------- ---------- ---------- ---------
C_parvum_KJ808692        ---------- ---------- ---------- ---------
C_parvum_AF093493        ---------- ---------- ---------- ---------
C_parvum_EU553550        ---------- ---------- ---------- ---------
C_parvum_EU553557        ---------- ---------- ---------- ---------
C_parvum_KJ808693        ---------- ---------- ---------- ---------
C_parvum_KJ808694        ---------- ---------- ---------- ---------
C_parvum_KJ808695        ---------- ---------- ---------- ---------
25115-C.parvum           ---------- ---------- ---------- ---------
C_parvum_KP004204        ---------- ---------- ---------- ---------
C_parvum_KP004203        ---------- ---------- ---------- ---------
C_parvum_AB271070        ---------- ---------- ---------- ---------
```
