# Supplementary material for: Cryptosporidium parvum and Cryptosporidium hominis subtypes in crab-eating macaques
Source: Parasit Vectors. 2019 Jul 15;12:350. doi: 10.1186/s13071-019-3604-7 (PMC6631616; doi:10.1186/s13071-019-3604-7)
Supplement: Supplementary file 2 — Additional file 2: Figure S2. Alignment of nucleotide sequences of the 60 kDa glycoprotein gene of Cryptosporidium parvum and Cryptosporidium hominis. [file 13071_2019_3604_MOESM2_ESM.pdf]

Additional file 2: Figure S2. Alignment of nucleotide sequences of the 60 kDa glycoprotein gene of *Cryptosporidium parvum* and *Cryptosporidium hominis*.

```
                               ....|....| ....|....| ....|....| ....|....| ....|....| ....|....|
                                       10         20         30         40         50         60
IiA17_KF679724_                -GTTTCTGTT GAGGGC---- ---------- -TCATCATCA TCATCATCAT CATCATCATC
25132-IiA17                    TGTTTCTGTT GAGGGC---- ---------- -TCATCATCA TCATCATCAT CATCATCATC
IIoA13G1_KC885906              TGTTTCTGTT GAGGGC---- ---------- ---------- TCATCATCAT CATCATCATC
25093-IIoA14G1                 TGTTTCTGTT GAGGGC---- ---------- -------TCA TCATCATCAT CATCATCATC
IIpA9_KC885904_                TGTTTCTGTT GAGGGC---- ---------- ---------- ------TCAT CATCA-----
IInA8_FJ897787                 TGTTCCTGTT GAGGGC---- ---------- ---------- ------TCAT CA--------
IIdA19G1_KJ802724              TGTTTCTGTT GAGGGT---- ---------- -TCATCATCA TCATCATCAT CATCATCATC
25883-IIdA19G1                 TGTTTCTGTT GAGGGT---- ---------- -TCATCATCA TCATCATCAT CATCATCATC
IIbA14_AF402285                TGTTTCTGTT GAGGGC---- ---------- ---------- ---TCATCAT CATCATCATC
IIkA14_AB237137                TGTTCCTGTT GAGGGC---- ---------- ---------- TCATCATCAT CATCATCATC
IIaA15G2R1_JF727769            TGTTCCTGTT GAGGGC---- ---------- -TCATCATCG TCATCGTCAT CATCATCATC
26239-In                       ---------- ---------- ---------- ---------- ---------- ----------
IoA15_KX926458                 ---------- ----GC---- ---------- ---------A TCATCATCAT CATCATCATC
24965-InA14                    TGTTTCTGTT GAGGGC---- ---------- ---------- TCATCATCAT CATCATCATC
26450-InA17                    TGTTTCTGTT GAGGGC---- ---------- -TCATCATCA TCATCATCAT CATCATCATC
26114-InA26                    TGTTTCTGTT GAGGGC---T CATCATCATC ATCATCATCA TCATCATCAT CATCATCATC
IdA22_GU214353                 TGTTTCTGTT GAGGGC---- ---------- -TCATCATCA TCATCATCAT CATCATCATC
IhA14G1_FJ971716_              TGTTTCTGTT GAGGGC---- ---------- -------TCA TCATCATCGT CATCATCATC
IjA14_JF681174                 TGTTTCTGTT GAGGGC---- ---------- ---------- TCATCATCAT CATCATCATC
IaA23R4_AF164502               TGTTTCTGTT GAGGGA---- --------TC ATCATCATCA TCATCATCAT CATCATCATC
24972-ImA18                    ---------- ---GGC---- ---------- -TCATCATCA TCATCATCAT CATCATCATC
IgA24_EF208067                 TGTTTCTGTT GAGGGC---- ---------- -TCATCATCA TCATCATCAT CATCATCATC
IfA19G1R5_AF440638             TGTTTCTGTT GAGAGC---- ---------- -TCATCATCA TCATCATCAT CATCATCATC
IIcA5G3b_AF164501              TGTTTCTGTT GAGAGC---- ---------- ---------- ------TCAT CG--------
IeA11G3T3_DQ665689             TGTTTCTGTT GAGGGC---- ---------- ----TCATCA TCATCTTCAT CATCGTCTTC
IlA7G4_KP314263_               TGTTCCTGTT GGGGGC---- ---------- ---------- ------TCAT CGTCATCA--
C.meleagridis_AB539719         ---------T GATAGCTCAT CGTCATCATC ATCATCATCA TCATCATCAT CATCATCATC
IbA10G2_AY262031               TGTTTCTGTT GAGAGC---- ---------- ---------- ------TCAT CATCATCATC
IIeA12G1_AY382675              TGCTTCTGTT GAGGGC---- ---------- ---------- ------TCAT CATCATCATC
IkA15G1_KJ941148               TGTTTCTGTT GAGGGC---- ---------- ----TCATCA TCATCATCAT CATCATCATC


                               ....|....| ....|....| ....|....| ....|....| ....|....| ....|....|
                                       70         80         90        100        110        120
IiA17_KF679724_                ATCA------ ---------T CATCATCATC ATCATCA--- ----ACAACA ACCGTTGCAC
25132-IiA17                    ATCA------ ---------T CATCATCATC ATCATCA--- ----ACAACA ACCGTTGCAC
IIoA13G1_KC885906              ATCA------ ---------T CATCATCATC GTCATCA--- ----ACATCG ACTGTAGCAC
25093-IIoA14G1                 ATCA------ ---------T CATCATCATC GTCATCA--- ----ACATCG ACTGTAGCAC
IIpA9_KC885904_                ---------- ---------T CATCATCATC ATCATCA--- ----ACATCG ACTGTAGCAC
IInA8_FJ897787                 ---------- ---------T CATCATCATC ATCATCA--- ----ACATCG ACTGTAGCAC
IIdA19G1_KJ802724              ATCATCATCA TCG------T CATCATCATC ATCATCA--- ----ACATCG ACTGTAGCAC
25883-IIdA19G1                 ATCATCATCA TCG------T CATCATCATC ATCATCA--- ----ACATCG ACTGTAGCAC
IIbA14_AF402285                ATCA------ ---------T CATCATCATC ATCATCA--- ----TCAACG ACCGTCGCAC
IIkA14_AB237137                ATCA------ ---------T CATCATCATC ATCATCA--- ----ACATCA ACCGTAGCAC
IIaA15G2R1_JF727769            ATCA------ ---------T CATCATCATC ATCATCA--- ----ACATCA ACCGTCGCAC
26239-In                       ---------- ---------- ---------- ---------- ---------- ----------
IoA15_KX926458                 ATCA------ ---------T CATCATCATC ATCATCA--- ----TCAACG ACCGTCGCAC
24965-InA14                    ATCA------ ---------T CATCATCATC ATCATCA--- ----ACGACG ACCGTCGCAC
26450-InA17                    ATCA------ ---------T CATCATCATC ATCATCA--- ----ACGACG ACCGTCGCAC
26114-InA26                    ATCATCATCA TCATCATCAT CATCATCATC ATCATCA--- ----ACGACG ACCGTCGCAC
IdA22_GU214353                 ATCATCATCA TCATCA---T CATCATCATC ATCATCA--- ----TCAACG ACCGTCGCAC
IhA14G1_FJ971716_              ATCA------ ---------T CATCATCATC ATCATCA--- ----ACATCG ACCGTCGCAC
IjA14_JF681174                 ATCA------ ---------T CATCATCATC ATCATCA--- ----ACGTCA ACCGCCGCAC
IaA23R4_AF164502               ATCATCATCA TCATCATCAT CATCATCATC ATCATCA--- ----ACATCG ACCGTCGCAC
24972-ImA18                    ATCATCA--- ---------T CATCATCATC ATCATCA--- ----ACGACG ACCGTCGCAC
IgA24_EF208067                 ATCATCATCA TCATCATCAT CATCATCATC ATCATCATCA TCAGACGACG ACCGTCGCAC
IfA19G1R5_AF440638             ATCATCATCA TCA------T CATCATCATC ATCGTCA--- ----ACAACA ACCCCCGCAC
IIcA5G3b_AF164501              ---------- ---------T CATCATCGTC ATCGTCA--- ----ACAACA ACCCCCGCAC
IeA11G3T3_DQ665689             ATCT------ ---------T CATCATCATC ATCGTCG--- ----TCAACA ACCCCAGCAC
IlA7G4_KP314263_               ---------- ---------T CATCATCGTC GTCATCG--- ----TCAACA ACCCCCGCAC
C.meleagridis_AB539719         ATCATCA--- ---------T CATCATCATC ATCATCA--- ----ACATCA ACCGCCGCAC
IbA10G2_AY262031               ATCA------ ---------T CATCGTCATC ATCGTCA--- ----ACAACA ACCCCCGCAC
IIeA12G1_AY382675              ATCA------ ---------T CATCATCATC ATCATCA--- ----TCGACC ACCGTCGCAC
```

```
IkA15G1_KJ941148           ATCA------ ---------T CATCATCGTC ATCATCA--- ----ACAACA ACCGTTGCAC

                           ....|....| ....|....| ....|....| ....|....| ....|....| ....|....|
                                  130        140        150        160        170        180
IiA17_KF679724_            CAGTTTCAAA TAAGGCAAGA AATGCAGATG ---------- ---------- ----------
25132-IiA17                CAGTTTCAAA TAAGGCAAGA AATGCAGATG ---------- ---------- ----------
IIoA13G1_KC885906          CAACTCCAAA GAAAGAAAGA ACTGGAGAGG ---------- ---------- ----------
25093-IIoA14G1             CAACTCCAAA GAAAGAAAGA ACTGGAGAGG ---------- ---------- ----------
IIpA9_KC885904_            CAACTCCAAA GAAAGAAAGA ACTGGAGAGG ---------- ---------- ----------
IInA8_FJ897787             CAACTCCAAA GAAAGAAAGA ACTGGAGAGG ---------- ---------- ----------
IIdA19G1_KJ802724          CAACTCCAAA GAAAGAAAGA ACTGGAGAGG ---------- ---------- ----------
25883-IIdA19G1             CAACTCCAAA GAAAGAAAGA ACTGGAGAGG ---------- ---------- ----------
IIbA14_AF402285            CAGCTTCAAA TAAGGCAAGA ACTGGAGAAG ---------- ---------- ----------
IIkA14_AB237137            CAG---CAAA TAAGGCAAGA ACTGGAGAAG ---------- ---------- ----------
IIaA15G2R1_JF727769        CAG---CAAA TAAGGCAAGA ACTGGAGAAG ---------- ---------- ----------
26239-In                   -AGCTTCAAA TAAGGCAAGA ACTGGAGAAG ACACAGGACG AAGCGGAGGA AGTCGAGGTT
IoA15_KX926458             CAGCTTCAAA TAAGGCAAGA ACTGGAGAAG ACACAGGACG AAGCGAAGGA AGTCAAGGTT
24965-InA14                CAGCTTCAAA TAAGGCAAGA ACTGGAGAAG ACACAGGACG AAGCGGAGGA AGTCGAGGTT
26450-InA17                CAGCTTCAAA TAAGGCAAGA ACTGGAGAAG ACACAGGACG AAGCGGAGGA AGTCGAGGTT
26114-InA26                CAGCTTCAAA TAAGGCAAGA ACTGGAGAAG ACACAGGACG AAGCGGAGGA AGTCGAGGTT
IdA22_GU214353             CAGCTTCAAA TAAGGCAAGA ACTGGAGAGG ACACAGGACG AAGCGAAGGA AGTCAAGGTT
IhA14G1_FJ971716_          CAGCTCCAAA GAAAGAAAGA ACTGTAGAGG ---------- ---------- ----------
IjA14_JF681174             CAGCTCCAAA GAAGGCAAGA AATGTAGAAG ---------- ---------- ----------
IaA23R4_AF164502           CAGCTCCAAA GAAAGAAAGA ACTGTAGAGG ---------- ---------- ----------
24972-ImA18                CAGCTCCAAA GAAAGAAAGA ACTGTAGAGG ---------- ---------- ----------
IgA24_EF208067             CAGCTCCAAA GAAAGAAAGA ACTGTAGATG ---------- ---------- ----------
IfA19G1R5_AF440638         CAGCTCCAAA GAAGGCAAGA GAAGCAGAAG ---------- ---------- ----------
IIcA5G3b_AF164501          CAGCTCCAAA GAAGGTAAGA GAAAGCGAAG ---------- ---------- ----------
IeA11G3T3_DQ665689         CAGCTTCAAA GAAGGTAAGA GAAGCAGAAG GC-------- ---------- ----------
IlA7G4_KP314263_           CAGCTCCAAA GAAGGCAAGA GAAGCAGAAG ---------- ---------- ----------
C.meleagridis_AB539719     CAACC----- ---------- ACTGAAGATG ---------- ---------- ----------
IbA10G2_AY262031           CAGCTCCAAA GAAGGCAAGA GAAGCAGATG ---------- ---------- ----------
IIeA12G1_AY382675          CAGCTCCAAA GAAAGAAAGA ACTGGAGAGG ---------- ---------- ----------
IkA15G1_KJ941148           CAGCTTCAAA TAAGGCAAGA AATGCAGAGG ---------- ---------- ----------

                           ....|....| ....|....| ....|....| ....|....| ....|....| ....|....|
                                  190        200        210        220        230        240
IiA17_KF679724_            ---------- ---------- ---------- ---------- ---------- ----------
25132-IiA17                ---------- ---------- ---------- ---------- ---------- ----------
IIoA13G1_KC885906          ---------- ---------- ---------- ---------- ---------- ----------
25093-IIoA14G1             ---------- ---------- ---------- ---------- ---------- ----------
IIpA9_KC885904_            ---------- ---------- ---------- ---------- ---------- ----------
IInA8_FJ897787             ---------- ---------- ---------- ---------- ---------- ----------
IIdA19G1_KJ802724          ---------- ---------- ---------- ---------- ---------- ----------
25883-IIdA19G1             ---------- ---------- ---------- ---------- ---------- ----------
IIbA14_AF402285            ---------- ---------- ---------- ---------- ---------- ----------
IIkA14_AB237137            ---------- ---------- ---------- ---------- ---------- ----------
IIaA15G2R1_JF727769        ---------- ---------- ---------- ---------- ---------- ----------
26239-In                   CTGAAGAACA CGAAGACGGA GAGGACGATA GTTCAGATCC TAGTGGAGAC AGTGTAGGAG
IoA15_KX926458             CTGAAGAACA CCAAGACGGA AAGGACGATA GTTCAGATCC TAGTGGAGAC AGTGTAGGAA
24965-InA14                CTGAAGAACA CGAAGACGGA GAGGACGATA GTTCAGATCC TAGTGGAGAC AGTGTAGGAG
26450-InA17                CTGAAGAACA CGAAGACGGA GAGGACGATA GTTCAGATCC TAGTGGAGAC AGTGTAGGAG
26114-InA26                CTGAAGAACA CGAAGACGGA GAGGACGATA GTTCAGATCC TAGTGGAGAC AGTGTAGGAG
IdA22_GU214353             CTGAAGAACA CCAAGACGGA GAGGACGATA GTTCAGATTC TAGTGGAGGC AGTGTAGGAG
IhA14G1_FJ971716_          ---------- ---------- ---------- ---------- ---------- ----------
IjA14_JF681174             ---------- ---------- ---------- ---------- ---------- ----------
IaA23R4_AF164502           ---------- ---------- ---------- ---------- ---------- ----------
24972-ImA18                ---------- ---------- ---------- ---------- ---------- ----------
IgA24_EF208067             ---------- ---------- ---------- ---------- ---------- ----------
IfA19G1R5_AF440638         ---------- ---------- ---------- ---------- ---------- ----------
IIcA5G3b_AF164501          ---------- ---------- ---------- ---------- ---------- ----------
IeA11G3T3_DQ665689         ---------- ---------- ---------- ---------- ---------- ----------
IlA7G4_KP314263_           ---------- ---------- ---------- ---------- ---------- ----------
C.meleagridis_AB539719     ---------- ---------- ---------- ---------- ---------- ----------
IbA10G2_AY262031           ---------- ---------- ---------- ---------- ---------- ----------
IIeA12G1_AY382675          ---------- ---------- ---------- ---------- ---------- ----------
IkA15G1_KJ941148           ---------- ---------- ---------- ---------- ---------- ----------
```

```
                          ....|....| ....|....| ....|....| ....|....| ....|....| ....|....|
                                 250        260        270        280        290        300
IiA17_KF679724_           ---------- --------AA GAAGGAAAGA ACGAAGAAAG ---------- ----------
25132-IiA17               ---------- --------AA GAAGGAAAGA ACGAAGAAAG ---------- ----------
IIoA13G1_KC885906         ---------- ---------- ---------- ---AAGCAGA ---------- ----------
25093-IIoA14G1            ---------- ---------- ---------- ---AAGCAGA ---------- ----------
IIpA9_KC885904_           ---------- ---------- ---------- ---AAGCAGG ---------- ----------
IInA8_FJ897787            ---------- ---------- ---------- ---AACCAGG ---------- ----------
IIdA19G1_KJ802724         ---------- ---------- ---------- ---AAGTAGG ---------- ----------
25883-IIdA19G1            ---------- ---------- ---------- ---AAGTAGG ---------- ----------
IIbA14_AF402285           ---------- ---------- ---------- ACACAGAAGG ---------- ----------
IIkA14_AB237137           ---------- ---------- ---------- -ATCAGAAGA ---------- ----------
IIaA15G2R1_JF727769       ---------- ---------- ---------- ACGCAGAAGG ---------- ----------
26239-In                  GCACAGAGAG CGGAAGTGCA GGAGGAAAGA ACGAAGAAGA ---------- ----------
IoA15_KX926458            GCACAGAGAG CGGAAGTGCA GGAGGAAAGA ACGAAGGAGA ---------- ----------
24965-InA14               GCACAGAGAG CGGAAGTGCA GGAGGAAAGA ACGAAGAAGA ---------- ----------
26450-InA17               GCACAGAGAG CGGAAGTGCA GGAGGAAAGA ACGAAGAAGA ---------- ----------
26114-InA26               GCACAGAGAG CGGAAGTGCA GGAGGAAAGA ACGAAGAAGA ---------- ----------
IdA22_GU214353            GCACAGAGAG CGGAAGTGCA GGAGGAAAGA ACGAAGAAGA ---------- ----------
IhA14G1_FJ971716_         ---------- -----GCGCG GGAGAAGAGA ACGAAGAAAG ---------- ----------
IjA14_JF681174            ---------- --------GA CAAGGACAAG AAGAACAAGG ---------A CAAGAACAAG
IaA23R4_AF164502          ---------- --GCGGCACG GAAGGAAAGA ACGAAGAAAG ---------- ----------
24972-ImA18               ---------- -----GCACG GAAGGAAAGA ACGAAGAAAG ---------- ----------
IgA24_EF208067            ---------- -----GCGCA GAAGAAAAGA ACGAAGTAAG ---------- ----------
IfA19G1R5_AF440638        ---------- -----GCAAA GAAGCAGAAG GCAAAGAAGA AGAGGGCAAA GAAGAAGAGG
IIcA5G3b_AF164501         ---------- ---------- -AAGGGAAGA ACAGTGAAGA ---------- ----------
IeA11G3T3_DQ665689        ---------- ----AGTGAA GAAAAGGACA GCGAAGAAAA GGACAGTGAA GAAAAGGGCA
IlA7G4_KP314263_          ---------- --------GC GGAGAAAAGA ACAATGAAGA ---------- ----------
C.meleagridis_AB539719    ---------- ---------- ---------- ACCAGGATAG ---------- ----------
IbA10G2_AY262031          ---------- -----GCGGA GAAGAAAAGA ACAATGAAGA ---------- ----------
IIeA12G1_AY382675         ---------- -----GCGTA GATGGAAAGG ACCAAGTAGA ---------- ----------
IkA15G1_KJ941148          ---------- --------AA GAAGGAAAGA TCGATGAAGG ---------- ----------


                          ....|....| ....|....| ....|....| ....|....| ....|....| ....|....|
                                 310        320        330        340        350        360
IiA17_KF679724_           ---------- CAGTCAAAGT CCTAGTGTTC ---CTGGATC ---T------ ---------T
25132-IiA17               ---------- CAGTCAAAGT CCTAGTGTTC ---CTGGATC ---T------ ---------T
IIoA13G1_KC885906         ---------- TAGTCAAGGT T--------- ---CTGAAGA ---T------ ---------C
25093-IIoA14G1            ---------- TAGTCAAGGT T--------- ---CTGAAGA ---T------ ---------C
IIpA9_KC885904_           ---------- TAGTCAAGGT T--------- ---CTGAAGA ---T------ ---------C
IInA8_FJ897787            ---------- TAGTCCAAGT T--------- ---CTGAAGG ---T------ ---------C
IIdA19G1_KJ802724         ---------- TAATCCAGGT T--------- ---CTGAAGG ---T------ ---------C
25883-IIdA19G1            ---------- TAATCCAGGT T--------- ---CTGAAGG ---T------ ---------C
IIbA14_AF402285           ---------- TAGTCAAGTT TCTGGTGGTA CTCCTGAATC ---T------ ---------T
IIkA14_AB237137           ---------- CAGTCAAGAT ACTAGCA--- ---CTGAAGC ---T------ ----------
IIaA15G2R1_JF727769       ---------- CAGTCAAGAT TCTAGTGGTA ---CTGAAGC ---T------ ---------T
26239-In                  ---------- TAGTTCAAGT T--------- ---CTGGAGG TGTT------ ---------C
IoA15_KX926458            ---------- TAGTTCAAGT T--------- ---CTGGAGG TGTT------ ---------C
24965-InA14               ---------- TAGTTCAAGT T--------- ---CTGGAGG TGTT------ ---------C
26450-InA17               ---------- TAGTTCAAGT T--------- ---CTGGAGG TGTT------ ---------C
26114-InA26               ---------- TAGTTCAAGT T--------- ---CTGGAGG TGTT------ ---------C
IdA22_GU214353            ---------- TAGTTCAAGT T--------- ---CTGGAGG TGCT------ ---------C
IhA14G1_FJ971716_         ---------- CAGTCCAGGT T--------- ---CTGGAGA TCAT------ ---------C
IjA14_JF681174            AAGAACAAGA AGAACAAGAT TCTACTGGTA ---CTGAGGC ---T------ ---------T
IaA23R4_AF164502          ---------- CAGTCCAGGT T--------- ---CTGAAGA ---A------ ---------C
24972-ImA18               ---------- CAGTCCAGGT T--------- ---CTGAAGA ---A------ ---------C
IgA24_EF208067            ---------- TAGTCCAGGT T--------- ---CTGAAGA ---A------ ---------C
IfA19G1R5_AF440638        GCAGTGAAGA AAGCCAAGGT CCCACTAGTT ---CTGGAAG ---T------ ---------G
IIcA5G3b_AF164501         ---------- TAGTCAAACT C--------- ---CCGCTAG ---T------ ---------C
IeA11G3T3_DQ665689        GTGAAGAATG TATCCAAACT C--------- ---CCGCTAG ---T------ ---------C
IlA7G4_KP314263_          ---------- AAGCCCAGCT CCCGCTGATC ---CTGGGAG TGGTGGGGGG AATGGAGGAC
C.meleagridis_AB539719    ---------- TACTCAAAGT A--------- ---------- ---------- ----------
IbA10G2_AY262031          ---------- AAGCCAAACT CCCGCTAGTC ---CTGGAAG ---T------ ----------
IIeA12G1_AY382675         ---------- TAGTGCAGGT T--------- ---CTGA--- ---T------ ---------C
IkA15G1_KJ941148          ---------- CAGTCAAAGT CCTAGTGATG TTCCTGGATC ---T------ ---------T


                          ....|....| ....|....| ....|....| ....|....| ....|....| ....|....|
                                 370        380        390        400        410        420
IiA17_KF679724_           CTGACAGT-- ---------- ---------- ---------- -------CAA GACACT----
```

```
25132-IiA17                   CTGACAGT-- ---------- ---------- ---------- -------CAA GACACT----
IIoA13G1_KC885906             AGGACGGT-- ---------- ---------- ---------- ----AAAGGA GACACT----
25093-IIoA14G1                AGGACGGT-- ---------- ---------- ---------- ----AAAGGA GACACT----
IIpA9_KC885904_               AGGTCGGT-- ---------- ---------- ---------- ----AAAGGA GACACT----
IInA8_FJ897787                AGGACGGT-- ---------- ---------- ---------- ----AAAGAA GACGCT----
IIdA19G1_KJ802724             AGGACGGT-- ---------- ---------- ---------- ----AAAGGA GACACT----
25883-IIdA19G1                AGGACGGT-- ---------- ---------- ---------- ----AAAGGA GACACT----
IIbA14_AF402285               CTGGCAGC-- ---------- ---------- ---------- -------CAG GACACT----
IIkA14_AB237137               ---------- ---------- ---------- ---------- ---------- ----------
IIaA15G2R1_JF727769           CTGGTAGC-- ---------- ---------- ---------- -------CAG GGTTCT----
26239-In                      AGGATGGC-- ---------- ---------- ---------- ----ATTGGA GGCACT----
IoA15_KX926458                AGGATGGC-- ---------- ---------- ---------- ----AGTGGA GGCACT----
24965-InA14                   AGGATGGC-- ---------- ---------- ---------- ----ATTGGA GGCACT----
26450-InA17                   AGGATGGC-- ---------- ---------- ---------- ----ATTGGA GGCACT----
26114-InA26                   AGGATGGC-- ---------- ---------- ---------- ----ATTGGA GGCACT----
IdA22_GU214353                AGGACGGC-- ---------- ---------- ---------- ----AGTGGA GGCACT----
IhA14G1_FJ971716_             AAGAGGGC-- ---------- ---------- ---------- ----AAGGAA GAAGATGTAG
IjA14_JF681174                CTGGTGGC-- ---------- ---------- ---------- -------CAG GATGCTTCTT
IaA23R4_AF164502              AAGACGGT-- -GGTAAGGAA GACGGTGGTA AGGAAGACGG TGGTAAGGAA AACGGT----
24972-ImA18                   AAGACGGT-- ---------- ------GGTA AGGAAGACGG TGGTAAGGAA GACGCT----
IgA24_EF208067                AAGACAGT-- ---------- ---------- ---------- ----AAAGAA AACACT----
IfA19G1R5_AF440638            GAGTGGGG-- --------AG TGAAGGAATG ATCAAGGTGA CTCTAAAGGA GAC-------
IIcA5G3b_AF164501             CTGGAAGT-- ---------- ------GATT CTCAGGATAG CTCTAAAGGA GAC-------
IeA11G3T3_DQ665689            CTGGACGTGG AGGGGTGAGT GAAGGAGATA CTCAAGGTGA CTCTAAAGGA GAC-------
IlA7G4_KP314263_              AAAATACTGA AGGTGGCTCC ACAGGAGATG AACAAGGTGG CTCCACAGGA GACTCT----
C.meleagridis_AB539719        ---------- ---------- ---------- ---------- ---------- ----------
IbA10G2_AY262031              -----GGTGG GGTGAGTGAA GGACAAGATA CTCAAGGTGG CTCCAAAGGA GACGCT----
IIeA12G1_AY382675             AGAGCAGT-- ---------- ---------- ---------- ----AAAGGA GACACT----
IkA15G1_KJ941148              CTGACAGT-- ---------- ---------- ---------- -------CAA GACACT----

                              ....|....| ....|....| ....|....| ....|....| ....|....| ....|....|
                                     430        440        450        460        470        480
IiA17_KF679724_               --------GA AGAAAGTGAG GACAGTACTC AATCTAGTGC ------TGCT GATTCCCAAA
25132-IiA17                   --------GA AGAAAGTGAG GACAGTACTC AATCTAGTGC ------TGCT GATTCCCAAA
IIoA13G1_KC885906             ------GTAG AAGAGCAGGA GACAGTG--C AGACCGAGAG ------TACT GTTTCTCAA-
25093-IIoA14G1                ------GTAG AAGAGCAGGA GACAGTG--C AGACCGAGAG ------TACT GTTTCTCAA-
IIpA9_KC885904_               -----GTAGA TGGAGCAGGA GACGATGAAC AGACCGAGAG ------TACT GTTTCTCAA-
IInA8_FJ897787                --------GA AGAAACAGAA GACGGTAAAC AGACCGAGAG ------TACT GTTACTCAA-
IIdA19G1_KJ802724             --------GA AGAAACAGAA GACAAT---C AGACCGAGAG ------TACT GTTTCTCAA-
25883-IIdA19G1                --------GA AGAAACAGAA GACAAT---C AGACCGAGAG ------TACT GTTTCTCAA-
IIbA14_AF402285               ---------G AGAAAGTGAA GACGGTTCCC AAACTAG--- ------TACT GTCTCCGAAT
IIkA14_AB237137               ---------- -GGTAGCGAA GATGGTACTC AAACTAG--- ------TGCT GCTTCCGAAC
IIaA15G2R1_JF727769           --GAAGAGGA AGGTAGTGAA GACGATGGCC AAACTAG--- ------TGCT GCTTCCCAAC
26239-In                      ---------- ----GCAGAA GGCGCTACTC AGTCCGAGGC ------TACT GCTTCTCAA-
IoA15_KX926458                ---------- ----GCAGAA GGCGCTACTC AGTCCGAGGC ------TACT GCTTCTCAA-
24965-InA14                   ---------- ----GCAGAA GGCGCTACTC AGTCCGAGGC ------TACT GCTTCTCAA-
26450-InA17                   ---------- ----GCAGAA GGCGCTACTC AGTCCGAGGC ------TACT GCTTCTCAA-
26114-InA26                   ---------- ----GCAGAA GGCGCTACTC AGTCCGAGGC ------TACT GCTTCTCAA-
IdA22_GU214353                ---------- ----GCAGAA GGCGCTACTC AGTCCGAGGC ------TACT GCTTCTCAA-
IhA14G1_FJ971716_             AAGAAGAAGA GGGCACAGGC GACGGGAAAC AGACCGAGAG ------TGCT GGTTCTCAA-
IjA14_JF681174                CTAAAGAGGA AGGTAGTGAA AACACTACTC AAACTAG--- ------TGAT AGTTCCGACT
IaA23R4_AF164502              -----GAAGG AGACACAGTA GACGGGGAAC AAACCGGGAG ---------T GGTTCTCAA-
24972-ImA18                   --GAAGAAGG AGACACAGTA GACGGGGAAC AAACCGGGAG ---------T GGTTCTCAA-
IgA24_EF208067                -----GAAGG AGACACAGCA GACGAGGAAG AAGCCGGGAG ---------T AGTTCTCAG-
IfA19G1R5_AF440638            --------GG AGCTAGTGAA GATGATAATA AAAATCAAGA TGGTGACACT TCTTCCGAAT
IIcA5G3b_AF164501             --------GA AG---CTGTA GATGG----- ---------- ------AGCC GCTTCCGGAT
IeA11G3T3_DQ665689            --------GG AGTTAGTTCA GATGAGAACC AAAGTCAAGG TGGGGACGCT ACTCCCGGAT
IlA7G4_KP314263_              -----GTGGA TGACACTGAA GACGATGAAC AAGCCGATGA ------GAGT GCTAGCCAAC
C.meleagridis_AB539719        ---------- -----CTGGT GACAGCAGTG GAACT----- ---------- ----------
IbA10G2_AY262031              -----GAGGA AGGCACTGAA GACAATGAAC AAGCCGATGA ------GAGT GCTACCCAAC
IIeA12G1_AY382675             -----GAAGG AACCGGAGAA AATGGTGAAG AGATCGAAGG ------TACT GTTTCCCAAC
IkA15G1_KJ941148              --------GA AGAAAGTGAG GACAGTACTC AATCTAGTGC TGC---TGCT GATTCCCAAA

                              ....|....| ....|....| ....|....| ....|....| ....|....| ....|....|
                                     490        500        510        520        530        540
IiA17_KF679724_               CTTCTGCTCC AGCTCAAGAA TCCGAGAAA- -----ACCGA ATCCACAGAA ---ACTGCTC
25132-IiA17                   CTTCTGCTCC AGCTCAAGAA TCCGAGAAA- -----ACCGA ATCCACAGAA ---ACTGCTC
IIoA13G1_KC885906             --AATACTCC ATCTCAAGGT TCGGACACAG CT---ACCGA AACCACAGAA ---GCTGCTC
25093-IIoA14G1                --AATACTCC ATCTCAAGGT TCGGACACAG CT---ACCGA AACCACAGAA ---GCTGCTC
```

```
IIpA9_KC885904_            --AATACTCC ATCTCAAGGT TCGGACACTG CC---GCCGA AACCACAGAA ---GCTACTC
IInA8_FJ897787             --AATACTCC ATCTCAAGAT TCGGACAAAA CT---ACCGA AACCACAGAA ---GCTACTC
IIdA19G1_KJ802724          --AATACTCC AGCTCAAACT GAAGGCACAA CT---ACCGA AACCACAGAA ---GCTGCTC
25883-IIdA19G1             --AATACTCC AGCTCAAACT GAAGGCACAA CT---ACCGA AACCACAGAA ---GCTGCTC
IIbA14_AF402285            CCACTACTCC AGCTCAAAGT GAAGGCACAA TT---ACCGA AACCACAGAA ---GCTGCTC
IIkA14_AB237137            CTTCCGCCCC AACTCAAG-T TCTGACGCAA CT---ACCGA AACCACAGAA ---GCTGCTC
IIaA15G2R1_JF727769        CCACTACTCC AGCTCAAAGT GAAGGCGCAA CT---ACCGA AACCATAGAA ---GCTACTC
26239-In                   --GATGCTCC ATCTCAAGGT TCTGACACAA TC---ACCGA GTCCACACAA ---ACTACTC
IoA15_KX926458             --GATGCTCC ATCTCAAGGT TCTGACACAA CC---ACCGA GTCCACACAA ---ACTACTC
24965-InA14                --GATGCTCC ATCTCAAGGT TCTGACACAA TC---ACCGA GTCCACACAA ---ACTACTC
26450-InA17                --GATGCTCC ATCTCAAGGT TCTGACACAA TC---ACCGA GTCCACACAA ---ACTACTC
26114-InA26                --GATGCTCC ATCTCAAGGT TCTGACACAA TC---ACCGA GTCCACACAA ---ACTACTC
IdA22_GU214353             --GGTGCTCC ATCTCAAGGT TCTGACAAAA CT---ACCGA GTCCACACAA ---ACTACTC
IhA14G1_FJ971716_          --GTTACCCC ATCTCAAGAT GCCAGCACAG CA---ACCGA GTCCACACCA ---GCTACTC
IjA14_JF681174             CCGCTGCTTC ATCTCAAGAT ACCAGCACAG CTACCACCGA ACCCACAGAA ---ACCACTT
IaA23R4_AF164502           --GTTACTCC ATCTGAAAGT GCCGGCACAG CT---ACCGA GTCCACAGCA ACTACTACTC
24972-ImA18                --GTTACTCC ATCTGAAAGT GCCGGCACAG CT---ACCGA GTCCACAGCA ACTACTACTC
IgA24_EF208067             --GTTACCCC ATCTCAAGAT GCCGGCACAA CA---GCCGA GTCCACACGA ---ACTACTC
IfA19G1R5_AF440638         CTGTCACCCC AACTCAA--- ---------- ---------- ---------- ---GCTACTC
IIcA5G3b_AF164501          CTAGTACCCC AACTCAAGCT GCTGAAAAGG AG---CCCGA AACTCCAGAA ---TCTACTC
IeA11G3T3_DQ665689         CTAGCACCCA AACTCAAGCT ACTGAAAAAG AA---CCCGG ATCTTCAGAA ---GCTACTC
IlA7G4_KP314263_           CTTCTACTTC AGTTCAAGGC TCCGATAAA- -----ACCGA ATCCACAGAA ---ACTGCTC
C.meleagridis_AB539719     ---------- ---------- ---------- ---------- ---------- ---ACTAATC
IbA10G2_AY262031           CTTCTACCCC AGGTCAAGGC TCCGTTAAA- -----ACCGA ATCCACAGAA ---ACTACTC
IIeA12G1_AY382675          CCACTACTCC AGATCAAGGT GAGAGCGCAA CT---CCCGG ATCCACGGAA ---ACTACTC
IkA15G1_KJ941148           CTTCTGCTCC AGCTCAAGAA TCCGAGAAA- -----ACCGA ATCCACAGAA ---ACTGCTC
```

```
                           ....|....| ....|....| ....|....| ....|....| ....|....| ....|....|
                                  550        560        570        580        590        600
IiA17_KF679724_            CAAAGGAAGA GTGCGGTACT TCATTTGTTA TGTGGTTTGG AGAGGGTGTT CCAGTTGCAA
25132-IiA17                CAAAGGAAGA GTGCGGTACT TCATTTGTTA TGTGGTTTGG AGAGGGTGTT CCAGTTGCAA
IIoA13G1_KC885906          CAAAGAAAGA GTGCGGTACT TCATTTGTAA TGTGGTTCGG AGAAGGTACC CCAGTTGCGA
25093-IIoA14G1             CAAAGAAAGA GTGCGGTACT TCATTTGTAA TGTGGTTCGG AGAAGGTACC CCAGTTGCGA
IIpA9_KC885904_            CAAAGAAAGA GTGCGGTACT TCATTTGTTA TGTGGTTCGG ACAGGGTGTT CCAGTTGCAA
IInA8_FJ897787             CAAAGAAAGA GTGCGGTACT TCATTTGTTA TGTGGTTCGG GGAGGGTGTT CCAGTTGCAA
IIdA19G1_KJ802724          CAAAGAAAGA GTGCGGTACT TCATTTGTTA TGTGGTTCGG AGAGGGTGTT CCAGTTGCAT
25883-IIdA19G1             CAAAGAAAGA GTGCGGTACT TCATTTGTTA TGTGGTTCGG AGAGGGTGTT CCAGTTGCAT
IIbA14_AF402285            CAAAGAAAGA GTGCGGTACT TCATTTGTTA TGTGGTTCGG AGAAGGTACC CCAGTTGCGA
IIkA14_AB237137            CAAAGAAAGA GTGCGGTACT TCATTTGTTA TGTGGTTCGG AGAAGGTACC CCAGTTGCGA
IIaA15G2R1_JF727769        CAAAAGAAGA ATGCGGCACT TCATTTGTAA TGTGGTTCGG AGAAGGTACC CCAGCTGCGA
26239-In                   CAAAGGAAGA GTGCGGTACT TCGTTTGTAA TGTGGTTCGG TGAAGGTACC CCGGTTGCGA
IoA15_KX926458             CAAAGGAAGA GTGCGGTACT TCGTTTGTAA TGTGGTTCGG TGAAGGTACC CCGGTTGCGA
24965-InA14                CAAAGGAAGA GTGCGGTACT TCGTTTGTAA TGTGGTTCGG TGAAGGTACC CCGGTTGCGA
26450-InA17                CAAAGGAAGA GTGCGGTACT TCGTTTGTAA TGTGGTTCGG TGAAGGTACC CCGGTTGCGA
26114-InA26                CAAAGGAAGA GTGCGGTACT TCGTTTGTAA TGTGGTTCGG TGAAGGTACC CCGGTTGCGA
IdA22_GU214353             CAAAGGAAGA GTGCGGTACT TCGTTTGTAA TGTGGTTCGG TGAAGGTACC CCGGTTGCGA
IhA14G1_FJ971716_          CAAAGGAAGG ATGCGGTACT TCATTCGTAA TGTGGTTCGA TAAAGGTACC CCGGTTGCGA
IjA14_JF681174             CAAAGGAAGG ATGCGGTACT TCATTTGTAA TGTGGTTCGA TAAAGGTACC CCGGTTGCGA
IaA23R4_AF164502           CAAAGGAAGA ATGTGGTACT TCATTTGTCA TGTGGTTCGA GAAAGGCACC CCGGTTGCGA
24972-ImA18                CAAAGGAAGA ATGTGGTACT TCATTTGTCA TGTGGTTCGA GCAAGGCACC CCGGTTGCGA
IgA24_EF208067             CAAAGGAAGG ATGCGGTACT TCATTTGTAA TGTGGTTCGA TAAAGGTACC CCGGTTGCGA
IfA19G1R5_AF440638         CAAAGGAAGA ATGTGGTACT TCATTCATAA TGTGGTTCGG AGAAGGTACT CCAGCCACAA
IIcA5G3b_AF164501          CAAAGGAAGA ATGTGGTACT TCATTTATAA TGTGGTTCGG AGAAGGTACT CCAGCCACAA
IeA11G3T3_DQ665689         CAAAGGAAGA GTGCGGTACT TCATTTGTAA TGTGGTTCGG ACAGGGTGTT CCAGTTGTAA
IlA7G4_KP314263_           CAAAGGAGAA GTGCGGTACT TCATTTGTTA TGTGGTTCGG AGAGGGTGTT CCAGTCGCAA
C.meleagridis_AB539719     CAAGTGAA-- -TGTGGTACT TCATTTGTCA TGTGGTTCGG AGAAGGTACT CCAGTTGCAA
IbA10G2_AY262031           CAAAGGAGAA GTGCGGTACT TCATTTGTTA TGTGGTTCGG ACAGGGTGTT CCAGTCGCAA
IIeA12G1_AY382675          CAAAGGAAGA ATGCGGTACT TCATTTGTAA TGTGGTTCGG AGAAGGTACC CCAGTTGCGA
IkA15G1_KJ941148           CAAAGGAAGA GTGCGGTACT TCATTTGTTA TGTGGTTTGG AGAGGGTGTT CCAGTTGCAA
```

```
                           ....|....| ....|....| ....|....| ....|....| ....|....| ....|....|
                                  610        620        630        640        650        660
IiA17_KF679724_            CTTTGAAGTG TGGTGACTAT ACTATGGTCT ATGCACCAGA AAATGGCAAA ACAGATCCCG
25132-IiA17                CTTTGAAGTG TGGTGACTAT ACTATGGTCT ATGCACCAGA AAATGGCAAA ACAGATCCCG
IIoA13G1_KC885906          CCTTGAAGTG TGGTGACTAC ACTATGGTCT ATGCACCAGA AAAAGACAAA ACAGATCCCG
25093-IIoA14G1             CCTTGAAGTG TGGTGACTAC ACTATGGTCT ATGCACCAGA AAAAGACAAA ACAGATCCCG
IIpA9_KC885904_            CTTTGAAGTG TGGCGACTAT ACTATGGTCT ATGCACCAGA AAAGGACAAA ACAGATCCCG
IInA8_FJ897787             CTTTGAAGTG TGGCGATTAT ACTATGGTCT ATGCACCAGA AAAGGACAAA ACAGATCCCG
IIdA19G1_KJ802724          CTTTGAAGTG TGGCGACTAT ACTATGGTCT ATGCACCAGA AAAGGACAAA ACAGATCCCG
```

```
25883-IIdA19G1              CTTTGAAGTG TGGCGACTAT ACTATGGTCT ATGCACCAGA AAAGGACAAA ACAGATCCCG
IIbA14_AF402285             CCTTGAAGTG TGGCGACTAT ACTATGGTCT ATGCACCAGA AAAGGACAAA ACAGATCCCG
IIkA14_AB237137             CCTTGAAGTG TGGCGACTAT ACTATGGTCT ATGCACCAGA AAAGGACAAA ACAGATCC--
IIaA15G2R1_JF727769         CATTGAAGTG TGGTGCCTAC ACTATCGTCT ATGCACCTAT AAAAGACCAA ACAGATCCCG
26239-In                    CCTTGAAGTG TGGTGGTTAC ACTATCGTCT ATGCACCTGT AAAGGATCAA ACAAATCCCG
IoA15_KX926458              CCTTGAAGTG TGGTGGTTAC ACTATCGTCT ATGCACCTGT AAAGGATCAA ACAAATCCCG
24965-InA14                 CCTTGAAGTG TGGTGGTTAC ACTATCGTCT ATGCACCTGT AAAGGATCAA ACAAATCCCG
26450-InA17                 CCTTGAAGTG TGGTGGTTAC ACTATCGTCT ATGCACCTGT AAAGGATCAA ACAAATCCCG
26114-InA26                 CCTTGAAGTG TGGTGGTTAC ACTATCGTCT ATGCACCTGT AAAGGATCAA ACAAATCCCG
IdA22_GU214353              CCTTGAAGTG TGGTGGTTAC ACTATCGTCT ATGCACCTGT AAAGGATCAA GCAAATCCCG
IhA14G1_FJ971716_           CCTTGAAGTG TGATGGTTAC ACTATCGTCT ATGCACCTAT AACAGGTCAA ACAGATCCCG
IjA14_JF681174              CATTGAAGTG TGGTGGTTAC ACTATCGTCT ATGCACCTGT AACAGGTCAA ACAGATCCCG
IaA23R4_AF164502            CCTTGAAGTG TGGTGATTAC ACTATCGTCT ATGCACCTAT AAAAGATCAA ACAGATCCCG
24972-ImA18                 CCTTGAAGTG TGGTGATTAC ACTATCGTCT ATGCACCTAT AAAAGATCAA ACAGATCCCG
IgA24_EF208067              CCTTGAAGTG TGGTGATTAC ACTATCGTCT ATGCACCTAT AAAAGATCAA ACAGATCCCG
IfA19G1R5_AF440638          CTTTGAAGTG TGGTGGTTAC ACTATCGTCT ATGCACCAGA AAAGAACAAT AGAGAACCCG
IIcA5G3b_AF164501           CTTTGAAGTG CGGTGGCTAC ACTATCGTCT ATGCACCAGA AAAGGATAAT AAAGAACCCG
IeA11G3T3_DQ665689          CTTTGAAGTG TGGTGGTTAT ACTATGGTCT ATGCACCAGA AAATGGCAAA ACAGATCCCG
IlA7G4_KP314263_            CTTTGAAGTG CGGTGACTAT ACTATGGTCT ATGCACCAGA AAAGGACAAA ACAGATCCCG
C.meleagridis_AB539719      CTTTGAAATG CGGTGATTAT ACTATCGTCT ATGCACCTGA AAGTGGCAAA ACAGATCCCG
IbA10G2_AY262031            CTTTGAAGTG CGGTGACTAT ACTATGGTCT ATGCACCAGA AAAGGACAAA ACAGATCCCG
IIeA12G1_AY382675           CCTTGAAGTG TGGTGGTTAC ACTATCGTCT ATGCACCTGT AAAGGAACAA ACAAATCCCG
IkA15G1_KJ941148            CTTTGAAGTG TGGTGACTAT ACTATGGTCT ATGCACCAGA AAGTGGCAAA ACAGATCCCG


                            ....|....| ....|....| ....|....| ....|....| ....|....| ....|....|
                                   670        680        690        700        710        720
IiA17_KF679724_             CACCAAGATA TATCTCTGGC GCAGTTTCAA CCGTAACCTT C---GAAAA- --AGAAGATA
25132-IiA17                 CACCAAGATA TATCTCTGGC GCAGTTTCAA CCGTAACCTT C---GAAAA- --AGAAGATA
IIoA13G1_KC885906           AACCAAGATA TATCTCAGGC GAAGTTACAA GCGTAACCTT T---GAAAA- --ACAAGACA
25093-IIoA14G1              AACCAAGATA TATCTCAGGC GAAGTTACAA GCGTAACCTT T---GAAAA- --ACAAGACA
IIpA9_KC885904_             CACCAAGATA TATCTCTGGC GAAGTTACAG AAGTAACCTT T---GAAAA- --ACAAGAGA
IInA8_FJ897787              CACCAAGATA TATCTCTGGC GAAGTTACAG GAGTAACCTT T---GAAAA- --ACAAGATA
IIdA19G1_KJ802724           CACCAAGATA TATCTCTGGT GAAGTTACAT CTGTAACCTT T---GAAAA- --ACAAGAGA
25883-IIdA19G1              CACCAAGATA TATCTCTGGT GAAGTTACAT CTGTAACCTT T---GAAAA- --ACAAGAGA
IIbA14_AF402285             CACCAAGAA- ---------- ---------- ---------- ---------- ----------
IIkA14_AB237137             ---------- ---------- ---------- ---------- ---------- ----------
IIaA15G2R1_JF727769         CACCAAGATA TATCTCTGGT GAAGTTACAT CTGTAACCTT T---GAAAA- --GAGTGATA
26239-In                    CACCAAGATA TATCTCTGGT GAAGTAAAGA ATGTATCCTT C---CAAAAA GAAAGTGATA
IoA15_KX926458              CACCAAGATA TATCTCTGGT GAAGTAAAGA ATGTATCCTT C---CAAAAA GAAAGTGATA
24965-InA14                 CACCAAGATA TATCTCTGGT GAAGTAAAGA ATGTATCCTT C---CAAAAA GAAAGTGATA
26450-InA17                 CACCAAGATA TATCTCTGGT GAAGTAAAGA ATGTATCCTT C---CAAAAA GAAAGTGATA
26114-InA26                 CACCAAGATA TATCTCTGGT GAAGTAAAGA ATGTATCCTT C---CAAAAA GAAAGTGATA
IdA22_GU214353              CACCAAGATA TATCTCTGGT GAAGTAAAGA ATGTATCCTT C---CAAAAA GAAAGTGATA
IhA14G1_FJ971716_           CACCAAGATA TATCTCTGGT GAAGTTACAT CTGTAACCTT T---GAAGA- --AAGTGATA
IjA14_JF681174              CACCAAGATA TATCTCTGGT GAAGTTACAT CCGTAACCTT T---GAAGA- --CAGTGATA
IaA23R4_AF164502            CACCAAGATA TATCTCTGGT GAAGTTACAT CTGTATCCTT T---GAAAA- --GAGTGAAA
24972-ImA18                 CACCAAGATA TATCTCTGGT GAAGTTACAT CTGTATCCTT T---GAAAA- --GAGTGAAA
IgA24_EF208067              CACCAAGATA TATCTCTGGT GAAGTTACAT CTGTATCCTT T---GAAAA- --GAGTGAAA
IfA19G1R5_AF440638          CACCAAGATA TATCTCTGGT GAGGTTAAGG CTGTAACTTT T---GAAAAG GGAAATGACA
IIcA5G3b_AF164501           CACCAAGATA CATCTCTGGT GATGTTAAGG CTGTAACCTT T---GAAAAG GGAGAAGATA
IeA11G3T3_DQ665689          CACCAAGATA TATCTCTGGT AAAGTTTCAA CCGTAGACTT T---GAAAA- --ACAAGATA
IlA7G4_KP314263_            CACCAAGATA TATCTCTGGT GAAGTTACAA CCGTAACCTT T---GATAA- --TCAAAATA
C.meleagridis_AB539719      CTCCAAAATA TATCTCTGGT GACGTTAAGG CTGTAACCTT TGAAAAAGAA TCAAGTTCTA
IbA10G2_AY262031            CACCAAGATA TATCTCTGGT GAAGTTACAA CCGTAACCTT T---GATAA- --ACAAGAGA
IIeA12G1_AY382675           CACCAAGAT- ---------- ---------- ---------- ---------- ----------
IkA15G1_KJ941148            CACCAAGATA TATCTCTGGC GCAGTTTCAA CCGTAACCTT C---GAAAA- --AGAAGATA


                            ....|....| ....|....| ....|....| ....|....| ....|....| ....|....|
                                   730        740        750        760        770        780
IiA17_KF679724_             GTACAGTTAA AATCAAGGTT AATGGCGTAG AGTTCAGCAC TCTCTCTACT AGCTCAAGTG
25132-IiA17                 GTACAGTTAA AATCAAGGTT AATGGCGTAG AGTTCAGCAC TCTCTCTACT AGCTCAAGTG
IIoA13G1_KC885906           GTACAGTTAC AATCAAGGTT AATAATGTAG CGTTCAGTAC TCTCTCTACT AGCTCAAGTA
25093-IIoA14G1              GTACAGTTAC AATCAAGGTT AATAATGTAG CGTTCAGTAC TCTCTCTACT AGCTCAAGTA
IIpA9_KC885904_             GCACAGTTAC AATCAAGGTT AATAATGTAG AGTTCAGCAC TCTTTCTACT AGCTCAAGTA
IInA8_FJ897787              GCACAGTTAA AATCAAGGTT AATGATGTGG AGTTCGGCAC TCTTTCTACT AGCTCAAGTA
IIdA19G1_KJ802724           GCACAGTTAC AATCAAGGTT AATAATGTAG AGTTCAGCAC TCTTTCTACT AGCTCAAGTA
25883-IIdA19G1              GCACAGTTAC AATCAAGGTT AATAATGTAG AGTTCAGCAC TCTTTCTACT AGCTCAAGTA
IIbA14_AF402285             ---------- ---------- ---------- ---------- ---------- ----------
IIkA14_AB237137             ---------- ---------- ---------- ---------- ---------- ----------
```

```
IIaA15G2R1_JF727769        ATACAGTTAA AATCAAGGTT AACGGTCAGG ATTTCAGCAC TCTCTCTGCT AATTCAAGTA
26239-In                   ATACAATTAA AATCAAGGTT GACGGTCAGG ATTTCAGCAC TCTCTCTGCT AGCTCAAGTA
IoA15_KX926458             ATACAATTAA AATCAAGGTT GACGGTCAGG ATTTCAGCAC TCTCTCTGCT AGCTCAAGTA
24965-InA14                ATACAATTAA AATCAAGGTT GACGGTCAGG ATTTCAGCAC TCTCTCTGCT AGCTCAAGTA
26450-InA17                ATACAATTAA AATCAAGGTT GACGGTCAGG ATTTCAGCAC TCTCTCTGCT AGCTCAAGTA
26114-InA26                ATACAATTAA AATCAAGGTT GACGGTCAGG ATTTCAGCAC TCTCTCTGCT AGCTCAAGTA
IdA22_GU214353             ATACAATTAA AATCAAGGTT GACGGTCAGG ATTTCAGCAC TCTCTCTGCT AGCTCAAGTA
IhA14G1_FJ971716_          GTACAGTTAA AATCAAGGTT AACGGTCAGG AATTCAGCAC TCTCTCTGCT AGCTCAAGTA
IjA14_JF681174             GTACAGTTAA AATCAAGGTT AACGGTCATG AGTTCAGCAC TCTCTCTGCT AGCTCAAGTA
IaA23R4_AF164502           GTACAGTTAC AATCAAGGTT AATGGTAAAG AGTTCAGCAC TCTCTCTGCT AACTCAAGTA
24972-ImA18                GTACAGTTAC AATCAAGGTT AATGGTAAAG AGTTCAGCAC TCTCTCTGCT AACTCAAGTA
IgA24_EF208067             GTACAGTTAC AATCAAGGTT AATGGTAAAG AGTTCAGCAC TCTCTCTGCT AACTCAAGTA
IfA19G1R5_AF440638         ATACAATTAA AATCAAGGTT GACGGTAAGG AGTTTAGCAC TCTCTCTACT AGCTCAAGTA
IIcA5G3b_AF164501          ATACAGTTAA AATCAAGGTT GATGGTAAGG AGTTCAGTAC TCTCTCTTCT AGCTCAAGCA
IeA11G3T3_DQ665689         GTACAGTTAA AATCAAGGTT AATGGTTCGG AGTTCAGCAC TCTCTCTACT AGCTCAAGTA
IlA7G4_KP314263_           ATACAGTTAC AATCAAGGTT AATGGTAAAG AGTTCAGCAC TCTCTCTGCT GACTCAAGTA
C.meleagridis_AB539719     ATACAATAAA AATCAAGGTT GACGGTAAGG AGCTCAGCAC TCTCTCTACT AATTCAAATT
IbA10G2_AY262031           GTACAGTTAC AATCAAGGTT AATAATGTAG AGTTCGGCAC TCTCTCTACT AGCTCAAGTA
IIeA12G1_AY382675          ---------- ---------- ---------- ---------- ---------- ----------
IkA15G1_KJ941148           GTACAGTTAA AATCAAGGTT AATGGCGTAG AGTTCAGCAC TCTCTCTACT AGCTCAAGTG

                           ....|....| ....|....| ....|....| ....|....| ....|....| ....|....|
                                  790        800        810        820        830        840
IiA17_KF679724_            AACCAACTAA AAATAGCGG- --ATCT---G AGAGCC---A GGCTCAATCA AGATCAAGAA
25132-IiA17                AACCAACTAA AAATAGCGG- --ATCT---G AGAGCC---A GGCTCAATCA AGATCAAGAA
IIoA13G1_KC885906          ATCCAACTGA AAATACCGA- --ATCT---G CAAGCC---A GACTCCATCG AGATCAAGAA
25093-IIoA14G1             ATCCAACTGA AAATACCGA- --ATCT---G CAAGCC---A GACTCCATCG AGATCAAGAA
IIpA9_KC885904_            GTCCAACTGA AAATAGCGG- --ATCT---G CAGGTC---A TGTTTCATCA AGATCAAGAA
IInA8_FJ897787             GTCCAACTGA AAATAGCGG- --ATCT---G CAGGTC---A GGTTCAATCA AGATCAAGAA
IIdA19G1_KJ802724          GTCCAACTGA AAATAGCGG- --ATCT---G CAGGTC---A GGTTCCATCA AGATCAAGAA
25883-IIdA19G1             GTCCAACTGA AAATAGCGG- --ATCT---G CAGGTC---A GGTTCCATCA AGATCAAGAA
IIbA14_AF402285            ---------- ---------- ---------- ---------- ---------- ----------
IIkA14_AB237137            ---------- ---------- ---------- ---------- ---------- ----------
IIaA15G2R1_JF727769        GTCCAACTGA AAATGGCGG- --ATCT---G CGGGTC---A GGCTTCATCA AGATCAAGAA
26239-In                   GTCCAACCGA AAATAAAGGT GAGTCT---G GCAATC---A GGTTGAGTCA AGATCAAGAA
IoA15_KX926458             GTCCAACCGA AAATAAAGGT GAGTCT---G GCAATC---A GGTTGAGTCA AGATCAAGAA
24965-InA14                GTCCAACCGA AAATAAAGGT GAGTCT---G GCAATC---A GGTTGAGTCA AGATCAAGAA
26450-InA17                GTCCAACCGA AAATAAAGGT GAGTCT---G GCAATC---A GGTTGAGTCA AGATCAAGAA
26114-InA26                GTCCAACCGA AAATAAAGGT GAGTCT---G GCAATC---A GGTTGAGTCA AGATCAAGAA
IdA22_GU214353             GTCCAACCGA AAATAAAGGT GAGTCT---G GCAATC---A GGTTGAGTCA AGATCAAGAA
IhA14G1_FJ971716_          GTCCAACTGA AAATAAAGGT GAGTCTAGTG ACGGTC---A GGTTGAGTCA AGATCAAGAA
IjA14_JF681174             GTCCAAGTGA AAATAATGGT GGATCCGGTG GTAGTCAGGA GGTTAAGTCA AGATCAAGAA
IaA23R4_AF164502           GTCCAACTAA AGATAACGGT GAATCTAGTA ACAGTC---A GGTTCAATCA AGATCAAGAA
24972-ImA18                GTCCAACTAA AGATAACGGT GAATCTAGTG ACAGTC---A GGTTCAATCA AGATCAAGAA
IgA24_EF208067             GTCCAACTAA AGATAACGGT GAATCTAGTG ACAGTC---A GGTTCAATCA AGATCAAGAA
IfA19G1R5_AF440638         GTCCAACTGT TAATAACGGT GGATCT---G ATGTCC---A AGCTAAATCA ATATCAAAGA
IIcA5G3b_AF164501          GTCCAACTGA AAATAACGG- --ATCT---A CGGGCC---A GGTTGCATCA AGATCAAGAA
IeA11G3T3_DQ665689         ATCCAACTGA AAATAGCGG- --ATCT---G AGAGCC---A GGCTCAATCA AGATCAAGAA
IlA7G4_KP314263_           GTCCAACTAA AGATAACGGT GAATCTAGTG ACAGTA---A GGTTCAATCA AGATCAAGAA
C.meleagridis_AB539719     CCCCAACAGA AAATGACACT GCGTCTGAGG AAAGCT---T ATCTAGATCG CGATCAAAAA
IbA10G2_AY262031           AACCAACTGA AAATAAAGGT GAGTCT---A GCGATC---A GGTTGGGTCA AGATCAAGAA
IIeA12G1_AY382675          ---------- ---------- ---------- ---------- ---------- ----------
IkA15G1_KJ941148           AACCAACTAA AAATAGCGG- --ATCT---G AGAGCC---A GGCTCAATCA AGATCAAGAA

                           ....|....| ....|....| ....|....| ....|....| ....|....| ....|....|
                                  850        860        870        880        890        900
IiA17_KF679724_            GATCACTCAC AGAGGAGGAT AGTGCTGAAA CTGCTGCAAC TGTCGATTTA ATTGCCTTCA
25132-IiA17                GATCACTCAC AGAGGAGGAT AGTGCTGAAA CTGCTGCAAC TGTCGATTTA ATTGCCTTCA
IIoA13G1_KC885906          GATCACTCTC AGAGGAAACT AG---TGAAG CTGCTGCAAC CGTCGATTTG TTTGCCTTTA
25093-IIoA14G1             GATCACTCTC AGAGGAAACT AG---TGAAG CTGCTGCAAC CGTCGATTTG TTTGCCTTTA
IIpA9_KC885904_            GATCACTCTC AGAGGAGGCT AG---TGAAA C---TGCAAC CGTCGATTTG TTTGCCTTCA
IInA8_FJ897787             GATCACTCTC AGAGGAGGCT AG---TGAAA C---TGCAAC CGTCGATTTG TTTGCCTTCA
IIdA19G1_KJ802724          GATCACTCTC AGAGGAGGCT AG---TGAAA C---TGCAAC CGTCGATTTG TTTGCCTTCA
25883-IIdA19G1             GATCACTCTC AGAGGAGGCT AG---TGAAA C---TGCAAC CGTCGATTTG TTTGCCTTCA
IIbA14_AF402285            ---------- ---------- ---------- ---------- ---------- ----------
IIkA14_AB237137            ---------- ---------- ---------- ---------- ---------- ----------
IIaA15G2R1_JF727769        GATCACTCTC AGAGGAAACC AG---TGAAG CTGCTGCAAC CGTCGATTTG TTTGCCTTTA
26239-In                   GATCACTCAC AGAGGAAACT AG---TGAAA C---TTCAAC CGTCGATTTG TTTGCCTTTA
IoA15_KX926458             GATCACTCAC AGAGGAAACT AG---TGAAA C---TTCAAC CGTCGATTTG TTTGCCTTTA
```

```
24965-InA14                GATCACTCAC AGAGGAAACT AG---TGAAA C---TTCAAC CGTCGATTTG TTTGCCTTTA
26450-InA17                GATCACTCAC AGAGGAAACT AG---TGAAA C---TTCAAC CGTCGATTTG TTTGCCTTTA
26114-InA26                GATCACTCAC AGAGGAAACT AG---TGAAA C---TTCAAC CGTCGATTTG TTTGCCTTTA
IdA22_GU214353             GATCACTCAC AGAGGAAACT AG---TGAAA C---TGCAAC CGTCGATTTG TTTGCCTTTA
IhA14G1_FJ971716_          GATCACTCAC AGAGGAAACT AG---TGAAA C---TGCAAC CGTCGATTTG TTTGCCTTTA
IjA14_JF681174             GATCACTCAC AGAGGGAGA- -----TGAAA CACCTGCAAC CGTCGATTTG TTTGCCTTCA
IaA23R4_AF164502           GATCACTCGC AGAGGAGAAT GG---TGAAA CAGTTGCAAC AGTTGATTTG TTTGCCTTTA
24972-ImA18                GATCACTCGC AGAGGAGAAT GG---TGAAA CAGTTGCAAC CGTTGATTTG TTTGCCTTTA
IgA24_EF208067             GATCACTCGC AGAGGAGAAT GG---TGAAA CAGTTGCAAC AGTTGATTTG TTTGCCTTTA
IfA19G1R5_AF440638         GATCACTCAC AGAGGAAAGT GA---CGAAG T---TGCGAC CGTCGATTTG TTTGCCTTTA
IIcA5G3b_AF164501          GATCGCTCTC AGAGGAAAAT AG---TGAAA CTGCTGCAAC CGTCGATTTG TTTGCCTTCA
IeA11G3T3_DQ665689         GATCACTCGC AGAGGATGGT AC---TGAGA CTGCTGCAAC CGTCGATTTG ATTGCCTTCA
IlA7G4_KP314263_           GATCACTCGC AGAGGGGAAT GG---TGAAA CAGTTGCAAC CGTTGATTTG TTTGCCTTTA
C.meleagridis_AB539719     GATCACTCTC AGACGC---- -----TGAGA CAACTGGAAC TGTTGATGTG CTCGCCTTTA
IbA10G2_AY262031           GATCACTCAC AGAGGAAACT AG---TGAAA C---TGCAAC CGTCGATTTG TTTGCCTTTA
IIeA12G1_AY382675          ---------- ---------- ---------- ---------- ---------- ----------
IkA15G1_KJ941148           GATCACTCAC AGAGGAGGAT AGTGCTGAAA CTGCTGCAAC TGTCGATTTA ATTGCCCTCA


                           ....|....| ....|....| ....|....| ....|....| ....|....| ....|....|
                                  910        920        930        940        950        960
IiA17_KF679724_            CCCT---CCA TGGTGGTAAA AGAATCGAAG TGGCTGTGCC AAGTGACGAG GATACAAGCA
25132-IiA17                CCCT---CCA TGGTGGTAAA AGAATCGAAG TGGCTGTGCC AAGTGACGAG GATACAAGCA
IIoA13G1_KC885906          CCCT---TGA TGGTGGTAAA AGAATTGAAG TGGCTGTACC AAGCGTCGAA GATGCATCTA
25093-IIoA14G1             CCCT---TGA TGGTGGTAAA AGAATTGAAG TGGCTGTACC AAGCGTCGAA GATGCATCTA
IIpA9_KC885904_            CCCT---TGA TGGTGGTAAA AGAATTGAAG TTGCTGTACC AAGCGTCGAA GATGCATCCA
IInA8_FJ897787             CCCT---TGA TGGTGGTAAA AGAATTGAAG TTGCTGTACC AAGCGTCGAA GATGCATCCA
IIdA19G1_KJ802724          CCCT---TGA TGGTGGTAAA AGAATTGAAG TTGCTGTACC AAGCGACGAA GATGCATCTA
25883-IIdA19G1             CCCT---TGA TGGTGGTAAA AGAATTGAAG TTGCTGTACC AAGCGACGAA GATGCATCTA
IIbA14_AF402285            ---------- ---------- ---------- ---------- ---------- ----------
IIkA14_AB237137            ---------- ---------- ---------- ---------- ---------- ----------
IIaA15G2R1_JF727769        CCCT---TGA TGGTGGTAAA AGAATTGAAG TGGCTGTACC AAACGTCGAA GATGCATCTA
26239-In                   CCCT---TAG TGGTGGTAAG AGAATTGAAG TGGCTGTGCC AAACGCCGAA GAAACATCGA
IoA15_KX926458             CCCT---TAG TGGTGGTAAG AGAATTGAAG TGGCTGTGCC AAACGCCGAA GAAACATCGA
24965-InA14                CCCT---TAG TGGTGGTAAG AGAATTGAAG TGGCTGTGCC AAACGCCGAA GAAACATCGA
26450-InA17                CCCT---TAG TGGTGGTAAG AGAATTGAAG TGGCTGTGCC AAACGCCGAA GAAACATCGA
26114-InA26                CCCT---TAG TGGTGGTAAG AGAATTGAAG TGGCTGTGCC AAACGCCGAA GAAACATCGA
IdA22_GU214353             CCCT---TAA TGGTGGTAAG AGAATTGAAG TGGCTGTGCC AAACGCCGAA GAAACATCGA
IhA14G1_FJ971716_          CCCT---TGA TGGTGGTAAG AGAATTGAAG TGGCTGTACC AAACGCCGCA GAAACATCCA
IjA14_JF681174             CCCTTGGTGA TGGTGGTAAA AGAATTGAAG TAGCTGTACC AAGCAACGAA AATGCATCCC
IaA23R4_AF164502           CTCT---TGA TGGTGGTAGA AGAATTGAAG TGGCTGTGCC AAAGGACGAA AATGCAGACA
24972-ImA18                CTCT---TGA TGGTGGTAAA AGAATTGAAG TGGCTGTGCC AAAGGACGAA AATGCAGACA
IgA24_EF208067             CTCT---TGA TGGTGGTAGA AGAATTGAAG TGGCTGTGCC AAAGGACGAA AATGCAGACA
IfA19G1R5_AF440638         CTCT---TGA TGGTGGTAAA AGAATTGAAG TGGCTGTACC AAGTGTCGAT GATGTATCCA
IIcA5G3b_AF164501          CCCT---TGA TGGTGGCCGA AGAATTGAAG TTGCTGTACC CAGCGTCGAA GATGCAACCA
IeA11G3T3_DQ665689         CCCT---TCA AGGTGGTAAA AGAATCGAAG TCGCTGTGCC AAGTGACGAA GATGTATCCA
IlA7G4_KP314263_           CCCT---TGA TGGTGGTAAA AGAATTGAAG TGGCTGTACC AAAAGACGAA GCTGTATCCA
C.meleagridis_AB539719     CCCT---CCA AGGTGGTAAA AGAATTGAAG TGGCTGTACC AAATGCCAGT GATTCAGCCC
IbA10G2_AY262031           CCCT---TGA TGGTGGTAAA AGAATTGAAG TGGCTGTACC AAGTGACGAA GATGTATCCA
IIeA12G1_AY382675          ---------- ---------- ---------- ---------- ---------- ----------
IkA15G1_KJ941148           CCCT---CCG TGGTGGTAAA AGAATCGAAG TGGCTGTGCC AAGTGACGAG GATACAAGCA


                           ...

IiA17_KF679724_            AGA
25132-IiA17                AGA
IIoA13G1_KC885906          AAA
25093-IIoA14G1             AAA
IIpA9_KC885904_            AAA
IInA8_FJ897787             AAA
IIdA19G1_KJ802724          AAA
25883-IIdA19G1             AAA
IIbA14_AF402285            ---
IIkA14_AB237137            ---
IIaA15G2R1_JF727769        AAA
26239-In                   AAA
IoA15_KX926458             AAA
24965-InA14                AAA
26450-InA17                AAA
26114-InA26                AAA
```

**IdA22_GU214353**	AAA
**IhA14G1_FJ971716_**	AAA
**IjA14_JF681174**	AAA
**IaA23R4_AF164502**	AAA
**24972-ImA18**	AAA
**IgA24_EF208067**	AAA
**IfA19G1R5_AF440638**	AGA
**IIcA5G3b_AF164501**	AAA
**IeA11G3T3_DQ665689**	AGA
**IlA7G4_KP314263_**	AGA
**C.meleagridis_AB539719**	AAA
**IbA10G2_AY262031**	AGA
**IIeA12G1_AY382675**	---
**IkA15G1_KJ941148**	AGA
